# Synthesis and photoinitiated thiol–ene reactions of *exo*-mannals – a new route to *C*-β-d-mannosyl derivatives†

**DOI:** 10.1039/d0ra07115c

**Published:** 2020-09-22

**Authors:** János József, Nóra Debreczeni, Dániel Eszenyi, Anikó Borbás, László Juhász, László Somsák

**Affiliations:** Department of Organic Chemistry, University of Debrecen PO Box 400 H-4002 Debrecen Hungary juhasz.laszlo@science.unideb.hu somsak.laszlo@science.unideb.hu +36-52-512900/22464, +36-52-512900/22348; Department of Pharmaceutical Chemistry, University of Debrecen PO Box 400 H-4002 Debrecen Hungary; University of Debrecen, Doctoral School of Chemistry PO Box 400 H-4002 Debrecen Hungary; HAS-UD Molecular Recognition and Interaction Research Group, University of Debrecen Egyetem tér 1 Debrecen 4032 Hungary

## Abstract

Syntheses of acyl protected *exo*-mannal derivatives were developed starting from *O*-peracylated mannopyranoses *via* the corresponding anhydro-aldose tosylhydrazones under modified Bamford–Stevens conditions. The synthesis of analogous *O*-peralkylated (benzylated and isopropylenated) derivatives was carried out from pyranoid and furanoid mannonolactones using methylene transfer reagents. Photoinitiated thiol–ene additions of these *exo*-mannals resulted in the corresponding *C*-(mannopyranosyl/mannofuranosyl)methyl sulfides in medium to good yields with exclusive regio- and β(d) stereoselectivities.

## Abstract 2

A synthetic procedure was elaborated for *O*-peracylated exo-mannals. Thiol-ene additions to pyranoid and furanoid exo-mannals gave mannosylmethyl sulfide type adducts with exclusive regio- and β(d)-stereoselectivities including disaccharide mimetics.

## Graphical abstract

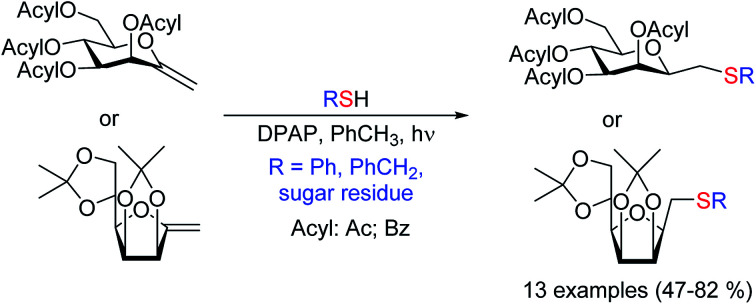

## 1. Introduction

d-Mannose occurs in microbes, plants, animals in free form, but more often as a component of glycans or glycoproteins. Several articles were published in recent years about the metabolic study and biological function of mannose and mannose containing glycoconjugates demonstrating that the therapeutic applications of these derivatives receive increasing attention.^1–4^ Mannose can be used as a drug in the case of specific bacterial infections, but it can be lethal or teratogenic, too.^5–7^ The mannose binding lectins (MBL) of the human cells play a central role in innate immunity by the interaction with surface sugars of a wide series of microorganisms, but this specific interaction can also be used for selective delivery of anti-cancer drugs, using glycosylated (mannosylated) bioconjugates.^8–10^

The *O*-glycosidic bond in natural glycosides is characterized with low hydrolytic and/or enzymatic stability but by replacing the glycosidic oxygen with other atoms (C, N, S)^11^ or groups (S–S, S–Se, SO_2_–N, and N–C(=O)–N),^12^ more stable molecules can be synthesized with similar biological activity. These molecules are the glycomimetics,^11^ which are frequently used as leads of drug discovery.

Several routes have been published in the literature for the synthesis of *C*-glycosyl derivatives but the yield and stereoselectivity of these reactions are highly dependent on the circumstances, protecting groups and configuration of the starting compounds.^13–15^*C*-Mannosyl derivatives received special attention as summarized in a recent review.^16^

Photoinitiated thiol–ene additions, also called thio-click reactions are widely used in synthetic organic chemistry and material science for the synthesis of sulfur containing compounds.^17–20^ In carbohydrate chemistry, the sugar moiety can be used both as a thiol or an alkene components, and such transformations show excellent regio- and stereoselectivities.^21^ The hydrothiolation of *exo*-glycals allows the synthesis of novel, glycosylmethyl sulfide (Gly–CH_2_–SR) type mimetics with very high or exclusive β-selectivity.^22–25^

Based on the above experiences we set out to study the thiol–ene additions with various *exo*-mannals with the expectation that the β-stereoselectivity observed with other *exo*-glycal configurations will be maintained here as well. This synthetic route can provide mimetics of *O*-β-d-mannosyl derivatives whose syntheses are otherwise very challenging tasks.^26^

## 2. Results

### 2.1. Synthesis of *exo*-mannal derivatives

Several methods are known in the literature for the synthesis of *exo*-glycal derivatives possessing different protecting groups.^27,28^ In the case of base stable ether or acetal type protection, they can be synthesized from the corresponding aldonolactones, using well-known olefination methods with the Petasis and the Tebbe reagents^29–31^ or Julia/modified Julia olefinations^32,33^ under strongly basic conditions. We have developed a simple method for the preparation of *exo*-glycals with ester type protection of hydroxyl groups *via* anhydro-aldose tosylhydrazones starting from glycosyl cyanides.^34,35^

**Scheme 1:**
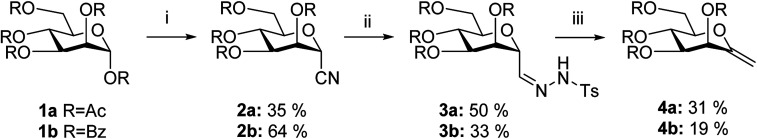
(i) 3.2 equiv. TMSCN, 2 equiv. BF_3_·OEt_2_ in CH_3_NO_2_, 40 °C; (ii) 1.3 equiv. TsNHNH_2_, 8.4 equiv. NaH_2_PO_2_, RANEY®-Ni in pyridine–AcOH–H_2_O; (iii) 5 equiv. K_3_PO_4_ in dry dioxane, reflux.

By using the latter method, *exo*-mannals 4a and 4b were synthesized in multistep reactions from commercially available 1,2,3,4,6-penta-*O*-acetyl- (1a) and -benzoyl-d-mannopyranose (1b), as summarized in Scheme 1. First, *O*-peracylated mannopyranoses 1a or 1b were reacted^36^ with TMSCN in the presence of BF_3_·OEt_2_ to give mannosyl cyanides 2a^37^ and 2b in 35 and 64% yields, respectively, as single anomers. Their NMR study clearly showed the ^4^*C*_1_ conformation of the ring and the α(d) anomeric configuration which can be explained by the anchimeric effect of the 2-*O*-acyl substituent and also corresponds to the anomeric effect of the CN substituent forcing this group to occupy an axial position.^38–40^

Subsequently cyanides 2 were transformed into the tosylhydrazones 3 under reductive conditions in the presence of tosylhydrazine, which on deprotonation by K_3_PO_4_ ^41^ (instead of the less easily handled NaH^34^) and heating to reflux temperature gave *exo*-mannals 4. The pyranoid ring of tosylhydrazones 3 and *exo*-mannals 4 had a ^5^*C*_2_ conformation according to the ^3^*J* coupling constants between H-2, H-3, H-4, H-5 and H-6 (Table 1).

**Table 1:** Selected NMR data (*δ* [ppm], ^3^*J*_H,H_ [Hz]) of tosylhydrazones 3 and *exo*-mannals 4

		H-2	H-3	H-4	H-5	H-6
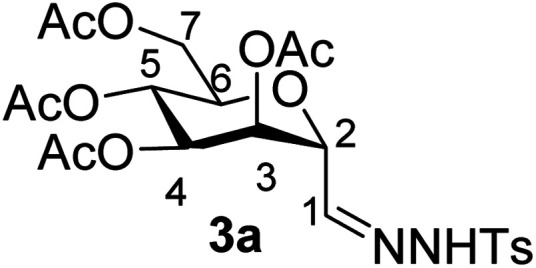	*δ*	4.56	5.57	5.30	5.23	3.66
	*J*	*3.2*, *2.6*	*3.5*, *2.6*	9.2, *3.5*	9.2	9.2, *5.3*, 2.6
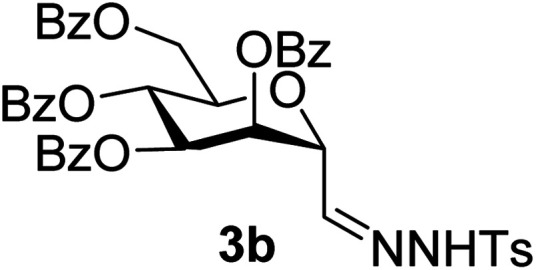	*δ*	4.84	6.04	5.88	6.09	4.12
	*J*	*2.7*, *1.9*	*3.1*, *1.9*	9.8, *3.1*	*9.8*	9.8, *4.1*, *2.2*
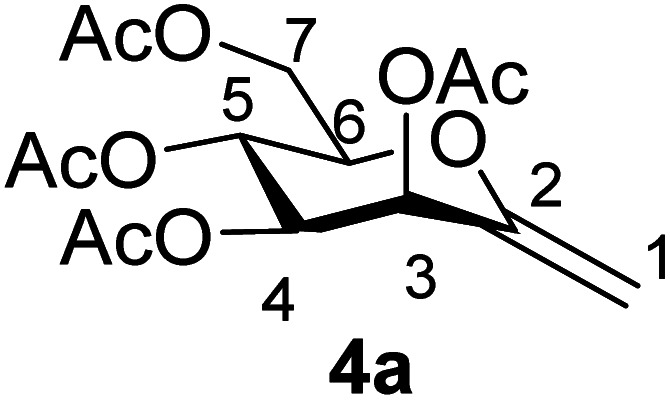	*δ*	—	5.71	5.10	5.43	3.82
	*J*	—	*3.5*	*9.5*, *3.5*	*9.5*	*9.5*, *5.2*, *2.6*
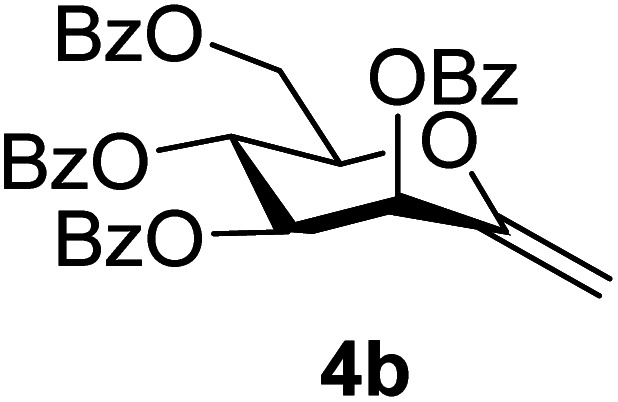	*δ*	—	6.17	5.68	6.28	4.29
	*J*	—	*3.4*	*9.7*, *3.4*	*9.7*	*9.7*, *4.1*, *2.7*

The benzylated pyranoid *exo*-mannal 5 ^31^ and the isopropylenated furanoid *exo*-mannal 6 ^32,42^ were synthesized by literature procedures.

### 2.2. Thiol–ene additions of *exo*-mannal derivatives

The addition of thiols was carried out in toluene at room temperature (unless otherwise indicated) in the presence of 2,2-dimethoxy-2-phenylacetophenone (DPAP, 0.1 equiv.) as the photoinitiator with irradiation at *λ*_max_ 365 nm for 15 min. The progress of the reaction was monitored by TLC after this reaction period and irradiation and addition of DPAP were repeated if necessary (in most cases two irradiation cycles had to be applied for total consumption of *exo*-mannals 4–6). The thiols were used in a 5-fold excess for 7a and 7b and in slightly more than equimolar amounts (1.1 equiv.) for 7c–f.

The results of the addition of thiols 7 to *exo*-mannals 4 and 5 are summarized in Table 2. The reactions were carried out under an Ar atmosphere (except in the case of thiol 7d) to give the expected products 8 in high yields. On addition of thiol 7d to both *exo*-mannals 4a and 4b under air, beside the glycosylmethyl sulfides 8ad and 8bd, respectively, as the major products, sulfoxides 9ad and 9bd were also isolated as minor components. The structure of these side products was identified by NMR and MS measurements, and their formation could be eliminated by using an inert atmosphere.

**Table 2:** Addition of thiols 7 to *exo*-mannals 4 and 5^a^

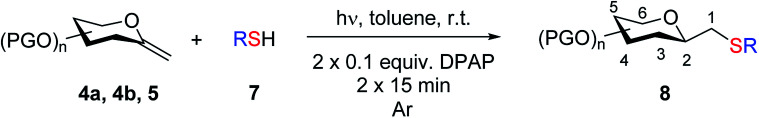			
*Exo*-Glycal	Thiols	Yield^b^ (%) of 8	Structure of adducts 8
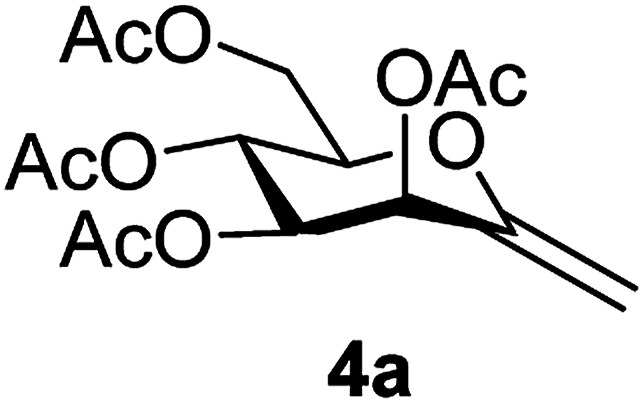	7a	79^c^	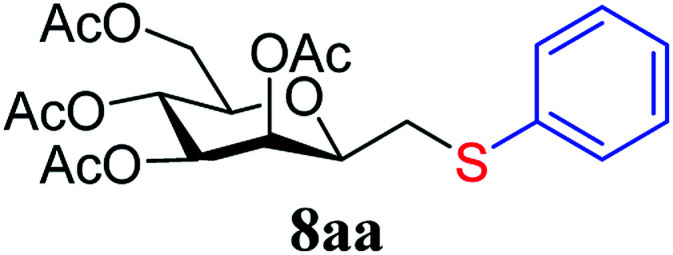
	7b	69	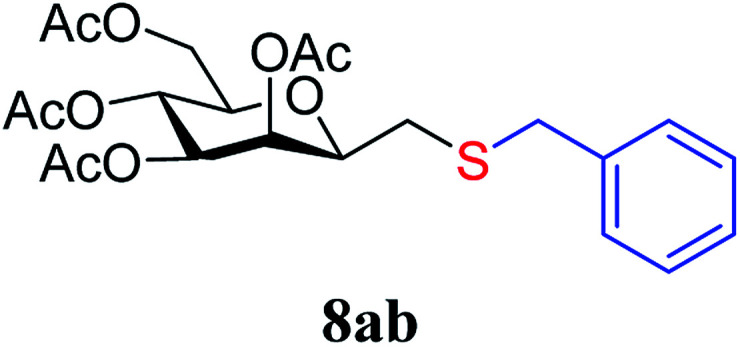
	7c	78	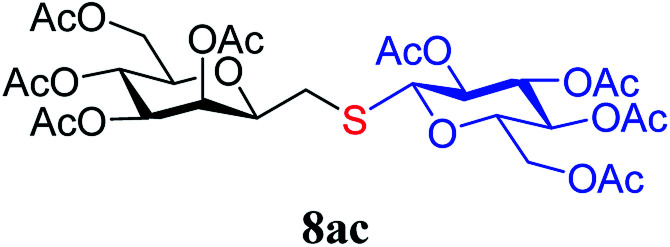
	7d	71^d^	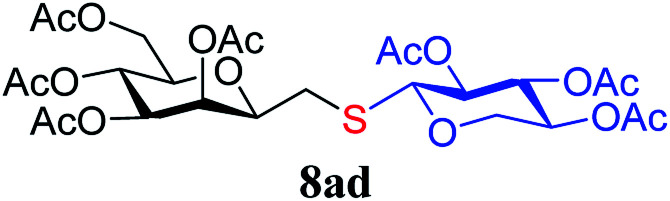
	7e	79	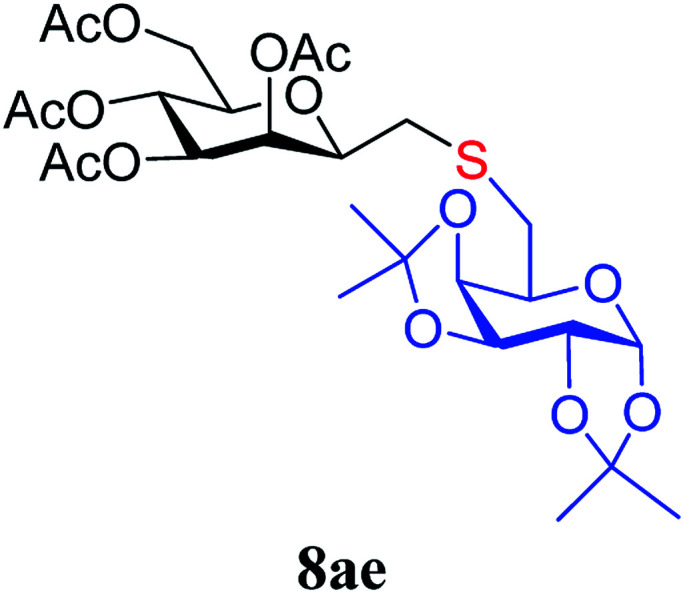
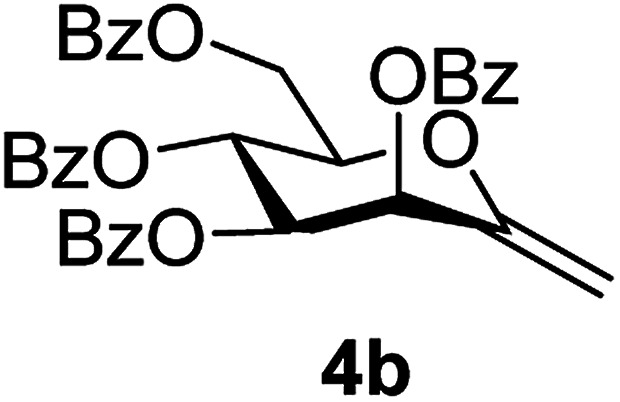	7a	47^c^	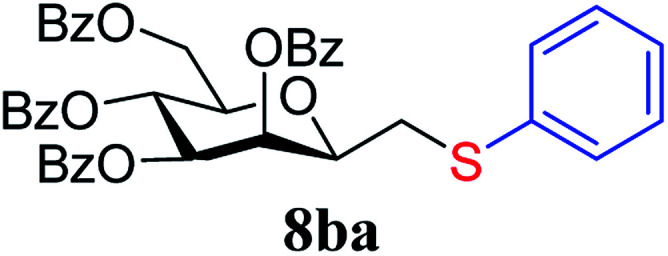
	7b	51	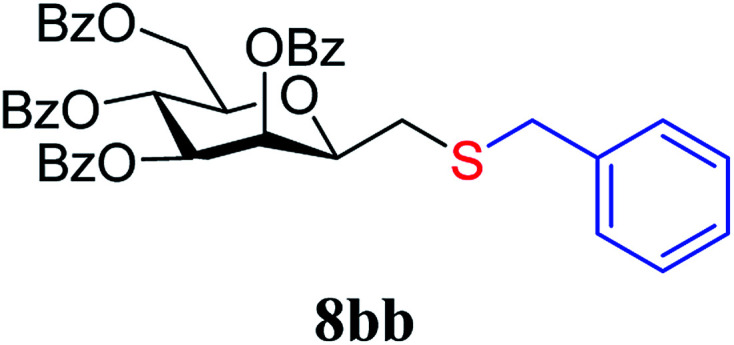
	7d	68^d^	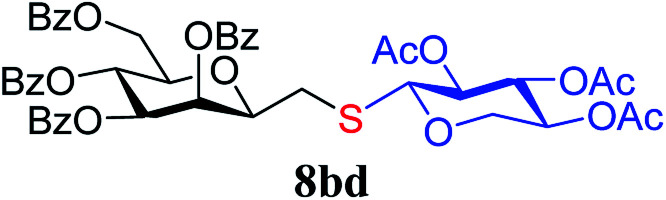
	7e	73	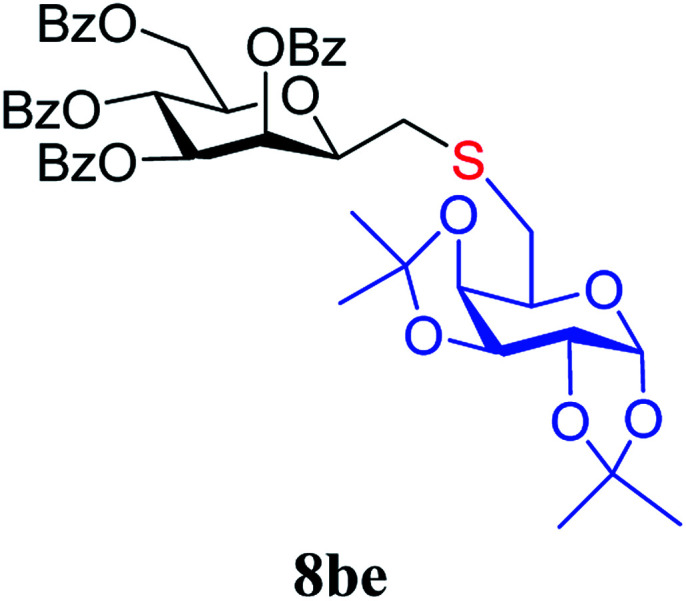
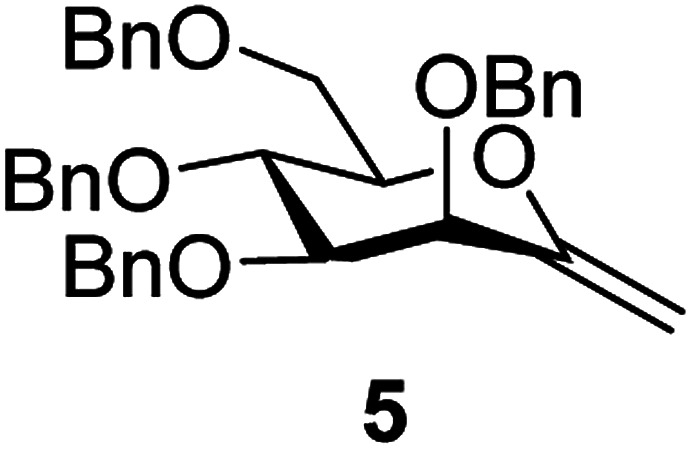		Low conversion and decomposition of *exo*-mannal 5 were observed with 7a, b, d	

a Total conversion of 4a, b was detected after two irradiations of 15 min.

b Isolated yields after purification by column chromatography.

c The reaction was performed at −78 °C. At room temperature, no conversion of the *exo*-glycal was detected.

d The reaction was performed under air. Formation of corresponding sulfoxide 9 in low yield was also observed: 
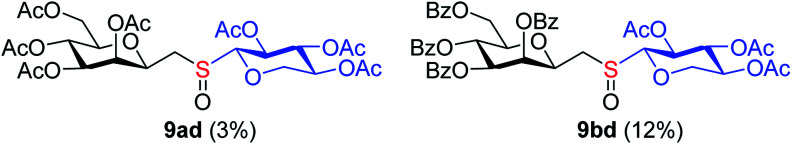

In the case of benzenethiol 7a no transformation was detected at room temperature, but at −78 °C ^43,44^ the sulfides 8aa and 8ba were isolated by column chromatography in 79 and 47% yields, respectively.

There are only a few examples in the literature for thiol–ene addition to benzylated carbohydrate derivatives, due to the low stability of this protecting group under radical conditions.^23,45^ This experience was corroborated with *exo*-mannal 5, whose reactions proceeded only with low conversion and decomposition of the starting benzylated glycal (Table 2).

The addition of thiols 7 to the furanoid *exo*-mannal 6 was carried out under the same conditions as described above (under argon atmosphere and at room temperature), and the results are summarized in Table 3. After the second irradiation total conversion of starting compound was observed, and the desired glycosylmethyl sulfides 10 were isolated in moderate to good yields (53–82%). In the case of benzenethiol 7a the reaction at room temperature gave 10a in 53% yield, while at low temperature the yield raised to 70%.

**Table 3:** Addition of thiols 7 to *exo*-mannals 6^a^

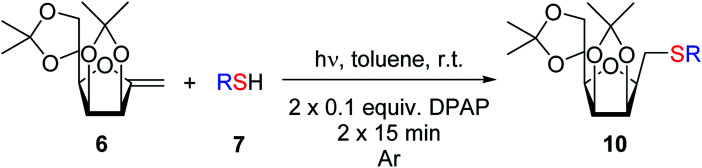		
Thiols	Yield^b^ (%) of 10	Structure of adducts 10
7a	53 (70^c^)	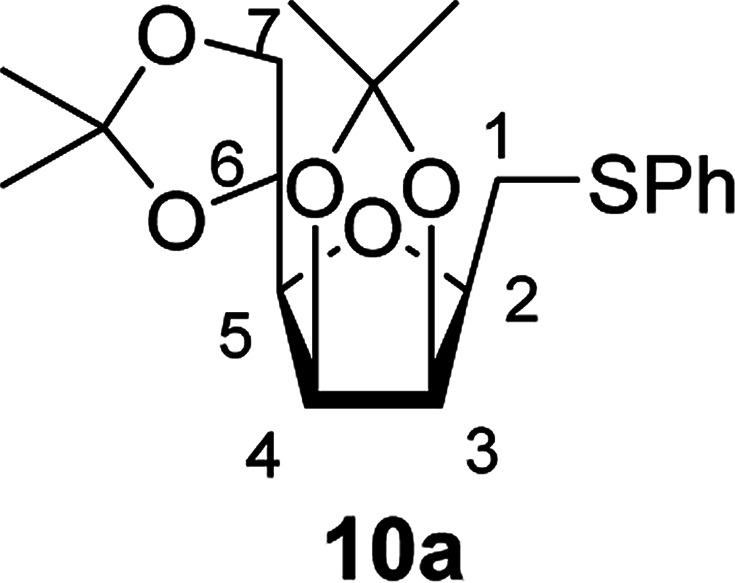
7c	82	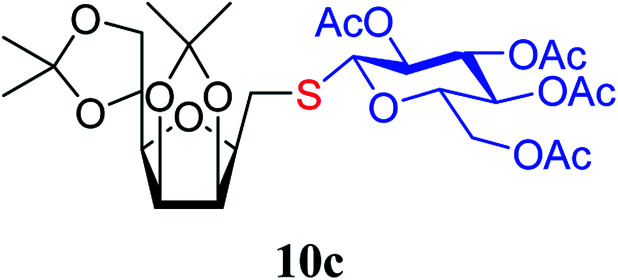
7e	70	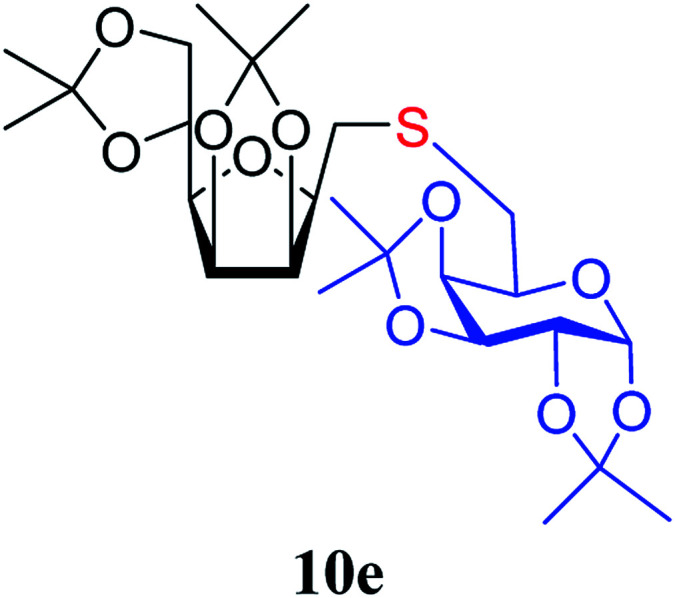
7f	74	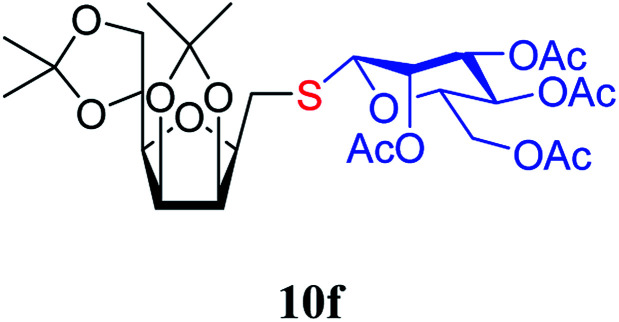

a Total conversion of 6 was detected after two irradiations of 15 min.

b Isolated yields after purification by column chromatography.

c The reaction was performed at −78 °C.

The structure of the products 8 and 10 was identified by assigning each signal and connectivity in their ^1^H NMR spectra by using COSY experiments (selected data are collected in Table 4).

**Table 4:** ^1^H-NMR data (*δ* [ppm], ^3^*J*_H,H_ [Hz]) of selected thiol adducts^a^

		H-1_A_	H-1_B_	H-2	H-3	H-4	H-5^b^	H-6
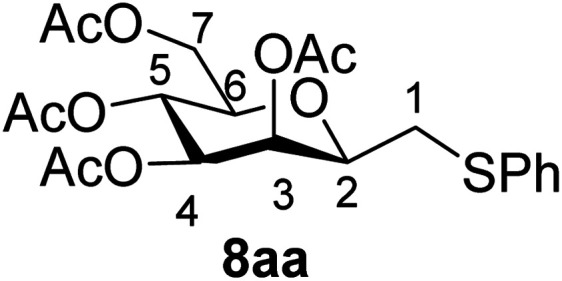	*δ*	3.16	2.92	3.68	5.55	5.02	5.19	3.61
	*J*	*13.9*, *6.8*	*13.9*, *7.0*	*7.0*, *6.8*, *1.0*	*3.4*, *1.0*	*10.0*, *3.4*	*10.0*	*10.0*, *5.6*, *2.3*
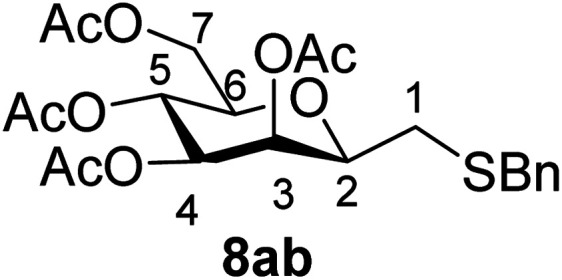	*δ*	2.64	2.40	3.60–3.56	5.41	4.97	5.19	3.60–3.56
	*J*	*14.0*, *7.3*	*14.0*, *6.2*	*m*	*3.2*	*10.0*, *3.4*	*10.0*	*m*
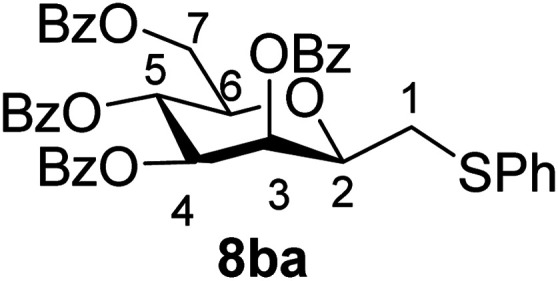	*δ*	3.25	3.05	4.01	6.03	5.59	6.02	4.08
	*J*	*14.1*, *7.1*	*14.1*, *6.5*	*7.1*, *6.5*, *0.9*	*3.2*, *0.9*	*10.0*, *3.2*	*10.0*	*10.0*, *4.6*, *2.7*
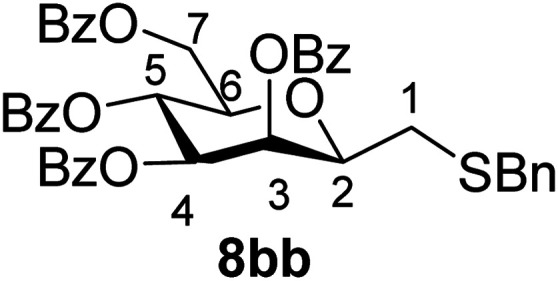	*δ*	2.73	2.51	3.95	5.89	5.56	6.01	4.07
	*J*	*14.4*, *7.8*	*14.4*, *5.5*	*7.8*, *5.5*, *0.8*	*3.2*, *0.8*	*10.0*, *3.2*	*10.0*	*10.0*, *4.6*, *2.5*
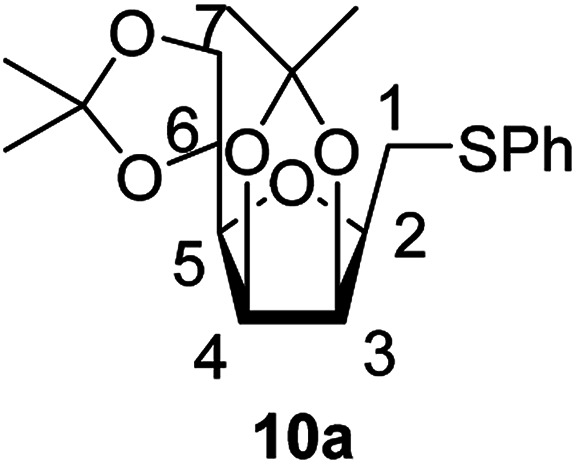	*δ*	3.24	3.22	3.67	4.79–4.73	3.49	4.38	
	*J*	*13.5*, *6.1*	*13.5*, *7.7*	*7.7*, *6.1*, *2.9*	*m*	*7.5*, *2.9*	*7.5*, *6.0*, *4.7*	
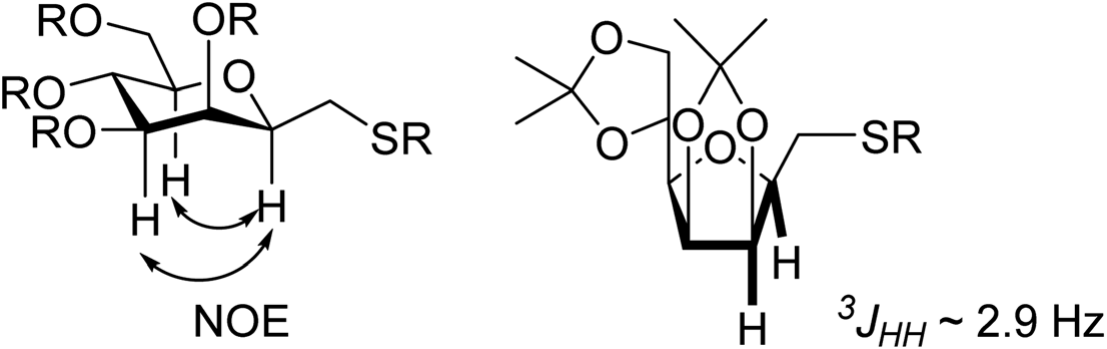								

a The NMR experiments were performed at 400 MHz in CDCl_3_.

b In the case of compounds 8 the signals H-5 were split into triplet.

The vicinal coupling constant between H-2, H-3, H-4, H-5 and H-6 indicated the ^5^*C*_2_ conformation of the sugar ring of 8. The β(d)-configuration at C-2 of 8 could not be determined from the coupling constants between H-2 and H-3 but it was easily assigned from the observed NOE-s between H-2, H-4 and H-6, which also confirm the ^5^*C*_2_ ring conformation of the products. In the case of furanoid derivatives the ^3^*J*_H2,H3_ values of ∼2.9 Hz clearly indicated the β(d)-configuration at C-2 of 10.

**Fig. 1:**
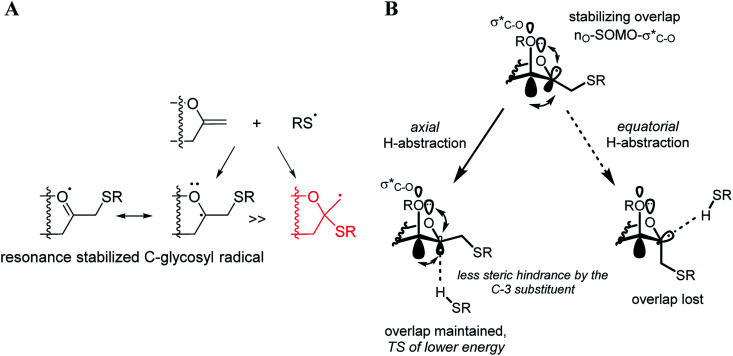
Mechanistic considerations. (A) Relative stabilities of adduct radical; (B) stabilizing overlaps in mannopyranosyl radicals and transition states of H-abstraction.

The exclusive regio- and stereoselectivity of these reactions can be explained by the following mechanistic considerations. The regiochemistry of the additions is determined by the different stability of the radicals that may form upon addition of the thiyl radicals to the exocyclic double bonds. The resonance stabilized *C*-glycosyl radical provides a reaction pathway with a significantly lower activation barrier than the glycosylmethyl radical (Fig. 1A). In the ^5^*C*_2_ conformation of mannopyranosyl radicals^46^ there are stabilizing overlaps between the 
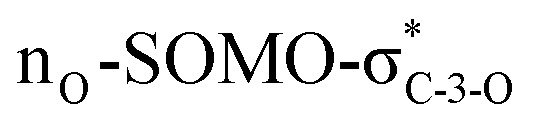
 orbitals due to the homo-anomeric effect. Similar considerations refer to the furanosyl radicals. The abstraction of the hydrogen by these radicals are clearly more favourable from the α-side, since both the steric shielding by the *O*-3-substituent and the preservation of the above stabilizing overlaps act in synergy (Fig. 1B). These effects lead to a more favorable transition state (TS) with lower energy, thus determining the exclusively observed β configuration of C-2.

## 3. Conclusion

A synthesis of *O*-peracylated *exo*-mannals was developed from mannopyranosyl cyanides and the respective anhydro-aldose tosylhydrazones. Photoinitianed thiol–ene additions of these *O*-peracylated and also *O*-peracetalated *exo*-mannals of both pyranoid and furanoid structures were studied to result in *C*-mannosylmethyl sulfide type compounds with the R-S-CH_2_ appendage in (pseudo)equatorial position as the only products. This study demonstrated that the radical-mediated thiol–ene reactions of *exo*-mannals with a wide range of thiols took place with exclusive regio- and stereoselectivities, thereby providing a new way for the construction of novel types of glycomimetic compounds of high biological relevance.

## 4. Materials and methods

### 4.1. General methods

Melting points were measured on a Kofler hot-stage and are uncorrected. Optical rotations were measured at r.t. with a Jasco P-2000 polarimeter. TLC was performed on Kieselgel 60 F_254_ (Merck) with detection by immersing into 5% ethanolic sulfuric acid soln. followed by heating. Column chromatography was performed on Silica gel 60 (Merck 0.063–0.200 mm). Organic solutions were dried over MgSO_4_ and concentrated under diminished pressure. The ^1^H (400 MHz) and ^13^C NMR (100.28 MHz) NMR spectra were recorded with a Bruker DRX-400 spectrometer. Chemical shifts are referenced to Me_4_Si (0.00 ppm for ^1^H) and to the residual solvent signals (CDCl_3_: 77.16 ppm for ^13^C). The coupling constant values (*J*) are given in Hz. Mass spectra were recorded with MicroTOF-Q type Qq-TOF MS (Bruker Daltonik, Bremen, Germany) instruments. The photoinitiated reactions were carried out by irradiation with a Hg-lamp (maximum emission at 365 nm) in a borosilicate vessel. The benzylated pyranoid *exo*-mannal (5) and isopropylenated furanoid *exo*-mannal (6) were synthesized by literature procedures.^31,32,42^

#### 4.1.1. Method A: general procedure for the preparation of *O*-peracylated d-mannopyranosyl cyanides

To a stirred solution of an *O*-peracylated mannopyranose (1 mmol each) in CH_3_NO_2_ (5 mL) TMSCN (0.4 mL; 3.2 mmol) and BF_3_·OEt_2_ (0.25 mL; 2 mmol) were added. The mixture was stirred at 40 °C and the progress of the reaction was controlled by TLC (eluent: hexane–EtOAc = 2 : 1). When the starting material disappeared, the solvent was evaporated, and the residue was dissolved in Et_2_O (50 mL). This was washed with saturated NaHCO_3_ solution (2 × 20 mL) and brine (1 × 20 mL), dried, then concentrated and purified by column chromatography.

##### 4.1.1.1. 2,3,4,6-Tetra-*O*-acetyl-α-d-mannopyranosyl cyanide (2a)

Prepared from 1a (5 g, 12.8 mmol) according to Method A to give 2a by column chromatography (eluent: hexane–acetone = 3 : 1) as a yellow syrup (1.6 g, 35%). *R*_f_ = 0.39 (hexane–EtOAc = 1 : 1). [*α*]_D_ = +11.4 (*c* = 0.244, CHCl_3_); lit^37^ +27.8 (*c* 3.32, CHCl_3_).

^1^H-NMR of 2a (400 MHz, CDCl_3_) *δ*: 5.44 (dd, 1H, *J* = 3.1, 2.3 Hz, H-2), 5.38–5.28 (m, 2H, H-3, H-4), 4.92 (d, 1H, *J* = 2.3 Hz, H-1), 4.34 (dd, 1H, *J* = 12.6, 5.3 Hz, H-6_A_), 4.17 (dd, 1H, *J* = 12.6, 2.0 Hz, H-6_B_), 4.08 (ddd, 1H, *J* = 9.4, 5.3, 2.0 Hz, H-5), 2.19, 2.11, 2.08, 2.03 (4 × s, 4 × 3H, OAc); data correspond to lit^37^ values. ^13^C-NMR of 2a (100 MHz, CDCl_3_) *δ*: 170.5, 169.7, 169.6, 169.6 (CO), 113.5 (CN), 74.3, 69.0, 68.8, 65.7, 65.1 (C-1, C-2, C-3, C-4, C-5), 61.8 (C-6), 20.8, 20.7, 20.7, 20.6 (OAc). ESI-MS positive mode (*m*/*z*): calcd for C_15_H_19_NNaO_9_^+^ [M + Na]^+^ = 380.0952. Found: [M + Na]^+^ = 380.0970.

##### 4.1.1.2. 2,3,4,6-Tetra-*O*-benzoyl-α-d-mannopyranosyl cyanide (2b)

Prepared from 1b (5 g, 7.1 mmol) according to Method A to give 2b by column chromatography (eluent: hexane–acetone = 4 : 1) as a yellow syrup (2.2 g, 53%). *R*_f_ = 0.31 (hexane–EtOAc = 2 : 1). [*α*]_D_ = −32.9 (*c* = 0.254, CHCl_3_).

^1^H-NMR of 2b (400 MHz, CDCl_3_) *δ*: 8.10 (dd, 2H, *J* = 8.1, 1.3 Hz, aromatic), 8.02 (dd, 2H, *J* = 8.2, 1.1 Hz, aromatic), 7.98 (dd, 2H, *J* = 8.2, 1.0 Hz, aromatic), 7.85 (dd, 2H, *J* = 8.3, 1.1 Hz, aromatic), 7.63–7.26 (m, 12H, aromatic), 6.18 (pseudo t, 1H, *J* = 9.8 Hz, H-4), 5.94–5.89 (m, 2H, H-2, H-3), 5.21 (d, 1H, *J* = 2.0 Hz, H-1), 4.77 (dd, 1H, *J* = 10.6, 3.2 Hz, H-6_A_), 4.56–4.51 (m, 2H, H-5, H-6_B_). ^13^C-NMR of 2b (100 MHz, CDCl_3_) *δ*: 165.4, 165.2 (CO), 134.1–128.6 (aromatic), 113.8 (CN), 74.6, 70.1, 69.8, 65.9, 65.7 (C-1, C-2, C-3, C-4, C-5), 62.1 (C-6). ESI-MS positive mode (*m*/*z*): calcd for C_35_H_27_NNaO_9_^+^ [M + Na]^+^ = 628.1578. Found: [M + Na]^+^ = 628.1573.

#### 4.1.2. Method B: general procedure for the preparation of anhydro-aldose tosylhydrazones

RANEY®-Ni (1.5 g) was added to a vigorously stirred mixture of pyridine (5.7 mL), AcOH (3.4 mL), and H_2_O (3.4 mL) at room temperature. Subsequently, NaH_2_PO_2_ (0.74 g, 8.4 mmol), *p*-toluenesulfonyl hydrazide (0.22 g, 1.2 mmol), and the corresponding mannosyl cyanide (2a–b, 1 mmol each) were added to the mixture. When the reaction was complete (TLC, eluent: hexane–EtOAc = 2 : 1) the insoluble materials were filtered off with suction and washed with CH_2_Cl_2_ (10 mL). The organic layer of the filtrate was separated, washed sequentially with H_2_O (5 mL), 10% aqueous solution of HCl (2 × 5 mL), cold, saturated NaHCO_3_ solution (2 × 5 mL) and H_2_O (5 mL), and then dried over anhydrous MgSO_4_. The solution was concentrated under reduced pressure, and traces of pyridine were removed by repeated co-evaporations of toluene. The residue was purified by column chromatography.

##### 4.1.2.1. 3,4,5,7-Tetra-*O*-acetyl-2,6-anhydro-d-*glycero*-d-*talo*-heptose tosylhydrazone (3a)

Prepared from 2a (1.6 g, 4.5 mmol) according to Method B to give 3a by column chromatography (eluent: hexane–EtOAc = 2 : 1) as a yellow amorphous solid (1.2 g, 50%). *R*_f_ = 0.36 (hexane–EtOAc = 1 : 1). [*α*]_D_ = −37.7 (*c* = 0.200, CHCl_3_).

^1^H-NMR of 3a (400 MHz, CDCl_3_) *δ*: 8.63 (s, 1H, NH), 7.92 (d, 2H, *J* = 8.4 Hz, aromatic), 7.34 (d, 2H, *J* = 8.4 Hz, aromatic), 7.17 (d, 1H, *J* = 3.2 Hz, H-1), 5.57 (dd, 1H, *J* = 3.5, 2.6 Hz, H-3), 5.30 (dd, 1H, *J* = 9.2, 3.5 Hz, H-4), 5.23 (pseudo t, 1H, *J* = 9.2 Hz, H-5), 4.56 (dd, 1H, *J* = 3.2, 2.6 Hz, H-2), 4.24 (dd, 1H, *J* = 12.3, 5.3 Hz, H-7_A_), 4.05 (dd, 1H, *J* = 12.3, 2.6 Hz, H-7_B_), 3.66 (ddd, 1H, *J* = 9.2, 5.3, 2.6 Hz, H-6), 2.42 (CH_3_), 2.12, 2.09, 2.05, 2.03 (4 × s, 4 × 3H, OAc). ^13^C-NMR of 3a (100 MHz, CDCl_3_) *δ*: 170.9, 170.3, 169.8, 169.8 (CO), 144.1 (C-1), 144.6–128.2 (aromatic), 74.7, 72.1, 69.3, 68.2, 66.5 (C-2, C-3, C-4, C-5, C-6), 62.3 (C-7), 21.7 (CH_3_), 20.9, 20.8, 20.8, 20.7 (OAc). ESI-MS positive mode (*m*/*z*): calcd for C_22_H_28_N_2_NaO_11_S^+^ [M + Na]^+^ = 551.1306. Found: [M + Na]^+^ = 551.1316.

##### 4.1.2.2. 3,4,5,7-Tetra-*O*-benzoyl-2,6-anhydro-d-*glycero*-d-*talo*-heptose tosylhydrazone (3b)

Prepared from 2b (1.7 g, 2.8 mmol) according to Method B to give 3b by column chromatography (eluent: hexane–EtOAc = 2 : 1) as a yellow amorphous solid (0.7 g, 33%). *R*_f_ = 0.44 (hexane–EtOAc = 1 : 1). [*α*]_D_ = +3.7 (*c* = 0.198, CHCl_3_).

^1^H-NMR of 3b (400 MHz, CDCl_3_) *δ*: 8.90 (s, 1H, NH), 8.10–7.26 (m, 25H, aromatic, H-1), 6.09 (pseudo t, 1H, *J* = 9.8 Hz, H-5), 6.04 (dd, 1H, *J* = 3.1, 1.9 Hz, H-3), 5.88 (dd, 1H, *J* = 9.8, 3.1 Hz, H-4), 4.84 (dd, 1H, *J* = 2.7, 1.9 Hz, H-2), 4.65 (dd, 1H, *J* = 12.2, 2.2 Hz, H-7_A_), 4.40 (dd, 1H, *J* = 12.2, 4.1 Hz, H-7_B_), 4.12 (ddd, 1H, *J* = 9.8, 4.1, 2.2 Hz, H-6), 2.41 (CH_3_). ^13^C-NMR of 3b (100 MHz, CDCl_3_) *δ*: 166.3, 165.6, 165.4 (CO), 144.3 (C-1), 144.8–128.5 (aromatic), 75.0, 72.4, 70.5, 69.5, 67.3 (C-2, C-3, C-4, C-5, C-6), 62.8 (C-7), 21.7 (CH_3_). ESI-MS positive mode (*m*/*z*): calcd for C_42_H_36_N_2_NaO_11_S^+^ [M + Na]^+^ = 799.1932. Found: [M + Na]^+^ = 799.1923.

#### 4.1.3. Method C: general procedure for the synthesis of *O*-peracylated *exo*-glycals

To the stirred and refluxed suspension of K_3_PO_4_ (0.1 g, 5 mmol) in dry 1,4-dioxane (12 mL), a solution of an anhydro-aldose tosylhydrazone (3a–b, 1 mmol each) in dry 1,4-dioxane (12 mL) was added dropwise. When the reaction was complete (TLC, eluent: hexane–EtOAc = 1 : 1), the mixture was cooled and the insoluble material filtered off. The solvent was removed under reduced pressure, and the residue was purified by column chromatography.

##### 4.1.3.1. 3,4,5,7-Tetra-*O*-acetyl-2,6-anhydro-1-deoxy-d-*manno*-hept-1-enitol (4a)

Prepared from 3a (1.0 g, 1.9 mmol) according to Method C to give 4a by column chromatography (eluent: hexane–EtOAc = 2 : 1) as a yellow oil (0.2 g, 31%). *R*_f_ = 0.41 (hexane–EtOAc = 1 : 1). [*α*]_D_ = +34.7 (*c* = 0.126, CHCl_3_).

^1^H-NMR of 4a (400 MHz, CDCl_3_) *δ*: 5.71 (d, 1H, *J* = 3.5 Hz, H-3), 5.43 (pseudo t, 1H, *J* = 9.5 Hz, H-5), 5.10 (dd, 1H, *J* = 9.5, 3.5 Hz, H-4), 4.88 (d, 1H, *J* = 1.4 Hz, H-1_A_), 4.73 (d, 1H, *J* = 1.4 Hz, H-1_B_), 4.31 (dd, 1H, *J* = 12.4, 5.2 Hz, H-7_A_), 4.21 (dd, 1H, *J* = 12.4, 2.6 Hz, H-7_B_), 3.82 (ddd, 1H, *J* = 9.5, 5.2, 2.6 Hz, H-6), 2.14, 2.12, 2.07, 2.03 (4 × s, 4 × 3H, OAc). ^13^C-NMR of 4a (100 MHz, CDCl_3_) *δ*: 170.7, 170.0, 169.9, 169.6 (CO), 152.5 (C-2), 102.0 (C-1), 77.1, 71.1, 69.0, 65.6 (C-3, C-4, C-5, C-6), 62.4 (C-7), 21.1, 20.8, 20.7, 20.7 (OAc). ESI-MS positive mode (*m*/*z*): calcd for C_15_H_20_NaO_9_^+^ [M + Na]^+^ = 367.1000. Found: [M + Na]^+^ = 367.0996.

##### 4.1.3.2. 2,6-Anhydro-3,4,5,7-tetra-*O*-benzoyl-1-deoxy-d-*manno*-hept-1-enitol (4b)

Prepared from 3b (0.2 g, 0.26 mmol) according to Method C to give 4b by column chromatography (eluent: hexane–EtOAc = 2 : 1) as a yellow oil (64 mg, 42%). *R*_f_ = 0.43 (hexane–EtOAc = 2 : 1). [*α*]_D_ = +19.0 (*c* = 0.260, CHCl_3_).

^1^H-NMR of 4b (400 MHz, CDCl_3_) *δ*: 8.13–7.85 (m, 8H, aromatic), 7.60–7.26 (m, 13H, aromatic, H-1), 6.28 (pseudo t, 1H, *J* = 9.7 Hz, H-5), 6.17 (d, 1H, *J* = 3.4 Hz, H-3), 5.68 (dd, 1H, *J* = 9.7, 3.4 Hz, H-4), 5.03 (d, 1H, *J* = 1.3 Hz, H-1_A_), 4.94 (d, 1H, *J* = 1.3 Hz, H-1_B_), 4.78 (dd, 1H, *J* = 12.3, 2.7 Hz, H-7_A_), 4.55 (dd, 1H, *J* = 12.3, 4.1 Hz, H-7_B_), 4.29 (ddd, 1H, *J* = 9.7, 4.1, 2.7 Hz, H-6). ^13^C-NMR of 4b (100 MHz, CDCl_3_) *δ*: 165.7, 165.6, 165.4, 165.4 (CO), 152.6 (C-2), 133.7–128.5 (aromatic), 102.8 (C-1), 77.3, 72.2, 70.1, 66.4 (C-3, C-4, C-5, C-6), 62.8 (C-7). ESI-MS positive mode (*m*/*z*): calcd for C_35_H_28_NaO_9_^+^ [M + Na]^+^ = 615.1626. Found: [M + Na]^+^ = 615.1626.

#### 4.1.4. Method D: general procedure for the thiol–ene additions

To a solution of an *exo*-glycal (4a, b, 5, 6 0.5 mmol each) in dry toluene (5 mL), a thiol (2.5 mmol of 7a, b and 0.55 mmol of 7c–f) and 2,2-dimethoxy-2-phenylacetophenone (DPAP, 0.1 mmol) were added. The solution was irradiated by a mercury vapor lamp (*λ*_max_ 365 nm) at room temperature for 15 min. If TLC (eluent: hexane–acetone = 2 : 1) indicated incomplete transformation of the starting material another 0.1 equiv. of DPAP was added and irradiation was continued for 15 min. When the reaction was complete, the solvent was removed, then the residue was purified by column chromatography.

##### 4.1.4.1. 3,4,5,7-Tetra-*O*-acetyl-2,6-anhydro-1-*S*-phenyl-1-thio-d-*glycero*-d-*galacto*-heptitol (8aa)

Prepared from 4a (35 mg, 0.11 mmol) and 7a (56 μL, 0.55 mmol) using DPPA (3 × 2.8 mg, 0.011 mmol) in toluene (1.1 mL) at −78 °C according to Method D to give 8aa by column chromatography (eluent: hexane–acetone = 10 : 1) as a yellow amorphous solid (31 mg, 62%). *R*_f_ = 0.41 (hexane–EtOAc = 2 : 1). [*α*]_D_ = +91.9 (*c* = 0.102, CHCl_3_).

^1^H-NMR of 8aa (400 MHz, CDCl_3_) *δ*: 7.39–7.22 (m, 5H, aromatic), 5.55 (dd, 1H, *J* = 3.4, 1.0 Hz, H-3), 5.23 (pseudo t, 1H, *J* = 10.0 Hz, H-5), 5.02 (dd, 1H, *J* = 10.0, 3.4 Hz, H-4), 4.27 (dd, 1H, *J* = 12.3, 5.6 Hz, H-7_A_), 4.09 (dd, 1H, *J* = 12.3, 2.3 Hz, H-7_B_), 3.68 (ddd, 1H, *J* = 7.0, 6.8, 1.0 Hz, H-2), 3.61 (ddd, 1H, *J* = 10.0, 5.6, 2.3 Hz, H-6), 3.16 (dd, 1H, *J* = 13.9, 6.8 Hz, H-1_A_), 2.92 (dd, 1H, *J* = 13.9, 7.0 Hz, H-1_B_), 2.14, 2.10, 2.03, 1.98 (4 × s, 4 × 3H, OAc). ^13^C-NMR of 8aa (100 MHz, CDCl_3_) *δ*: 170.9, 170.3, 170.3, 169.8 (CO), 135.0–127.1 (aromatic), 76.5, 76.1, 72.4, 68.6, 66.1 (C-2, C-3, C-4, C-5, C-6), 62.8 (C-7), 34.3 (C-1), 20.9, 20.8, 20.7 (OAc). ESI-MS positive mode (*m*/*z*): calcd for C_21_H_26_NaO_9_S^+^ [M + Na]^+^ = 477.1190. Found: [M + Na]^+^ = 477.1189.

##### 4.1.4.2. 3,4,5,7-Tetra-*O*-acetyl-2,6-anhydro-1-*S*-benzyl-1-thio-d-*glycero*-d-*galacto*-heptitol (8ab)

Prepared from 4a (100 mg, 0.29 mmol) and 7b (170 μL, 1.45 mmol) using DPPA (3 × 7.4 mg, 0.029 mmol) in toluene (2.9 mL) according to Method D to give 8ab by column chromatography (eluent: hexane–acetone = 10 : 1) as a yellow amorphous solid (94 mg, 69%). *R*_f_ = 0.26 (hexane–EtOAc = 2 : 1). [*α*]_D_ = +60.5 (*c* = 0.152, CHCl_3_).

^1^H-NMR of 8ab (400 MHz, CDCl_3_) *δ*: 7.32–7.30 (m, 5H, aromatic), 5.41 (d, 1H, *J* = 3.2 Hz, H-3), 5.19 (pseudo t, 1H, *J* = 10.0 Hz, H-5), 4.97 (dd, 1H, *J* = 10.0, 3.4 Hz, H-4), 4.24 (dd, 1H, *J* = 12.3, 5.8 Hz, H-7_A_), 4.12 (dd, 1H, *J* = 12.3, 2.3 Hz, H-7_B_), 3.79 (d, 1H, *J* = 13.4 Hz, CH_2_Ph), 3.72 (d, 1H, *J* = 13.4 Hz, CH_2_Ph), 3.60–3.56 (m, 1H, H-2, H-6), 2.64 (dd, 1H, *J* = 14.0, 7.3 Hz, H-1_A_), 2.40 (dd, 1H, *J* = 14.0, 6.2 Hz, H-1_B_), 2.09, 2.09, 2.04, 1.97 (4 × s, 4 × 3H, OAc). ^13^C-NMR of 8ab (100 MHz, CDCl_3_) *δ*: 170.7, 170.3, 170.2, 169.8 (CO), 138.1–127.3 (aromatic), 77.7, 76.4, 72.3, 68.9, 66.1 (C-2, C-3, C-4, C-5, C-6), 62.9 (C-7), 37.1 (CH_2_Ph), 31.0 (C-1), 20.9, 20.8, 20.7, 20.6 (OAc). ESI-MS positive mode (*m*/*z*): calcd for C_22_H_28_NaO_9_S^+^ [M + Na]^+^ = 491.1346. Found: [M + Na]^+^ = 491.1346.

##### 4.1.4.3. 3,4,5,7-Tetra-*O*-acetyl-2,6-anhydro-1-*S*-(2,3,4,6-tetra-*O*-acetyl-β-d-glucopyranosyl)-1-thio-d-*glycero*-d-*galacto*-heptitol (8ac)

Prepared from 4a (61 mg, 0.18 mmol) and 7c (72 mg, 0.20 mmol) using DPPA (3 × 4.6 mg, 0.018 mmol) in toluene (1.8 mL) according to Method D to give 8ac by column chromatography (eluent: hexane–acetone = 5 : 1) as a yellow amorphous solid (82 mg, 50%). *R*_f_ = 0.32 (hexane–EtOAc = 2 : 1). [*α*]_D_ = −25.10 (*c* = 0.1195, CHCl_3_).

^1^H-NMR of 8ac (400 MHz, CDCl_3_) *δ*: 5.51 (dd, 1H, *J* = 3.4, 1.0 Hz, H-3), 5.26–5.18 (m, 2H, H-5, H-3′), 5.12–5.05 (m, 2H, H-4, H-4′), 5.00 (pseudo t, 1H, *J* = 9.9 Hz, H-2′), 4.49 (d, 1H, *J* = 10.1 Hz, H-1′), 4.30–4.19 (m, 3H, H-7_A_, 
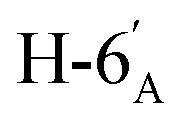
, 
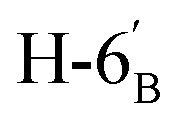
), 4.12 (dd, 1H, *J* = 12.3, 2.4 Hz, H-7_B_), 3.87 (ddd, 1H, *J* = 7.2, 6.8, 1.0 Hz, H-2), 3.74–3.67 (m, 2H, H-6, H-5′), 2.87 (dd, 1H, *J* = 13.9, 6.8 Hz, H-1_A_), 2.69 (dd, 1H, *J* = 13.9, 7.2 Hz, H-1_B_), 2.17, 2.12, 2.11, 2.06, 2.04, 2.03, 2.01, 1.98 (8 × s, 8 × 3H, OAc). ^13^C-NMR of 8ac (100 MHz, CDCl_3_) *δ*: 170.8, 170.7, 170.2, 170.1, 169.8, 169.5, 169.4 (CO), 83.3 (C-1′), 76.9, 76.4, 76.2, 73.8, 72.3, 69.9, 68.6, 68.2, 66.1 (C-2, C-3, C-4, C-5, C-6, C-2′, C-3′, C-4′, C-5′), 62.8 (C-7), 61.9 (C-6′), 29.6 (C-1), 20.9, 20.8, 20.7, 20.6 (OAc). Anal. calcd for C_27_H_40_O_14_S (620.21): C 52.24, H 6.5, S 5.16; measured C 53.07, H 6.60, S 5.03.

##### 4.1.4.4. 3,4,5,7-Tetra-*O*-acetyl-2,6-anhydro-1-*S*-(2,3,4-tri-*O*-acetyl-β-d-xylopyranosyl)-1-thio-d-*glycero*-d-*galacto*-heptitol (8ad)

Prepared from 4a (150 mg, 0.44 mmol) and 7d (150 mg, 0.48 mmol) using DPPA (3 × 11.3 mg, 0.044 mmol) in toluene (4.4 mL) according to Method D to give 8ad by column chromatography (eluent: hexane–acetone = 5 : 1) as a yellow amorphous solid (200 mg, 71%). *R*_f_ = 0.23 (hexane–EtOAc = 1 : 1). [*α*]_D_ = −22.4 (*c* = 0.216, CHCl_3_).

^1^H-NMR of 8ad (400 MHz, CDCl_3_) *δ*: 5.50 (dd, 1H, *J* = 3.2, 0.9 Hz, H-3), 5.22 (pseudo t, 1H, *J* = 10.0 Hz, H-5), 5.15 (pseudo t, 1H, *J* = 8.3 Hz, H-3′), 5.04 (dd, 1H, *J* = 10.0, 3.3 Hz, H-4), 4.96–4.91 (m, 2H, H-2′, H-4′), 4.51 (d, 1H, *J* = 8.4 Hz, H-1′), 4.28–4.19 (m, 2H, 
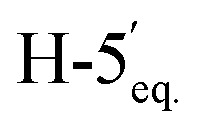
, H-7_A_), 4.12 (dd, 1H, *J* = 12.3, 2.2 Hz, H-7_B_), 3.79 (ddd, 1H, *J* = 7.2, 6.6, 0.9 Hz, H-2), 3.66 (ddd, 1H, *J* = 10.0, 5.4, 2.3 Hz, H-6), 3.41 (dd, 1H, *J* = 11.7, 9.0 Hz, 
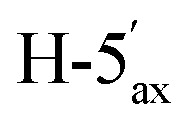
), 2.88 (dd, 1H, *J* = 13.8, 6.6 Hz, H-1_A_), 2.69 (dd, 1H, *J* = 13.8, 7.2 Hz, H-1_B_), 2.18, 2.10, 2.08, 2.05, 2.05, 2.04, 1.98, (7 × s, 7 × 3H, OAc). ^13^C-NMR of 8ad (100 MHz, CDCl_3_) *δ*: 170.8, 170.3, 170.2, 169.9, 169.8, 169.7, 169.6 (CO), 83.4, 77.0, 76.5, 72.4, 71.9, 69.9, 68.5, 66.0, (C-2, C-3, C-4, C-5, C-6, C-1′, C-2′, C-3′, C-4′), 65.5 (C-7), 62.8 (C-5′), 29.6 (C-1), 20.9, 20.8, 20.7 (OAc). ESI-MS positive mode (*m*/*z*): calcd for C_26_H_36_NaO_16_S^+^ [M + Na]^+^ = 659.1616. Found: [M + Na]^+^ = 659.1615.

##### 4.1.4.5. 3,4,5,7-Tetra-*O*-acetyl-2,6-anhydro-1-*S*-(1,2:3,4-di-*O*-isopropylidene-β-d-galactopyranos-6-yl)-1-thio-d-*glycero*-d-*galacto*-heptitol (8ae)

Prepared from 4a (60 mg, 0.17 mmol) and 7e (52 mg, 0.19 mmol) using DPPA (3 × 4.4 mg, 0.017 mmol) in toluene (1.7 mL) according to Method D to give 8ae by column chromatography (eluent: hexane–acetone = 5 : 1) as a yellow amorphous solid (102 mg, 83%). *R*_f_ = 0.28 (hexane–EtOAc = 2 : 1). [*α*]_D_ = −30.92 (*c* = 0.084, CHCl_3_).

^1^H-NMR of 8ae (400 MHz, CDCl_3_) *δ*: 5.54–5.52 (m, 2H, H-3, H-1′), 5.20 (pseudo t, 1H, *J* = 9.9 Hz, H-5), 5.10 (dd, 1H, *J* = 10.0, 3.4 Hz, H-4), 4.61 (dd, 1H, *J* = 7.8, 2.4 Hz, H-3′), 4.31 (dd, 1H, *J* = 5.1, 2.4 Hz, H-2′), 4.26–4.22 (m, 2H, H-7_A_, H-4′), 4.12 (dd, 1H, *J* = 12.3, 2.4 Hz, H-7_B_), 3.90 (dd, 1H, *J* = 7.0, 6.7, 0.8 Hz, H-2), 3.86 (ddd, 1H, *J* = 7.4, 6.2, 1.7 Hz, H-5′), 3.68 (ddd, 1H, *J* = 9.9, 5.9, 2.4 Hz, H-6), 2.82–2.76 (m, 3H, H-1_A_, 
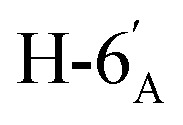
, 
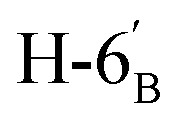
), 2.62 (dd, 1H, *J* = 14.1, 6.7 Hz, H-1_B_), 2.15, 2.10, 2.05, 1.98 (4 × s, 4 × 3H, CH_3_), 1.56, 1.45, 1.34, 1.26, (4 × s, 4 × 3H, OAc). ^13^C-NMR of 8ae (100 MHz, CDCl_3_) *δ*: 170.8, 170.3, 170.1, 169.8 (CO), 109.4, 108.8 (C_acetal_), 96.6 (C-1′), 77.7, 76.4, 72.3, 72.0, 71.0, 70.5, 69.0, 68.3, 66.4 (C-2, C-3, C-4, C-5, C-6, C-2′, C-3′, C-4′, C-5′), 63.0 (C-7), 33.3 (C-1), 29.8 (C-6′), 26.1, 26.0, 25.0, 24.5 (CH_3_), 20.9, 20.8, 20.7 (OAc). Anal. calcd for C_27_H_40_O_14_S (620.21): C 52.24, H 6.50, S 5.16; measured C 52.58, H 6.62, S 5.08.

##### 4.1.4.6. 2,6-Anhydro-3,4,5,7-tetra-*O*-benzoyl-1-*S*-phenyl-1-thio-d-*glycero*-d-*galacto*-heptitol (8ba)

Prepared from 4b (110 mg, 0.19 mmol) and 7a (195 μL, 1.9 mmol) using DPPA (3 × 5.0 mg, 0.019 mmol) in toluene (1.9 mL) at −78 °C according to Method D to give 8ba by column chromatography (eluent: hexane–acetone = 10 : 1) as a yellow amorphous solid (60 mg, 47%). *R*_f_ = 0.33 (hexane–EtOAc = 2 : 1). [*α*]_D_ = +24.3 (*c* = 0.180, CHCl_3_).

^1^H-NMR of 8ba (400 MHz, CDCl_3_) *δ*: 8.12 (dd, 2H, *J* = 8.3, 1.3 Hz, aromatic), 8.02 (dd, 2H, *J* = 8.3, 1.2 Hz, aromatic), 7.89 (dd, 2H, *J* = 8.4, 1.3 Hz, aromatic), 7.79 (dd, 2H, *J* = 8.4, 1.3 Hz, aromatic), 7.63–7.18 (m, 17H, aromatic), 6.03 (dd, 1H, *J* = 3.2, 0.9 Hz, H-3), 6.02 (pseudo t, 1H, *J* = 10.0 Hz, H-5), 5.59 (dd, 1H, *J* = 10.0, 3.2 Hz, H-4), 4.71 (dd, 1H, *J* = 12.2, 2.7 Hz, H-7_A_), 4.46 (dd, 1H, *J* = 12.2, 4.6 Hz, H-7_B_), 4.08 (ddd, 1H, *J* = 10.0, 4.6, 2.7 Hz, H-6), 4.01 (ddd, 1H, *J* = 7.1, 6.5, 0.9 Hz, H-2), 3.25 (dd, 1H, *J* = 14.1, 7.1 Hz, H-1_A_), 3.05 (dd, 1H, *J* = 14.1, 6.5 Hz, H-1_B_). ^13^C-NMR of 8ba (100 MHz, CDCl_3_) *δ*: 166.3, 165.9, 165.6, 165.5 (CO), 133.6–127.0 (aromatic), 76.6, 76.5, 73.4, 69.7, 67.1 (C-2, C-3, C-4, C-5, C-6), 63.3 (C-7), 34.6 (C-1). ESI-MS positive mode (*m*/*z*): calcd for C_41_H_34_NaO_9_S^+^ [M + Na]^+^ = 725.1816. Found: [M + Na]^+^ = 725.1818.

##### 4.1.4.7. 2,6-Anhydro-3,4,5,7-tetra-*O*-benzoyl-1-*S*-benzyl-1-thio-d-*glycero*-d-*galacto*-heptitol (8bb)

Prepared from 4b (110 mg, 0.19 mmol) and 7b (110 μL, 0.95 mmol) using DPPA (3 × 4.9 mg, 0.019 mmol) in toluene (1.9 mL) according to Method D to give 8bb by column chromatography (eluent: hexane–acetone = 8 : 1) as a yellow amorphous solid (70 mg, 51%). *R*_f_ = 0.43 (hexane–EtOAc = 2 : 1). [*α*]_D_ = +25.2 (*c* = 0.110, CHCl_3_).

^1^H-NMR of 8bb (400 MHz, CDCl_3_) *δ*: 8.14 (dd, 2H, *J* = 8.2, 1.5 Hz, aromatic), 7.97 (dd, 2H, *J* = 8.4, 1.4 Hz, aromatic), 7.92 (dd, 2H, *J* = 8.4, 1.3 Hz, aromatic), 7.79 (dd, 2H, *J* = 8.3, 1.5 Hz, aromatic), 7.62–7.13 (m, 19H, aromatic), 6.01 (pseudo t, 1H, *J* = 10.0 Hz, H-5), 5.89 (dd, 1H, *J* = 3.2, 0.8 Hz, H-3), 5.56 (dd, 1H, *J* = 10.0, 3.2 Hz, H-4), 4.78 (dd, 1H, *J* = 12.2, 2.5 Hz, H-7_A_), 4.46 (dd, 1H, *J* = 12.2, 4.6 Hz, H-7_B_), 4.07 (ddd, 1H, *J* = 10.0, 4.6, 2.5 Hz, H-6), 3.95 (ddd, 1H, *J* = 7.8, 5.5, 0.8 Hz, H-2), 3.86 (d, 1H, *J* = 13.4 Hz, CH_2_Ph), 3.70 (d, 1H, *J* = 13.4 Hz, CH_2_Ph), 2.73 (dd, 1H, *J* = 14.4, 7.8 Hz, H-1_A_), 2.51 (dd, 1H, *J* = 14.4, 5.5 Hz, H-1_B_). ^13^C-NMR of 8bb (100 MHz, CDCl_3_) *δ*: 166.3, 165.8, 165.6, 165.0 (CO), 138.1–127.2 (aromatic), 78.7, 76.6, 73.4, 70.1, 66.9 (C-2, C-3, C-4, C-5, C-6), 63.2 (C-7), 37.2 (CH_2_Ph), 31.1 (C-1). ESI-MS positive mode (*m*/*z*): calcd for C_42_H_36_NaO_9_S^+^ [M + Na]^+^ = 739.1972. Found: [M + Na]^+^ = 739.1973.

##### 4.1.4.8. 2,6-Anhydro-3,4,5,7-tetra-*O*-benzoyl-1-*S*-(2,3,4-tri-*O*-acetyl-β-d-xylopyranosyl)-1-thio-d-*glycero*-d-*galacto*-heptitol (8bd)

Prepared from 4b (70 mg, 0.12 mmol) and 7d (40 mg, 0.13 mmol) using DPPA (3 × 3.1 mg, 0.012 mmol) in toluene (1.2 mL) according to Method D to give 8bd by column chromatography (eluent: hexane–acetone = 5 : 1) as a yellow amorphous solid (60 mg, 68%). *R*_f_ = 0.33 (hexane–EtOAc = 2 : 1). [*α*]_D_ = +0.5 (*c* = 0.162, CHCl_3_).

^1^H-NMR of 8bd (400 MHz, CDCl_3_) *δ*: 8.13 (dd, 2H, *J* = 8.1, 1.1 Hz, aromatic), 8.02 (dd, 2H, *J* = 8.2, 1.2 Hz, aromatic), 7.91 (dd, 2H, *J* = 8.3, 1.4 Hz, aromatic), 7.79 (dd, 2H, *J* = 8.5, 1.3 Hz, aromatic), 7.62–7.23 (m, 12H, aromatic), 6.04 (pseudo t, 1H, *J* = 10.1 Hz, H-5), 6.00 (dd, 1H, *J* = 3.1, 0.9 Hz, H-3), 5.62 (dd, 1H, *J* = 10.1, 3.1 Hz, H-4), 5.14 (pseudo t, 1H, *J* = 8.3 Hz, H-2′), 4.94 (pseudo t, 1H, *J* = 8.2 Hz, H-3′), 4.92–4.87 (m, 1H, H-4′), 4.75 (dd, 1H, *J* = 12.2, 2.5 Hz, H-7_A_), 4.55 (d, 1H, *J* = 8.3 Hz, H-1′), 4.45 (dd, 1H, *J* = 12.2, 4.2 Hz, H-7_B_), 4.16–4.10 (m, 3H, H-2, H-6, 
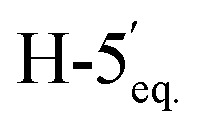
), 3.38 (dd, 1H, *J* = 11.7, 8.9 Hz, 
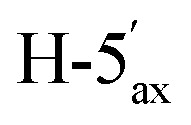
), 2.99 (dd, 1H, *J* = 14.0, 6.6 Hz, H-1_A_), 2.84 (dd, 1H, *J* = 14.0, 7.1 Hz, H-1_B_), 2.04, 2.03, 2.02 (3 × s, 3 × 3H, OAc). ^13^C-NMR of 8bd (100 MHz, CDCl_3_) *δ*: 170.1, 170.0, 169.8, 169.6, 166.3, 165.5, 165.4 (CO), 133.5–128.4 (aromatic), 83.7, 77.6, 76.5, 73.4, 72.0, 70.1, 69.5, 68.6, 66.9, (C-2, C-3, C-4, C-5, C-6, C-1′, C-2′, C-3′, C-4′), 65.4 (C-7), 63.1 (C-5′), 30.4 (C-1), 20.8 (OAc). ESI-MS positive mode (*m*/*z*): calcd for C_46_H_44_NaO_16_S^+^ [M + Na]^+^ = 907.2242. Found: [M + Na]^+^ = 907.2238.

##### 4.1.4.9. 2,6-Anhydro-3,4,5,7-tetra-*O*-benzoyl-1-*S*-(2,3,4-tri-*O*-acetyl-β-d-xylopyranosyl)-1-thio-d-*glycero*-d-*galacto*-heptitol-*S*-oxide (9bd)

Prepared from 4b (70 mg, 0.12 mmol) and 7d (40 mg, 0.13 mmol) using DPPA (3 × 3.1 mg, 0.012 mmol) in toluene (1.2 mL) according to Method D to give 9bd by column chromatography (eluent: hexane–acetone = 5 : 1) as a yellow amorphous solid (11 mg, 12%). *R*_f_ = 0.21 (hexane–acetone = 1 : 2).

^1^H-NMR of 9bd (400 MHz, CDCl_3_) *δ*: 8.13 (dd, 2H, *J* = 8.2, 1.1 Hz, aromatic), 8.05 (dd, 2H, *J* = 8.2, 1.2 Hz, aromatic), 7.92 (dd, 2H, *J* = 8.2, 1.3 Hz, aromatic), 7.78 (dd, 2H, *J* = 8.4, 1.3 Hz, aromatic), 7.65–7.22 (m, 12H, aromatic), 6.08 (pseudo t, 1H, *J* = 10.0 Hz, H-5), 5.87 (dd, 1H, *J* = 3.2, 0.9 Hz, H-3), 5.66 (dd, 1H, *J* = 10.1, 3.2 Hz, H-4), 5.27 (pseudo t, 1H, *J* = 8.2 Hz, H-2′), 5.21 (pseudo t, 1H, *J* = 8.2 Hz, H-3′), 4.92 (ddd, 1H, *J* = 9.0, 8.2, 5.1 Hz, H-4′), 4.74 (dd, 1H, *J* = 12.2, 2.5 Hz, H-7_A_), 4.55 (ddd, 1H, *J* = 10.7, 2.3, 0.9 Hz, H-2), 4.47 (dd, 1H, *J* = 12.2, 4.5 Hz, H-7_B_), 4.41 (d, 1H, *J* = 8.3 Hz, H-1′), 4.23–4.18 (m, 2H, H-6, 
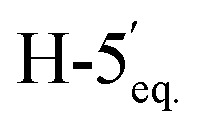
), 3.47 (dd, 1H, *J* = 11.6, 9.0 Hz, 
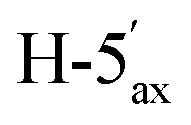
), 3.17 (dd, 1H, *J* = 13.0, 10.7 Hz, H-1_A_), 3.04 (dd, 1H, *J* = 13.0, 2.3 Hz, H-1_B_), 2.03, 2.02, 1.96 (3 × s, 3 × 3H, OAc). ESI-MS positive mode (*m*/*z*): calcd for C_46_H_44_KO_17_S^+^ [M + K]^+^ = 939.1936. Found: [M + K]^+^ = 939.1934.

##### 4.1.4.10. 2,6-Anhydro-3,4,5,7-tetra-*O*-benzoyl-1-*S*-(1,2:3,4-di-*O*-isopropylidene-β-d-galactopyranos-6-yl)-1-thio-d-*glycero*-d-*galacto*-heptitol (8be)

Prepared from 4b (50 mg, 0.08 mmol) and 7e (24 mg, 0.088 mmol) using DPPA (3 × 2.1 mg, 0.008 mmol) in toluene (0.8 mL) according to Method D to give 8be by column chromatography (eluent: hexane–acetone = 4 : 1) as a yellow amorphous solid (51 mg, 73%). *R*_f_ = 0.35 (hexane–EtOAc = 2 : 1). [*α*]_D_ = −22.3 (*c* = 0.160, CHCl_3_).

^1^H-NMR of 8be (400 MHz, CDCl_3_) *δ*: 8.11 (dd, 2H, *J* = 8.4, 1.4 Hz, aromatic), 8.04 (dd, 2H, *J* = 8.1, 1.0 Hz, aromatic), 7.92 (dd, 2H, *J* = 8.0, 0.9 Hz, aromatic), 7.78 (dd, 2H, *J* = 8.0, 0.9 Hz, aromatic), 7.62–7.22 (m, 12H, aromatic), 6.03 (dd, 1H, *J* = 3.2, 0.7 Hz, H-3), 6.01 (pseudo t, 1H, *J* = 10.0 Hz, H-5), 5.68 (dd, 1H, *J* = 10.0, 3.2 Hz, H-4), 5.52 (d, 1H, *J* = 5.0 Hz, H-1′), 4.73 (dd, 1H, *J* = 12.2, 2.7 Hz, H-7_A_), 4.58 (dd, 1H, *J* = 7.9, 2.4 Hz, H-3′), 4.46 (dd, 1H, *J* = 12.2, 4.6 Hz, H-7_B_), 4.28 (dd, 1H, *J* = 5.0, 2.4 Hz, H-2′), 4.24 (dd, 1H, *J* = 7.9, 1.8 Hz, H-4′), 4.23 (m, 1H, H-2), 4.15 (ddd, 1H, *J* = 10.0, 4.6, 2.7 Hz, H-6), 3.86 (ddd, 1H, *J* = 7.6, 5.8, 1.8 Hz, H-5′), 2.91 (dd, 1H, *J* = 14.1, 7.0 Hz, H-1_A_), 2-86-2.74 (m, 3H, H-1_B_, 
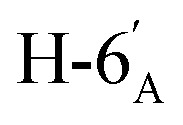
, 
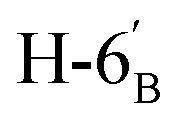
), 1.46, 1.41, 1.29, 1.27 (4 × s, 4 × 3H, CH_3_). ^13^C-NMR of 8be (100 MHz, CDCl_3_) *δ*: 166.3, 165.7, 165.6 (CO), 133.5–128.4 (aromatic), 109.4, 108.7 (C_acetal_), 96.7 (C-1′), 78.2, 76.5, 73.3, 72.0, 71.0, 70.6, 70.0, 68.3, 67.3 (C-2, C-3, C-4, C-5, C-6, C-2′, C-3′, C-4′, C-5′), 63.5 (C-7), 33.4 (C-1), 33.0 (C-6′), 26.2, 26.1, 25.0, 24.5 (CH_3_). ESI-MS positive mode (*m*/*z*): calcd for C_47_H_48_NaO_14_S^+^ [M + Na]^+^ = 891.2657. Found: [M + Na]^+^ = 891.2659.

##### 4.1.4.11. 2,5-Anhydro-3,4:6,7-di-*O*-isopropylidene-1-*S*-phenyl-1-thio-d-*glycero*-d-*galacto*-heptitol (10a)

Prepared from 6 (60 mg, 0.23 mmol) and 7a (230 μL, 2.3 mmol) using DPPA (4 × 5.9 mg, 0.023 mmol) in toluene (2.3 mL) at room temperature according to Method D to give 10a by column chromatography (eluent: hexane–acetone = 10 : 1) as a yellow amorphous solid (45 mg; 53%).

Prepared from 6 (56 mg, 0.22 mmol) and 7a (220 μL, 2.2 mmol) using DPPA (2 × 5.6 mg, 0.022 mmol) in toluene (2.2 mL) at −78 °C according to Method D to give 10a by column chromatography (eluent: hexane–acetone = 10 : 1) as a yellow amorphous solid (56 mg; 69%). *R*_f_ = 0.44 (hexane–EtOAc = 2 : 1). [*α*]_D_ = +39.9 (*c* = 0.172, CHCl_3_).

^1^H-NMR of 10a (400 MHz, CDCl_3_) *δ*: 7.39 (dd, 2H, *J* = 8.3, 1.2 Hz, aromatic), 7.30–7.26 (m, 2H, aromatic), 7.21–7.17 (m, 1H, aromatic), 4.76–4.72 (m, 2H, H-3, H-4), 4.38 (ddd, 1H, *J* = 7.5, 6.0, 4.7 Hz, H-6), 4.07 (dd, 1H, *J* = 8.7, 6.0 Hz, H-7_A_), 4.03 (dd, 1H, *J* = 8.7, 4.7 Hz, H-7_B_), 3.67 (ddd, 1H, *J* = 7.7, 6.1, 2.9 Hz, H-2), 3.49 (dd, 1H, *J* = 7.5, 2.9 Hz, H-5), 3.24 (dd, 1H, *J* = 13.5, 6.1 Hz, H-1_A_), 3.22 (dd, 1H, *J* = 13.5, 7.7 Hz, H-1_B_), 1.49, 1.43, 1.37, 1.34 (4 × s, 4 × 3H, CH_3_). ^13^C-NMR of 10a (100 MHz, CDCl_3_) *δ*: 136.0–126.3 (aromatic), 112.7, 109.2 (C_acetal_), 81.8, 80.8, 80.6, 80.6, 73.2 (C-2, C-3, C-4, C-5, C-6), 66.9 (C-7), 31.4 (C-1), 27.1, 25.9, 25.3, 24.7 (CH_3_). ESI-MS positive mode (*m*/*z*): calcd for C_19_H_27_O_5_S^+^ [M + H]^+^ = 367.0987. Found: [M + H]^+^ = 367.1012.

##### 4.1.4.12. 2,5-Anhydro-3,4:6,7-di-*O*-isopropylidene-1-*S*-(2,3,4,6-tetra-*O*-acetyl-β-d-glucopyranosyl)-1-thio-d-*glycero*-d-*galacto*-heptitol (10c)

Prepared from 6 (79 mg, 0.31 mmol) and 7c (135 mg, 0.37 mmol) using DPPA (3 × 7.9 mg, 0.031 mmol) in toluene (3.1 mL) according to Method D to give 10c by column chromatography (eluent: hexane–acetone = 4 : 1) as a colorless syrup (168 mg; 88%). *R*_f_ = 0.28 (hexane–EtOAc = 2 : 1). [*α*]_D_ = −6.4 (*c* = 0.200, CHCl_3_).

^1^H NMR of 10c (400 MHz, CDCl_3_) *δ*: 5.21 (pseudo t, 1H, *J* = 9.3 Hz, H-3′), 5.08 (pseudo t, 1H, *J* = 9.7 Hz, H-4′), 5.07 (pseudo t, 1H, *J* = 9.6 Hz, H-2′), 4.79–4.73 (m, 2H, H-3, H-4), 4.61 (d, 1H, *J* = 10.1 Hz, H-1′), 4.38 (dt, 1H, *J* = 6.1, 5.0 Hz, H-6), 4.24 (dd, 1H, *J* = 12.4, 5.0 Hz, 
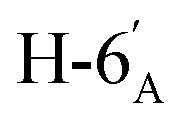
), 4.14 (dd, 1H, *J* = 12.4, 2.2 Hz, 
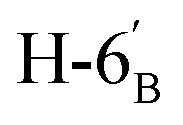
), 4.12–4.00 (m, 2H, H-7_A_, H-7_B_), 3.77 (ddd, 1H, *J* = 8.5, 5.2, 2.8 Hz, H-2), 3.70 (ddd, 1H, *J* = 10.1, 5.0, 2.2 Hz, H-5′), 3.55 (dd, 1H, *J* = 7.2, 2.8 Hz, H-5), 3.07 (dd, 1H, *J* = 13.8, 8.5 Hz, H-1_A_), 2.85 (dd, 1H, *J* = 13.8, 5.2 Hz, H-1_B_), 2.09 2.06, 2.03, 2.01 (4 × s, 4 × 3H, OAc), 1.47, 1.44, 1.37, 1.36 (4 × s, 4 × 3H, CH_3_). ^13^C NMR of 10c (100 MHz, CDCl_3_) *δ*:170.7, 170.4, 169.6, 169.5 (CO), 112.7, 109.1 (C_acetal_), 84.2, 82.1, 81.8, 80.8, 80.7, 76.0, 74.0, 73.2, 70.2, 68.4 (C-2, C-3, C-4, C-5, C-6, C-1′, C-2′, C-3′, C-4′, C-5′), 66.9 (C-7), 62.2 (C-6′), 28.4 (C-1), 27.0, 25.9, 25.3, 24.9 (OAc), 20.9, 20.8, 20.7, 20.7 (CH_3_). Elemental analysis: calcd for C_27_H_40_O_14_S (620.663): C: 52.25; H: 6.50; S: 5.17. Found: C: 50.28; H: 6.66; S: 5.12.

##### 4.1.4.13. 2,5-Anhydro-3,4:6,7-di-*O*-isopropylidene-1-*S*-(1,2:3,4-di-*O*-isopropylidene-β-d-galactopyranose-6-yl)-1-thio-d-*glycero*-d-*galacto*-heptitol (10e)

Prepared from 6 (111 mg, 0.40 mmol) and 7e (100 mg, 0.36 mmol) using DPPA (3 × 10.3 mg, 0.040 mmol) in toluene (4.0 mL) according to Method D to give 10e by column chromatography (eluent: hexane–acetone = 4 : 1) as a colorless syrup (154 mg; 81%). *R*_f_ = 0.36 (hexane–EtOAc = 2 : 1). [*α*]_D_ = −28.7 (*c* = 0.300, CHCl_3_).

^1^H NMR of 10e (400 MHz, CDCl_3_) *δ*: 5.53 (d, 1H, *J* = 5.0 Hz, H-1′), 4.77–4.70 (m, 2H, H-3, H-4), 4.62 (dd, 1H, *J* = 7.9, 2.2 Hz, H-3′), 4.35–4.28 (m, 2H, H-2′, H-4′), 4.38 (dd, 1H, *J* = 12.1, 6.1 Hz, H-6), 4.12–4.01 (m, 2H, H-7_A_, H-7_B_), 3.87 (pseudo t, 1H, *J* = 6.8 Hz, H-2), 3.71 (td, 1H, *J* = 6.8, 2.8 Hz, H-5′), 3.51 (dd, 1H, *J* = 7.6, 2.9 Hz, H-5), 2.95–2.78 (m, 4H, H-1_A_, H-1_B_, 
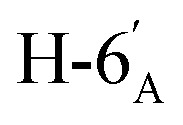
, 
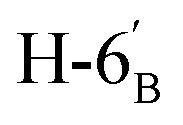
), 1.53, 1.45, 1.44, 1.37, 1.35, 1.33 (8 × s, 8 × 3H, CH_3_). ^13^C NMR of 10e (100 MHz, CDCl_3_) *δ*: 112.5, 109.3, 109.1, 108.6 (C_acetal_), 96.7 (C-1′), 82.1, 81.9, 80.9, 80.6, 73.1, 71.7, 71.0, 70.6, 67.6 (C-2, C-3, C-4, C-5, C-6, C-2′, C-3′, C-4′, C-5′), 67.0 (C-7′), 32.8 (C-6′) 30.4 (C-1), 27.0, 26.2, 26.1, 25.8, 25.3, 25.0, 24.7, 24.5 (CH_3_). Elemental analysis: calcd for C_25_H_40_O_10_S (532.65): C: 56.37; H: 7.57; S: 6.02. Found: C: 58.31; H: 7.75; S: 5.94.

##### 4.1.4.14. 2,5-Anhydro-3,4:6,7-di-*O*-isopropylidene-1-*S*-(2,3,4,6-tetra-*O*-acetyl-β-d-mannopyranosyl)-1-thio-d-*glycero*-d-*galacto*-heptitol (10f)

Prepared from 6 (123 mg, 0.48 mmol) and 7f (210 mg, 0.58 mmol) using DPPA (3 × 12.3 mg, 0.048 mmol) in toluene (4.8 mL) according to Method D to give 10f by column chromatography (eluent: hexane–acetone = 4 : 1) as a colorless syrup (168 mg; 88%). *R*_f_ = 0.34 (hexane–EtOAc = 2 : 1). [*α*]_D_ = −15.8 (*c* = 0.300, CHCl_3_).

^1^H NMR of 10f (400 MHz, CDCl_3_) *δ*: 5.53 (d, 1H, *J* = 2.5 Hz, H-3), 5.25 (pseudo t, 1H, *J* = 10.0 Hz, H-4′), 5.04 (dd, 1H, *J* = 10.1, 3.4 Hz, H-2′), 4.92 (s, 1H, H-1′), 4.80–4.74 (m, 2H, H-3, H-4), 4.38 (dd, 1H, *J* = 11.7, 6.2 Hz, H-6), 4.26 (dd, 1H, *J* = 12.3, 5.9 Hz, 
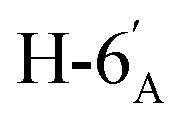
), 4.15 (dd, 1H, *J* = 12.3, 1.9 Hz, 
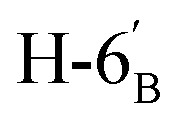
), 4.10–3.98 (m, 2H, H-7_A_, H-7_B_), 3.78–3.71 (m, 1H, H-2), 3.70–3.64 (m, 1H, H-5′), 3.55 (dd, 1H, *J* = 6.9, 2.2 Hz, H-5), 3.04 (dd, 1H, *J* = 13.6, 9.1 Hz, H-1_A_), 2.94 (dd, 1H, *J* = 13.6, 5.1 Hz, H-1_B_), 2.19, 2.08, 2.05, 1.98 (4 × s, 4 × 3H, OAc), 1.47, 1.44, 1.37, 1.35 (4 × s, 4 × 3H, CH_3_). ^13^C NMR of 10f (100 MHz, CDCl_3_) *δ*: 170.7, 170.3, 170.2, 169.7 (CO), 112.7, 109.1 (C_acetal_), 83.6, 81.9, 80.7, 80.5, 76.7, 73.2, 72.0, 70.4, 65.9 (C-2, C-3, C-4, C-5, C-6, C-1′, C-2′, C-3′, C-4′, C-5′), 66.8 (C-7), 63.0 (C-6′), 29.4 (C-1), 27.0, 25.8, 25.3, 24.8 (OAc), 20.9, 20.8, 20.8, 20.7 (CH_3_). Elemental analysis: calcd for C_27_H_40_O_14_S (620.66): C: 52.25; H: 6.50; S: 5.17. Found: C: 52.06; H: 6.26; S: 5.21.

## Funding

This research was funded by the National Research, Development and Innovation Office of Hungary (Grant K109450, K132870 and FK128766), and by the EU co-financed by the European Regional Development Fund under the projects GINOP-2.3.2-15-2016-00008 and GINOP-2.3.3-15-2016-00004.

## Author contributions

JJ: synthesis and structure elucidation of compounds (1a, b – 4a, b; every adduct except 8ac, 8ae, 10c, 10a, 10f) wrote the manuscript; ND: synthesis and structure elucidation of compounds (8ac, 8ae); ED: synthesis and structure elucidation of compounds (10c, 10e 10f); AB: planned and controlled the experiments, structure elucidations (8ac, 8ae, 10c, 10a, 10f) and reviewed manuscript; LJ: planned and controlled the experiments, structure elucidations, wrote the manuscript; SL: conceived the research and wrote the manuscript. All authors have read and agreed to the published version of the manuscript.

## Conflicts of interest

The authors declare no conflict of interest. The funders had no role in the design of the study; in the collection, analyses, or interpretation of data; in the writing of the manuscript, or in the decision to publish the results.

## Supplementary Material

RA-010-D0RA07115C-s001
