# Supplementary material for: Synthesis and photoinitiated thiol–ene reactions of exo-mannals – a new route to C-β-d-mannosyl derivatives
Source: RSC Adv. 2020 Sep 22;10(57):34825–36. doi: 10.1039/d0ra07115c (PMC9056834; doi:10.1039/d0ra07115c)
Supplement: RA-010-D0RA07115C-s001 [file RA-010-D0RA07115C-s001.pdf]

Electronic Supplementary Material (ESI) for RSC Advances.
This journal is © The Royal Society of Chemistry 2020

Supporting information

Synthesis and Photoinitiated Thiol-Ene Reactions of *Exo*-Mannals – A New Route to *C*-β-D-Mannosyl Derivatives

János József[1,3], Nóra Debreczeni[2,3,4], Dániel Eszenyi[2], Anikó Borbás[2], László Juhász[1,*], László Somsák[1,*]

1 Department of Organic Chemistry, University of Debrecen, PO Box 400, H-4002 Debrecen, Hungary
2 Department of Pharmaceutical Chemistry, University of Debrecen, PO Box 400, H-4002 Debrecen, Hungary
3 University of Debrecen, Doctoral School of Chemistry, PO Box 400, H-4002 Debrecen, Hungary
4 HAS-UD Molecular Recognition and Interaction Research Group, University of Debrecen, Egyetem tér 1, Debrecen 4032, Hungary
* Correspondace: juhasz.laszlo@science.unideb.hu; Tel: +36-52-512900/22464 (L.J.); somsak.laszlo@science.unideb.hu; Tel: +36-52-512900/22348 (L.S.)

Copies of the NMR spectra for the new compounds

The ^{1}H (400 MHz) and ^{13}C NMR (100.28 MHz) NMR spectra were recorded with Bruker DRX-400 spectrometer, respectively. Chemical shifts are referenced to Me_4Si (0.00 ppm for 1H) and to the residual solvent signals ($CDCl_3$: 77.16 ppm for ^{13}C). The coupling constant values (*J*) are given in Hz.

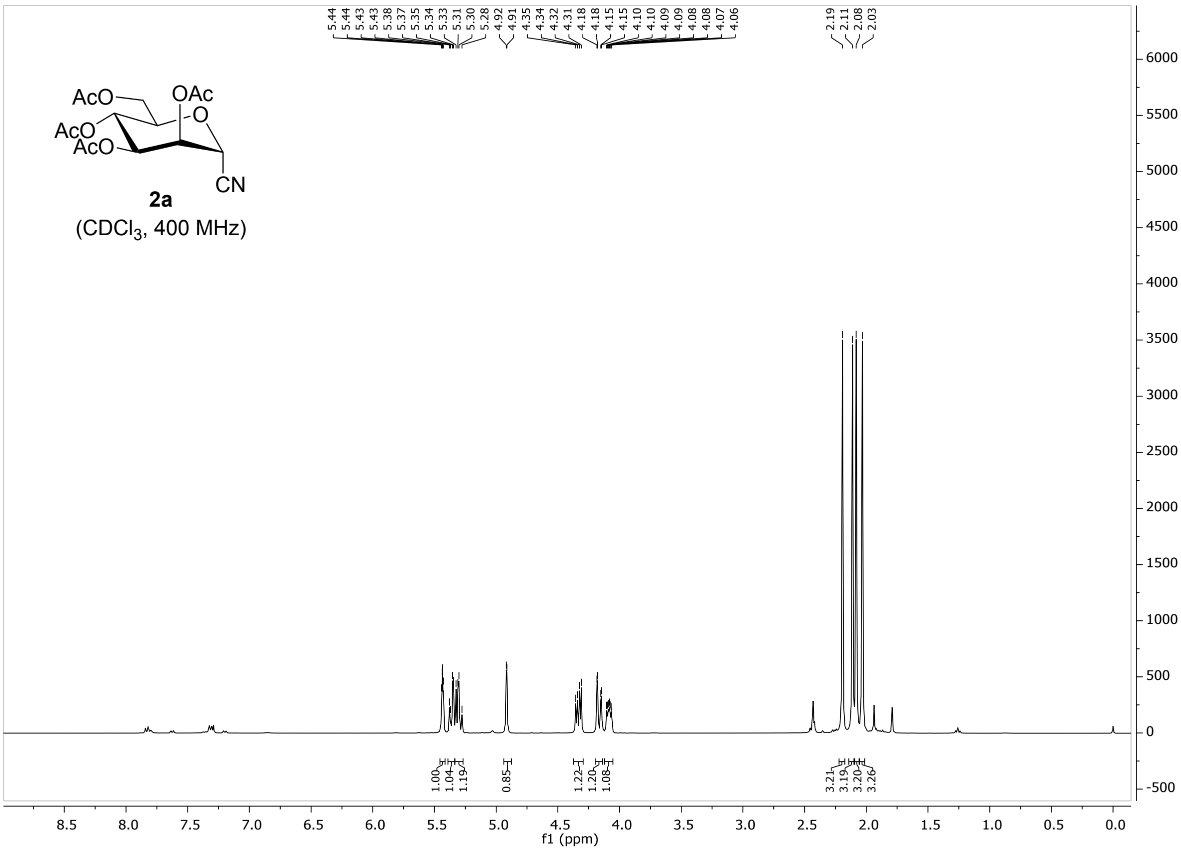
AcO
OAc
O
AcO
AcO
CN
2a
(CDCl$_3$, 400 MHz)
5.44 5.44 5.43 5.38 5.37 5.35 5.34 5.33 5.31 5.30 5.28 4.92 4.91 4.35 4.34 4.32 4.31 4.18 4.18 4.15 4.15 4.10 4.09 4.09 4.08 4.08 4.07 4.06
2.19 2.11 2.08 2.03
1.00 1.04 1.19 0.85 1.22 1.20 1.08 3.21 3.19 3.20 3.26
f1 (ppm)

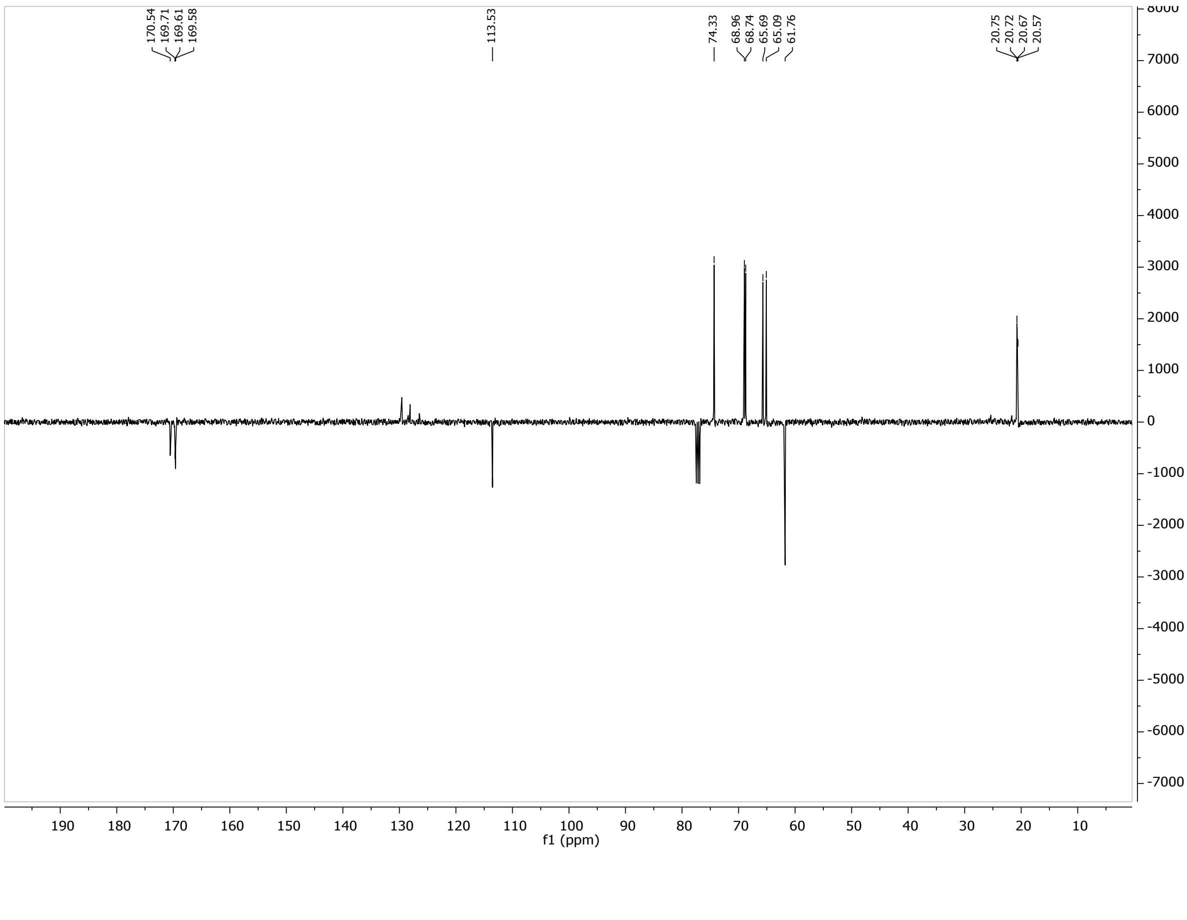
170.54 169.71 169.61 169.58
113.53
74.33 68.96 68.74 65.69 65.09 61.76
20.75 20.72 20.67 20.57
f1 (ppm)

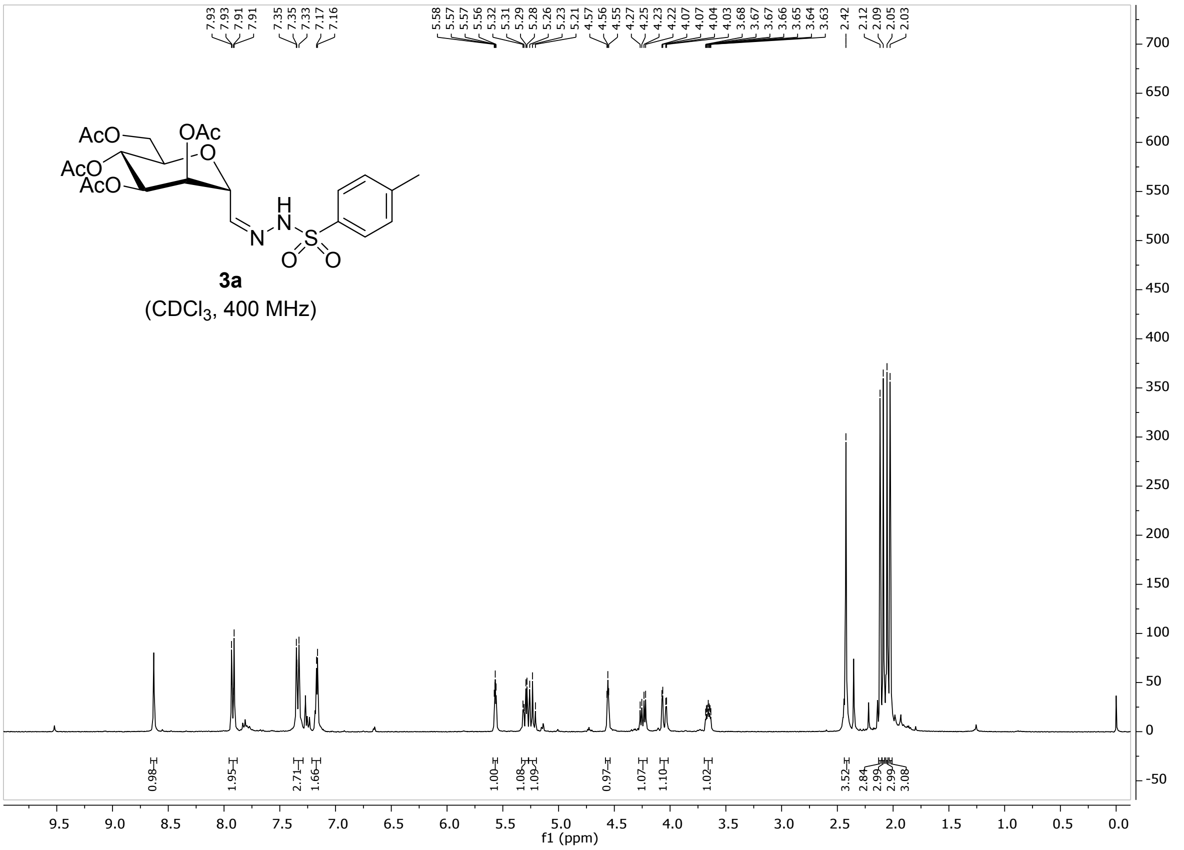
AcO
OAc
AcO
AcO
O
H
N
N
S
O
O
3a
(CDCl3, 400 MHz)
f1 (ppm)

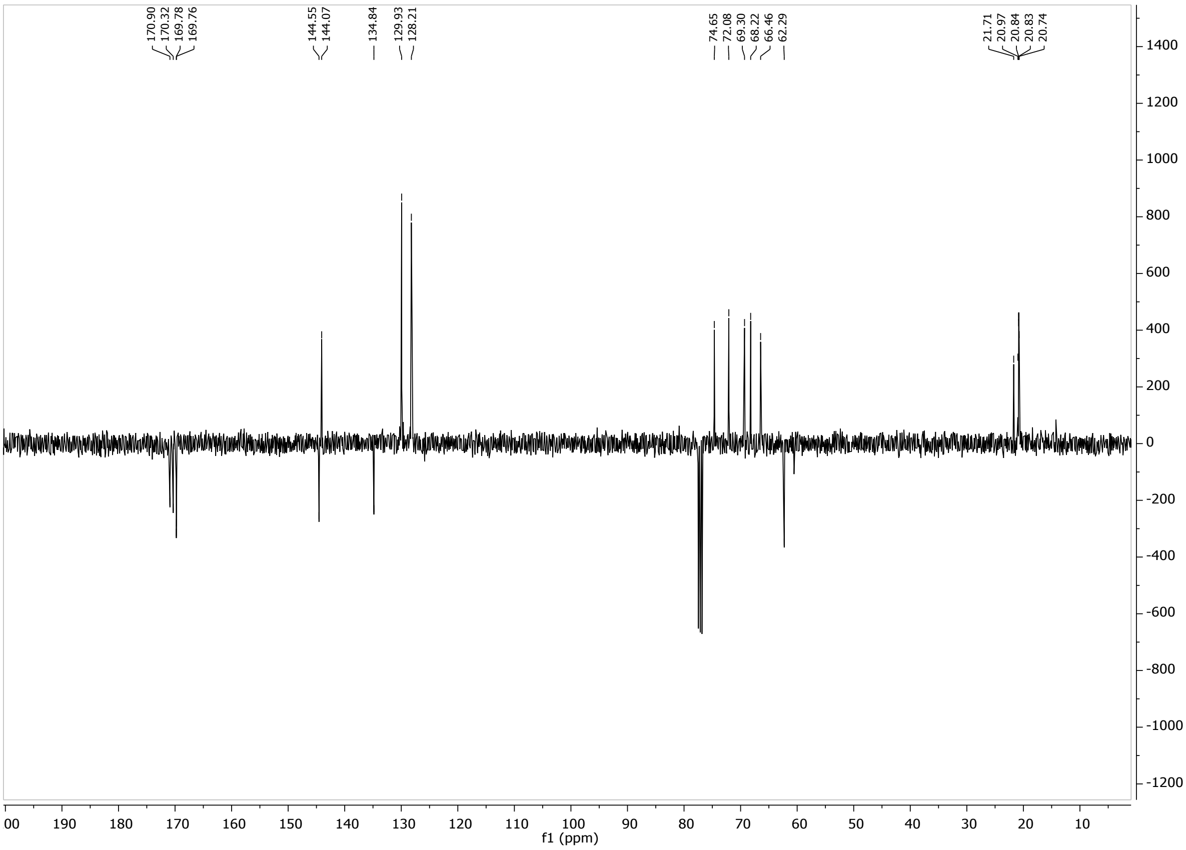
f1 (ppm)

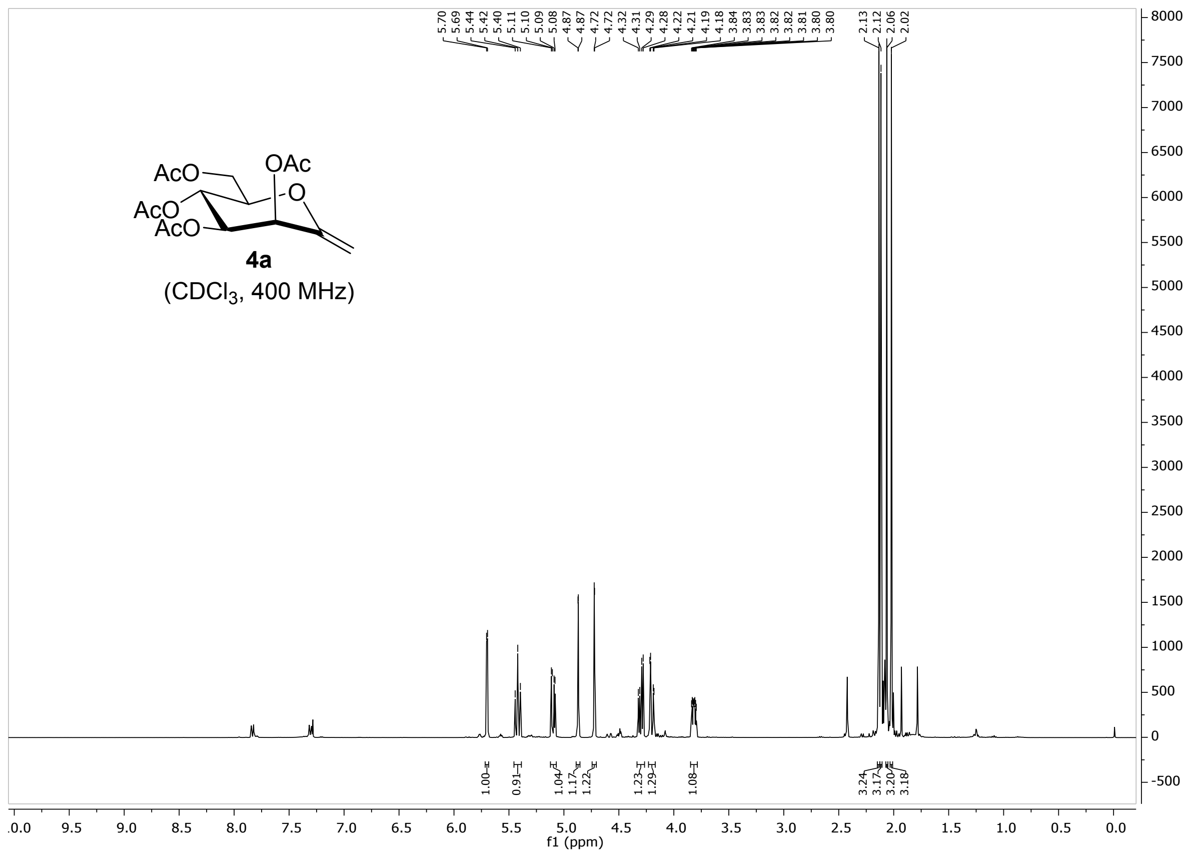
5.70 5.69 5.44 5.42 5.40 5.11 5.10 5.09 5.08 4.87 4.87 4.72 4.72 4.32 4.31 4.29 4.28 4.22 4.21 4.19 4.18 3.84 3.83 3.83 3.82 3.82 3.81 3.80 3.80
2.13 2.12 2.06 2.02
AcO
OAc
AcO
AcO
O
4a
(CDCl3, 400 MHz)
1.00 0.91 1.04 1.17 1.22 1.23 1.29 1.08 3.24 3.17 3.20 3.18
f1 (ppm)

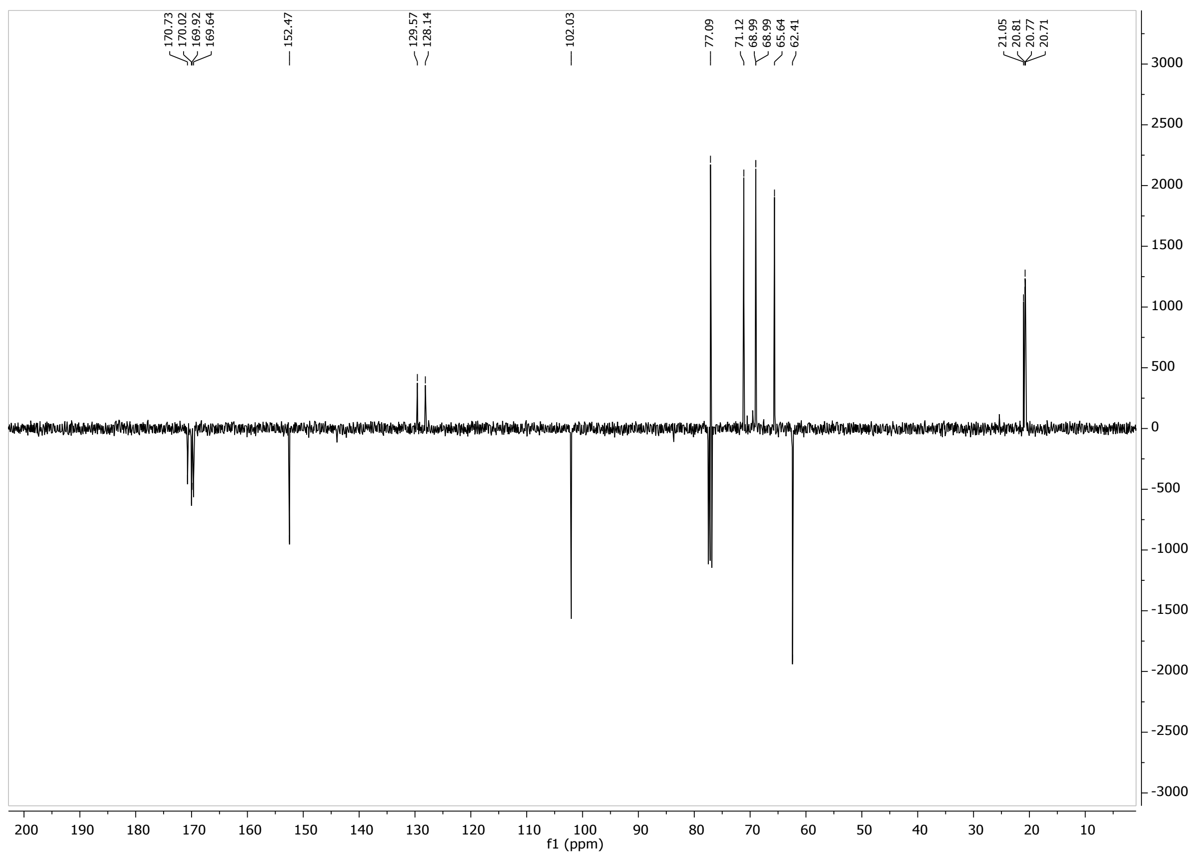
170.73 170.02 169.92 169.64
152.47
129.57 128.14
102.03
77.09
71.12 68.99 68.99 65.64 62.41
21.05 20.81 20.77 20.71
f1 (ppm)

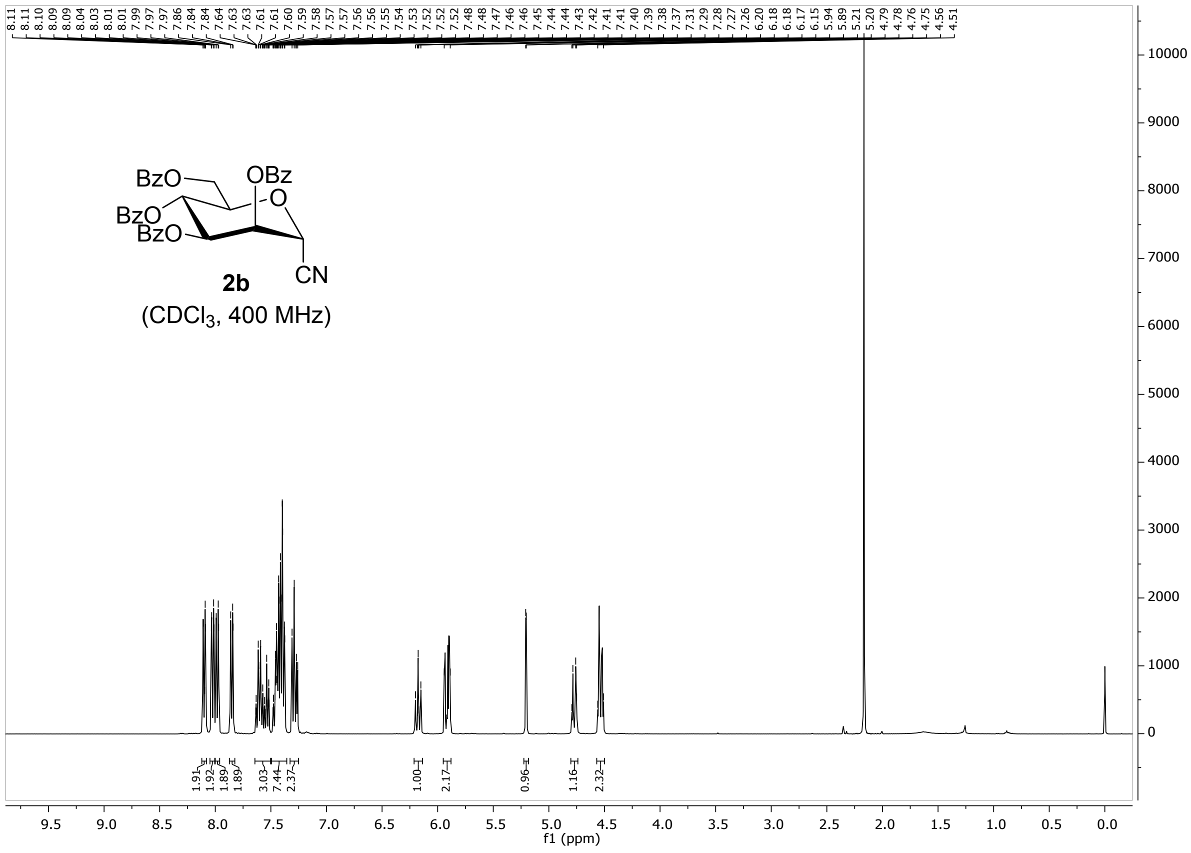
BzO
OBz
O
BzO
BzO
CN
2b
(CDCl$_3$, 400 MHz)
1.91 1.92 1.89 1.89 3.03 7.44 2.37 1.00 2.17 0.96 1.16 2.32
f1 (ppm)

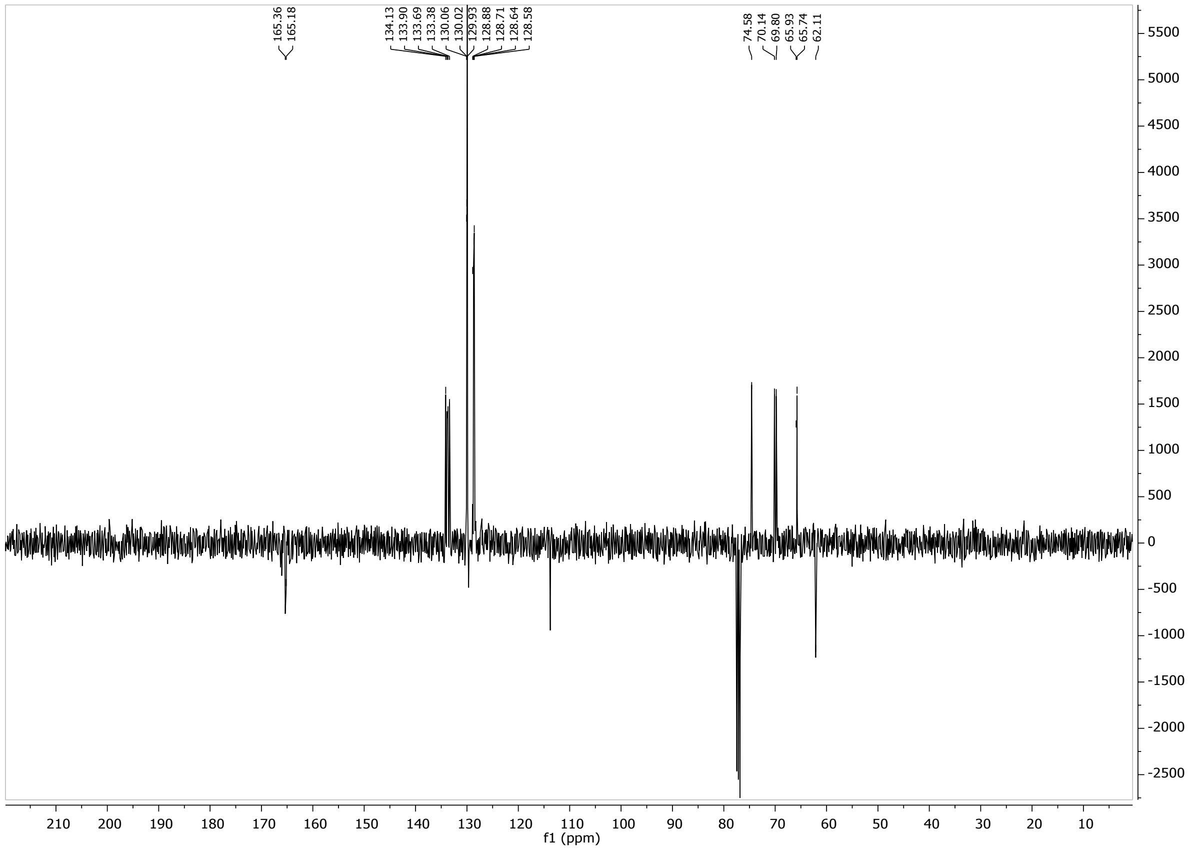
165.36 165.18
134.13 133.90 133.69 133.38 130.06 130.02 129.93 128.88 128.71 128.64 128.58
74.58 70.14 69.80 65.93 65.74 62.11
f1 (ppm)

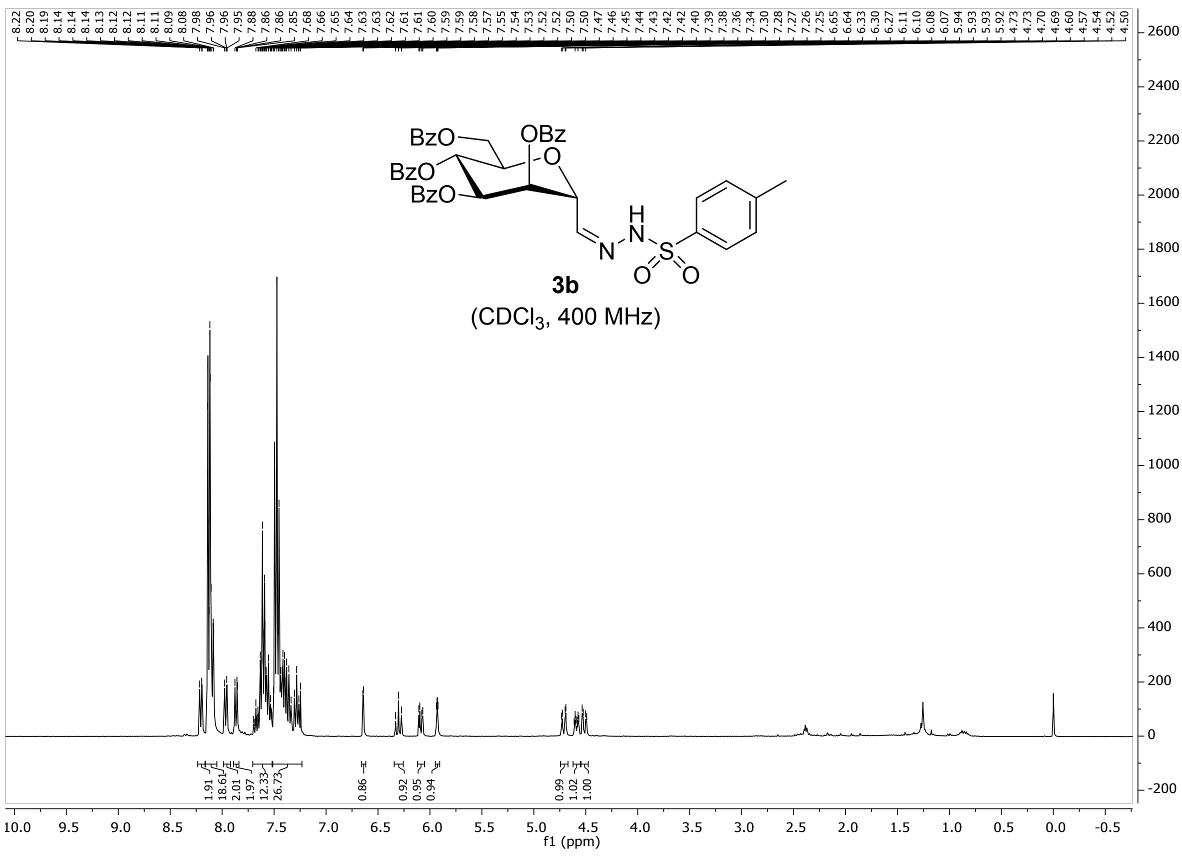
BzO
OBz
O
BzO
BzO
H
N
N
S
O
O
3b
(CDCl3, 400 MHz)
f1 (ppm)

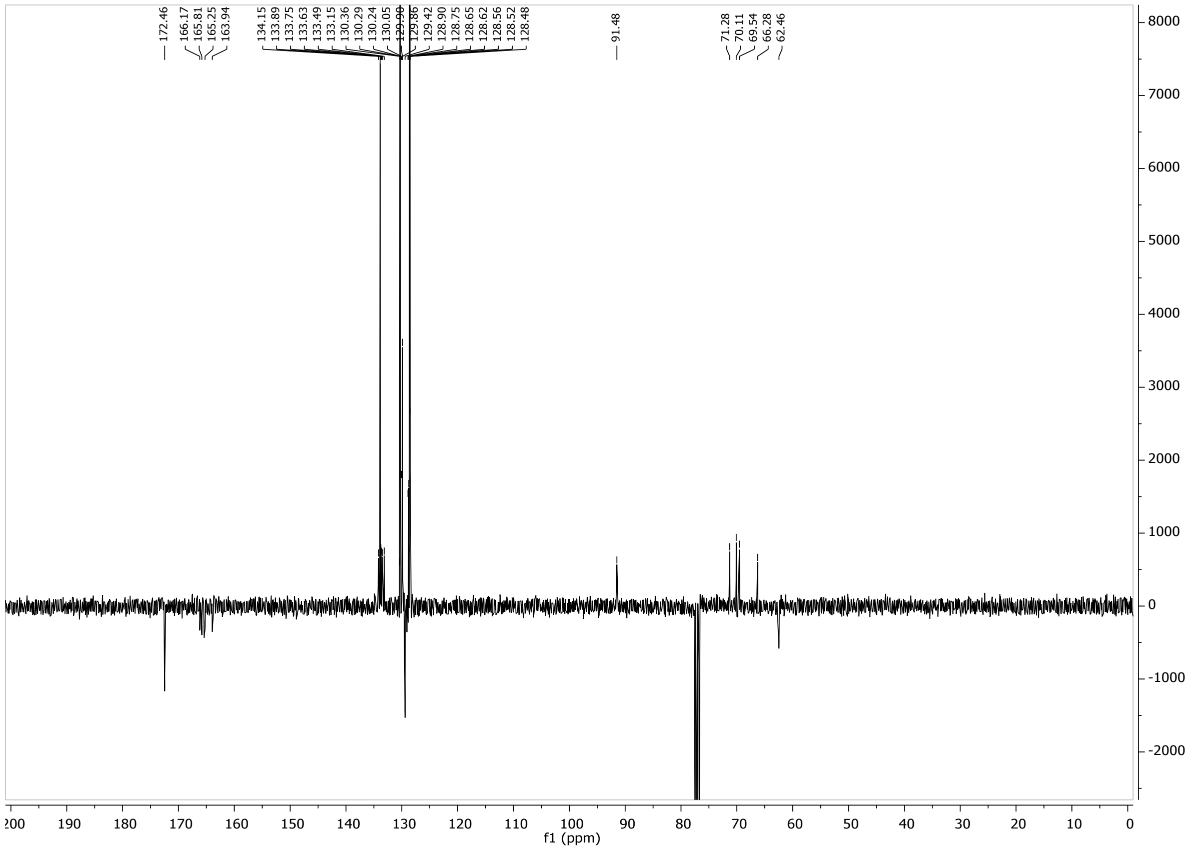
f1 (ppm)

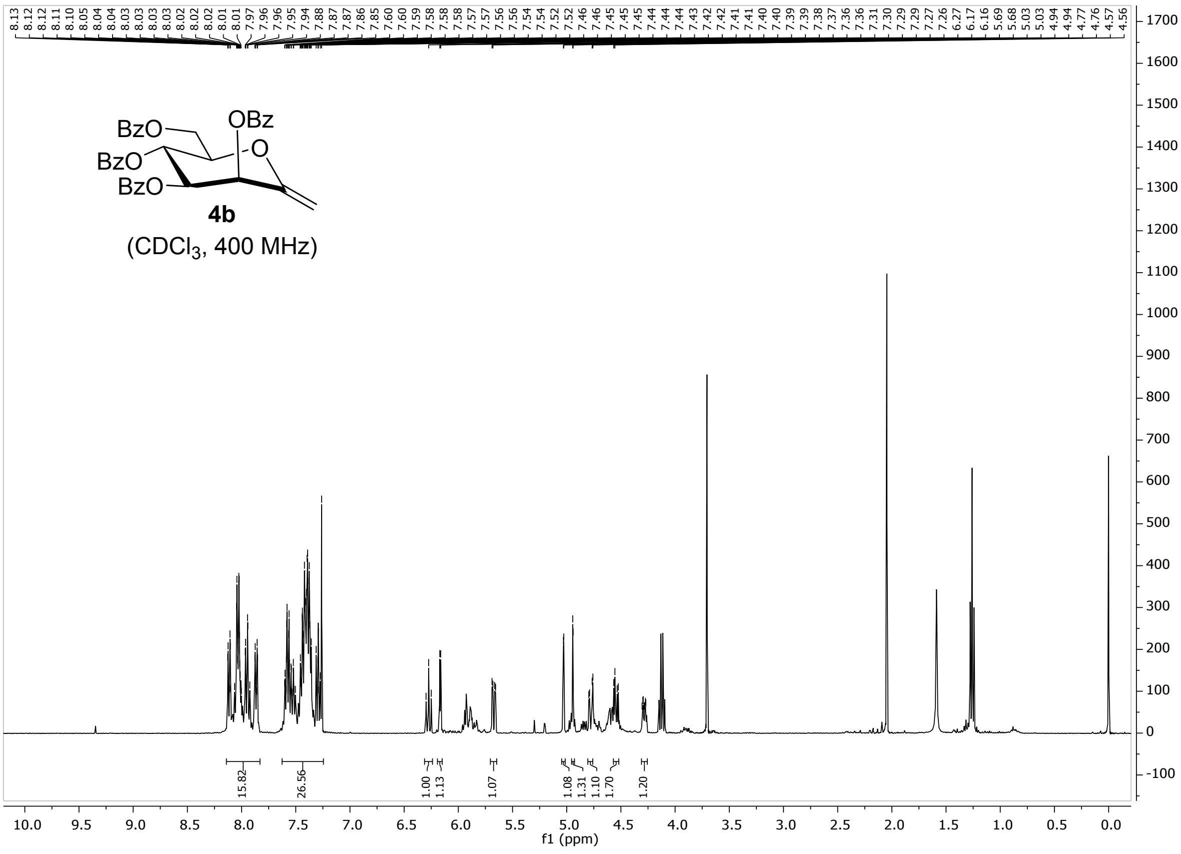

8.13 8.12 8.11 8.10 8.05 8.04 8.04 8.03 8.03 8.03 8.02 8.02 8.02 8.01 8.01 7.97 7.96 7.96 7.95 7.94 7.88 7.87 7.87 7.86 7.85 7.60 7.59 7.58 7.58 7.58 7.57 7.57 7.56 7.56 7.54 7.54 7.52 7.52 7.46 7.46 7.45 7.45 7.45 7.44 7.44 7.44 7.43 7.42 7.42 7.41 7.41 7.40 7.40 7.39 7.39 7.38 7.38 7.37 7.36 7.36 7.31 7.30 7.29 7.29 7.27 7.26 6.27 6.17 6.16 5.69 5.68 5.03 4.94 4.94 4.77 4.76 4.57 4.56
BzO
BzO
BzO
OBz
O
4b
(CDCl3, 400 MHz)
15.82 26.56 1.00 1.13 1.07 1.08 1.31 1.10 1.70 1.20
f1 (ppm)

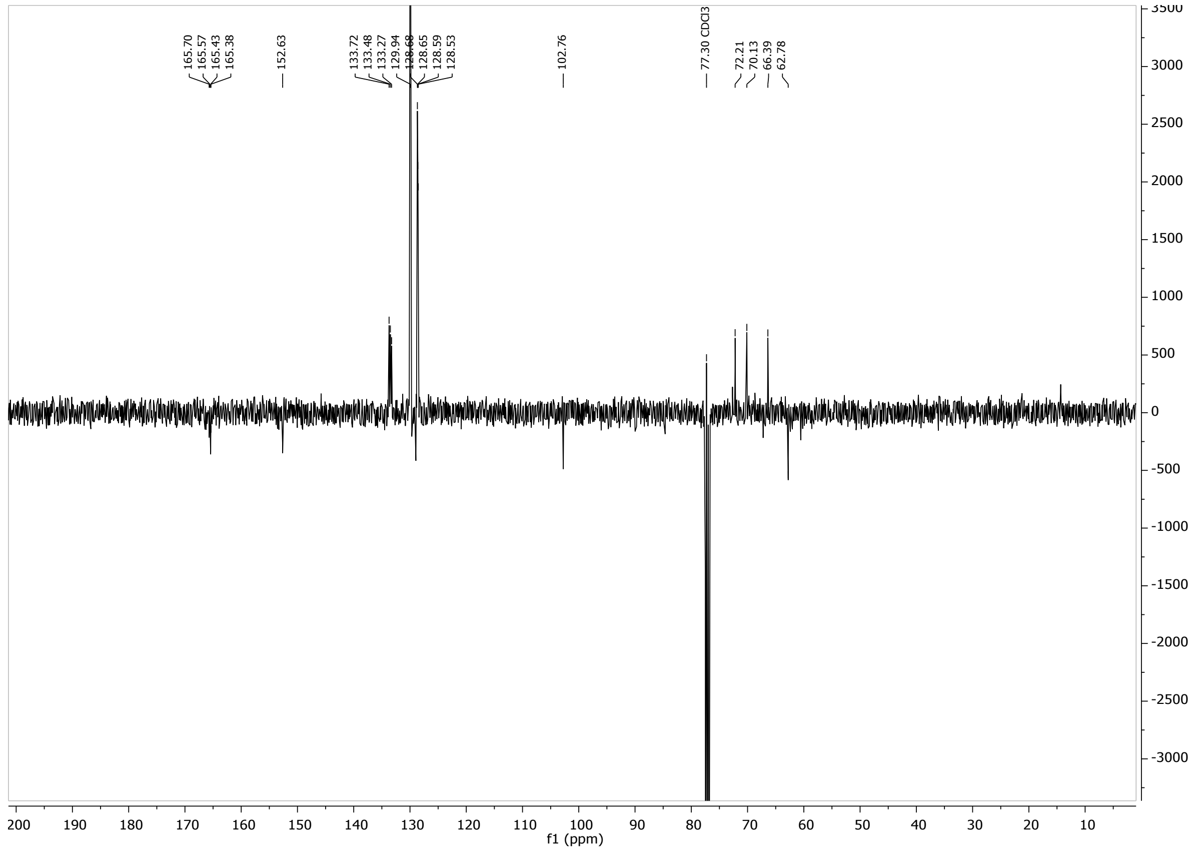

165.70 165.57 165.43 165.38
152.63
133.72 133.48 133.27 129.94 128.68 128.65 128.59 128.53
102.76
77.30 CDCl3
72.21 70.13 66.39 62.78
f1 (ppm)

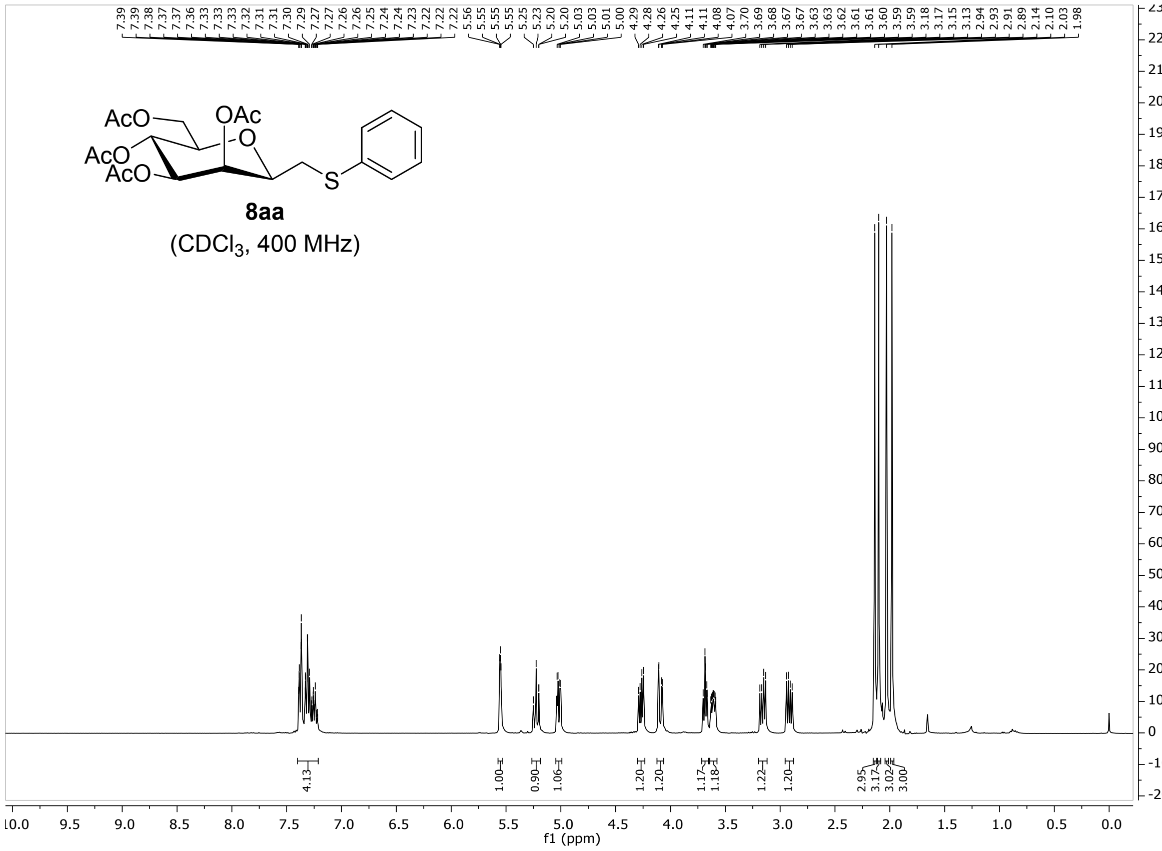
7.39 7.39 7.38 7.37 7.37 7.36 7.33 7.33 7.33 7.32 7.31 7.31 7.30 7.29 7.27 7.27 7.26 7.26 7.25 7.24 7.24 7.23 7.22 7.22 7.22 7.22 5.56 5.55 5.55 5.55 5.25 5.23 5.20 5.20 5.03 5.03 5.01 5.00 4.29 4.28 4.26 4.25 4.11 4.11 4.08 4.07 3.70 3.69 3.68 3.67 3.63 3.63 3.62 3.62 3.61 3.61 3.60 3.59 3.59 3.18 3.17 3.15 3.13 2.94 2.93 2.91 2.89 2.14 2.10 2.03 1.98
AcO
OAc
O
AcO
AcO
S
8aa
($CDCl_3$, 400 MHz)
4.13 1.00 0.90 1.06 1.20 1.20 1.17 1.18 1.22 1.20 2.95 3.17 3.02 3.00
f1 (ppm)

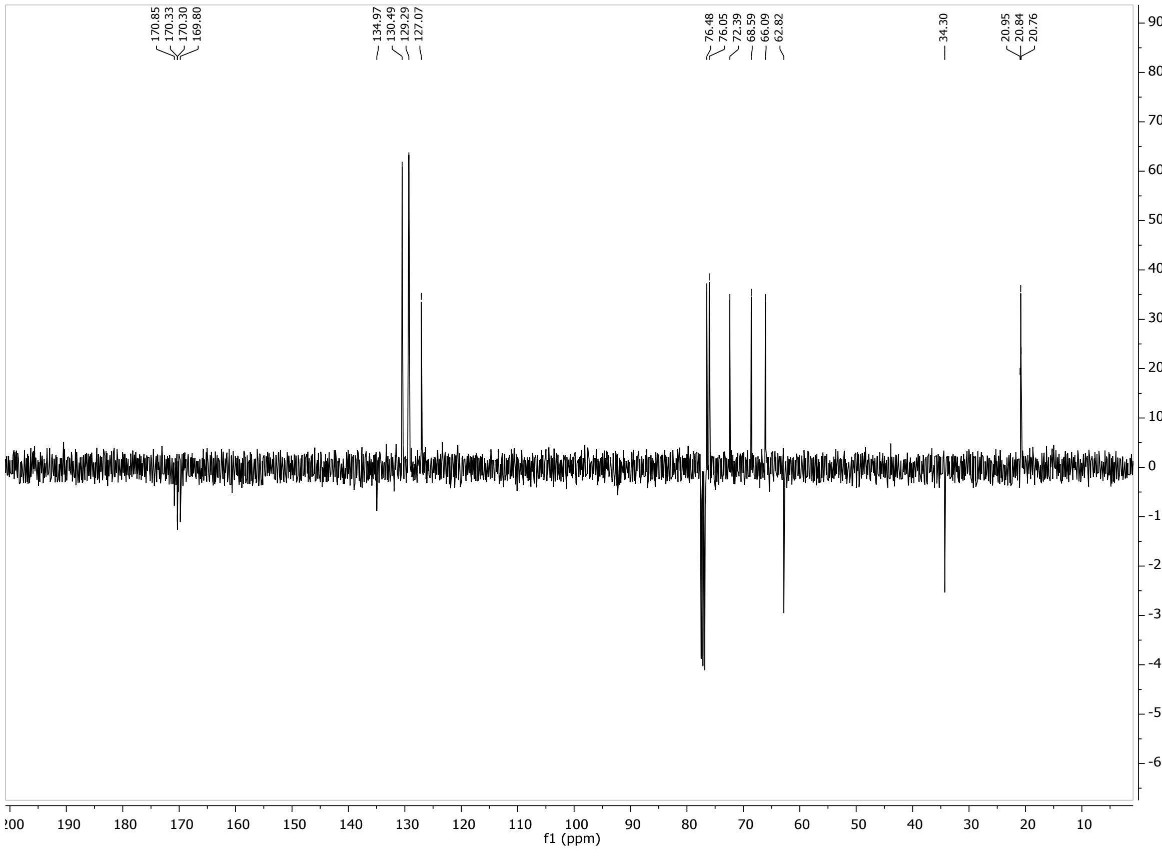
170.85 170.33 170.30 169.80
134.97 130.49 129.29 127.07
76.48 76.05 72.39 68.59 66.09 62.82
34.30
20.95 20.84 20.76
f1 (ppm)

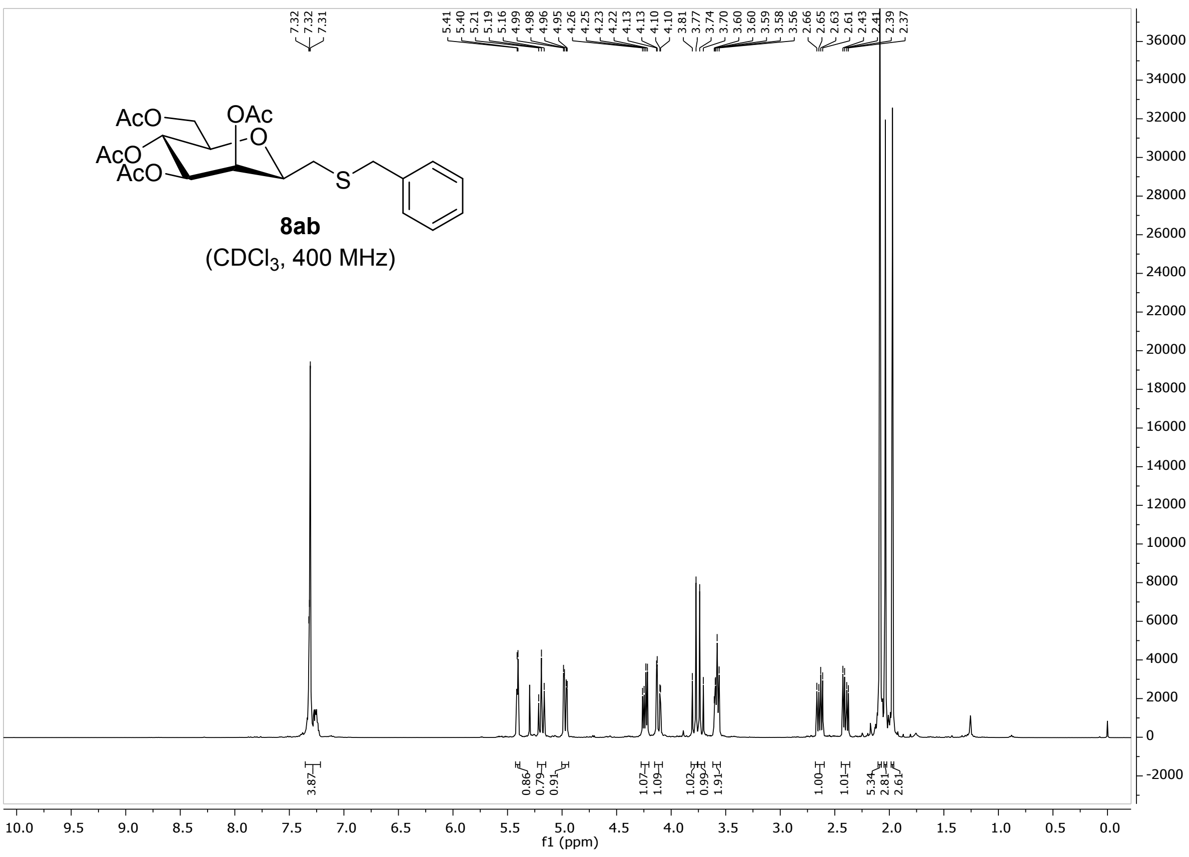
AcO
OAc
O
AcO
AcO
S
8ab
(CDCl3, 400 MHz)
7.32 7.32 7.31
5.41 5.40 5.21 5.19 5.16 4.99 4.98 4.96 4.95 4.26 4.25 4.23 4.22 4.13 4.13 4.10 4.10 3.81 3.77 3.74 3.70 3.60 3.60 3.59 3.58 3.56 2.66 2.65 2.63 2.61 2.43 2.41 2.39 2.37
3.87 0.86 0.79 0.91 1.07 1.09 1.02 0.99 1.91 1.00 1.01 5.34 2.81 2.61
f1 (ppm)

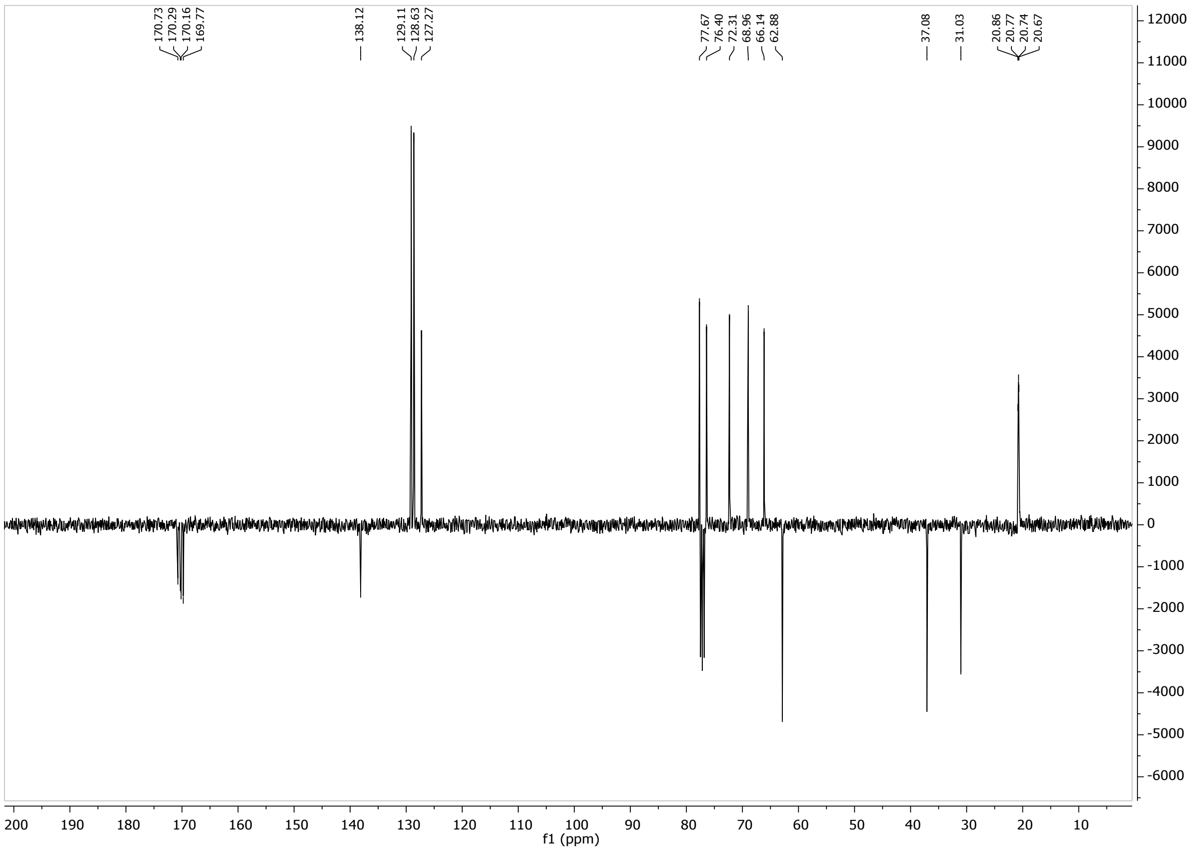
170.73 170.29 170.16 169.77
138.12
129.11 128.63 127.27
77.67 76.40 72.31 68.96 66.14 62.88
37.08
31.03
20.86 20.77 20.74 20.67
f1 (ppm)

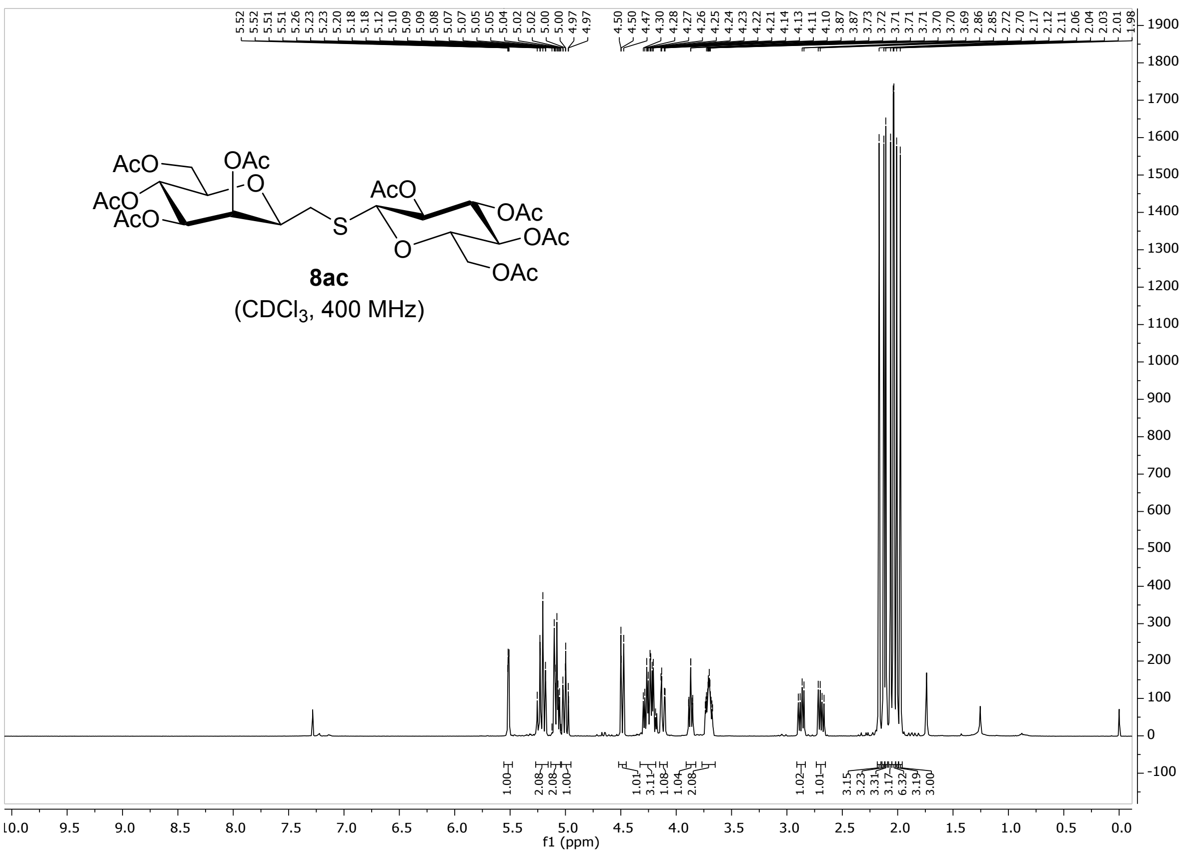

8ac
(CDCl$_3$, 400 MHz)
f1 (ppm)

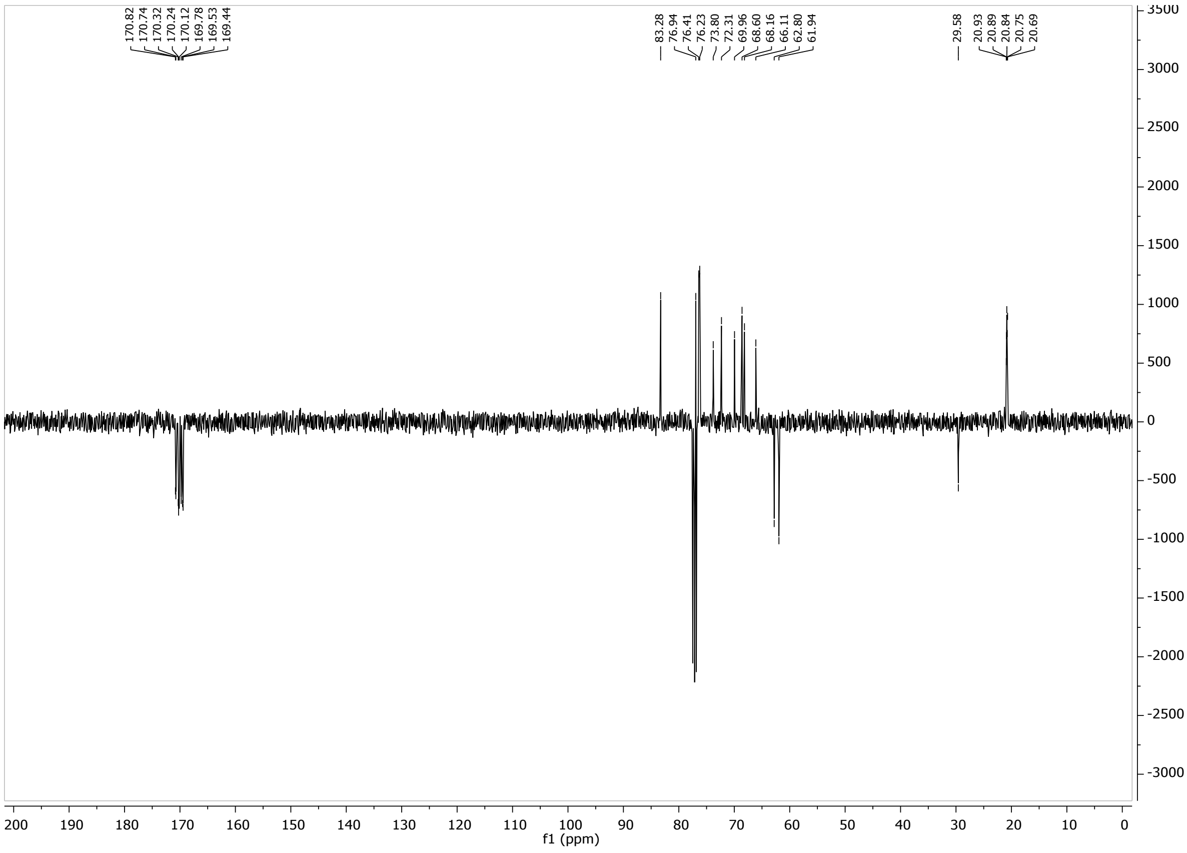

f1 (ppm)

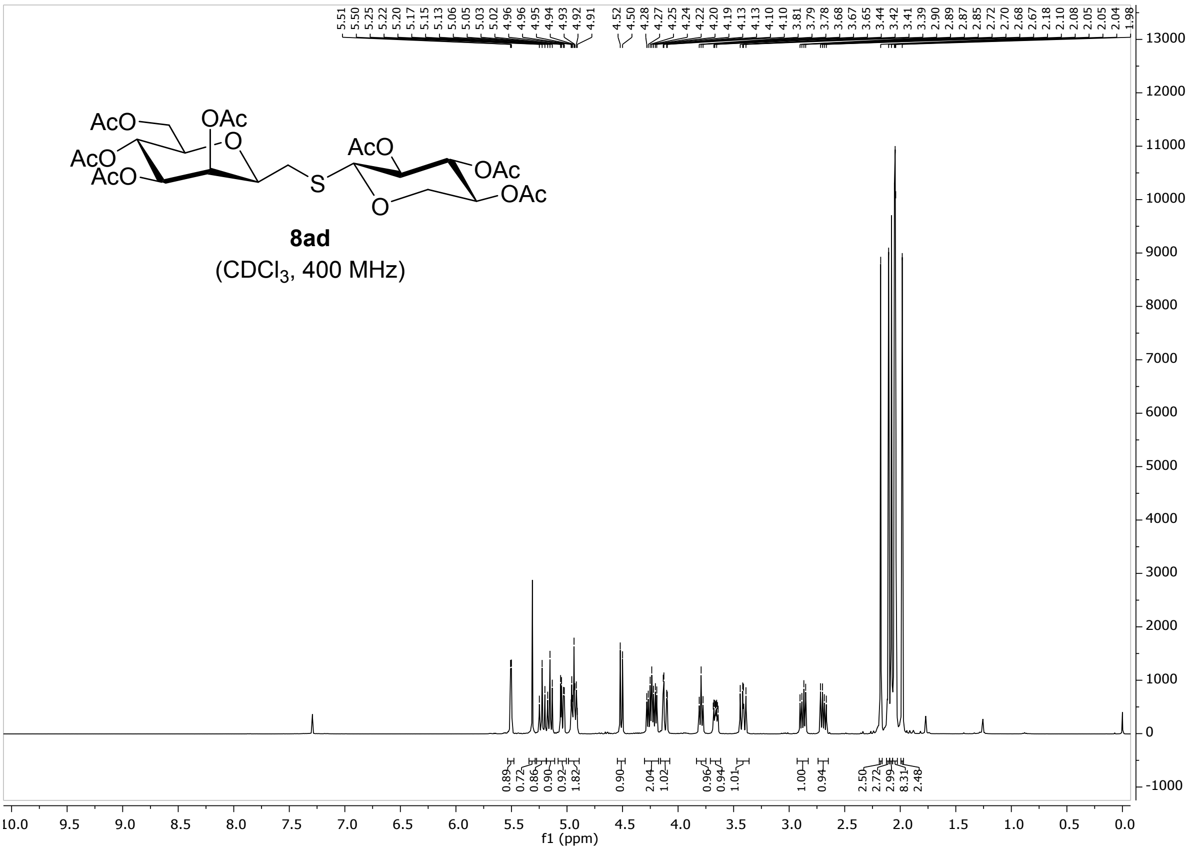
AcO
OAc
AcO
AcO
S
AcO
OAc
OAc
O
O
8ad
($CDCl_3$, 400 MHz)
5.51 5.50 5.25 5.22 5.20 5.17 5.15 5.13 5.06 5.05 5.03 5.02 4.96 4.95 4.94 4.93 4.92 4.91 4.52 4.50 4.28 4.27 4.25 4.24 4.22 4.20 4.19 4.13 4.13 4.10 4.10 3.81 3.79 3.78 3.68 3.67 3.65 3.44 3.42 3.41 3.39 2.90 2.89 2.87 2.85 2.72 2.70 2.68 2.67 2.18 2.10 2.08 2.05 2.05 2.04 1.98
0.89 0.72 0.86 0.90 0.92 1.82 0.90 2.04 1.02 0.96 0.94 1.01 1.00 0.94 2.50 2.72 2.99 8.31 2.48
f1 (ppm)

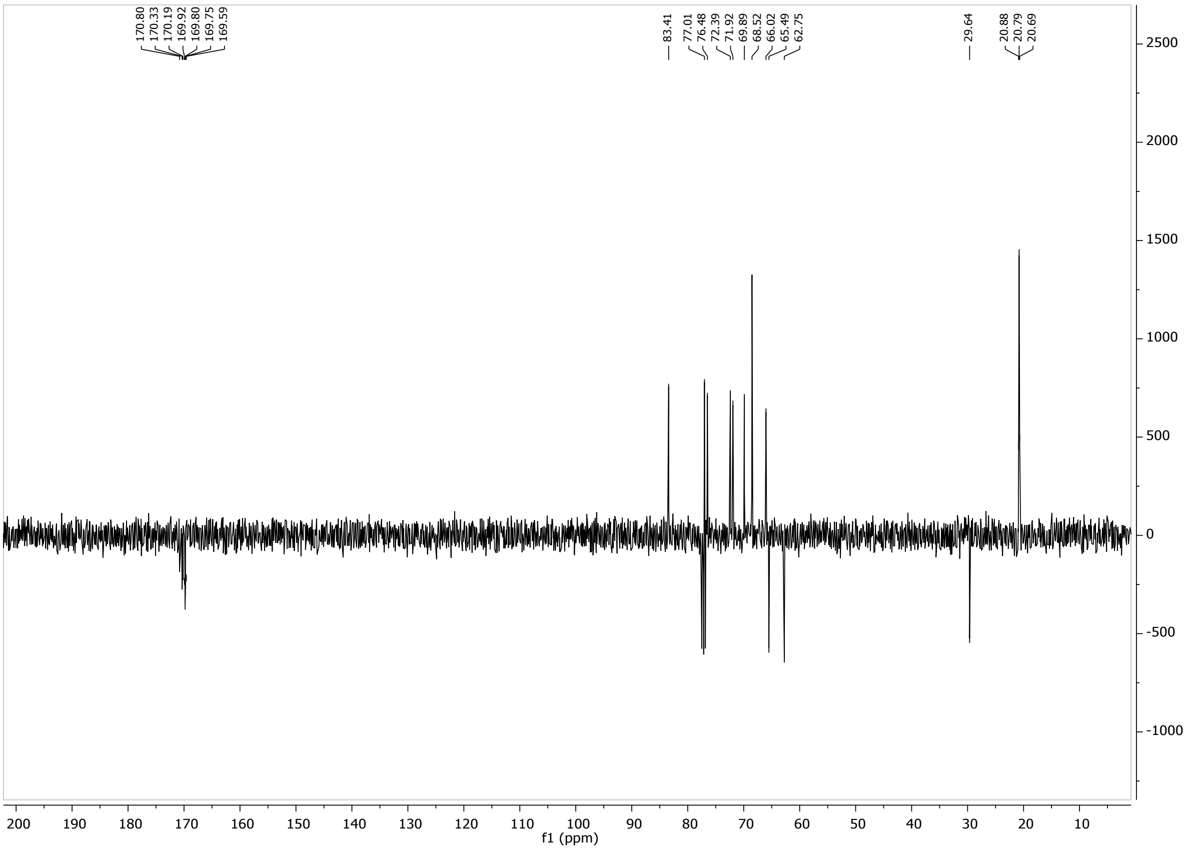
170.80 170.33 170.19 169.92 169.80 169.75 169.59
83.41 77.01 76.48 72.39 71.92 69.89 68.52 66.02 65.49 62.75
29.64 20.88 20.79 20.69
f1 (ppm)

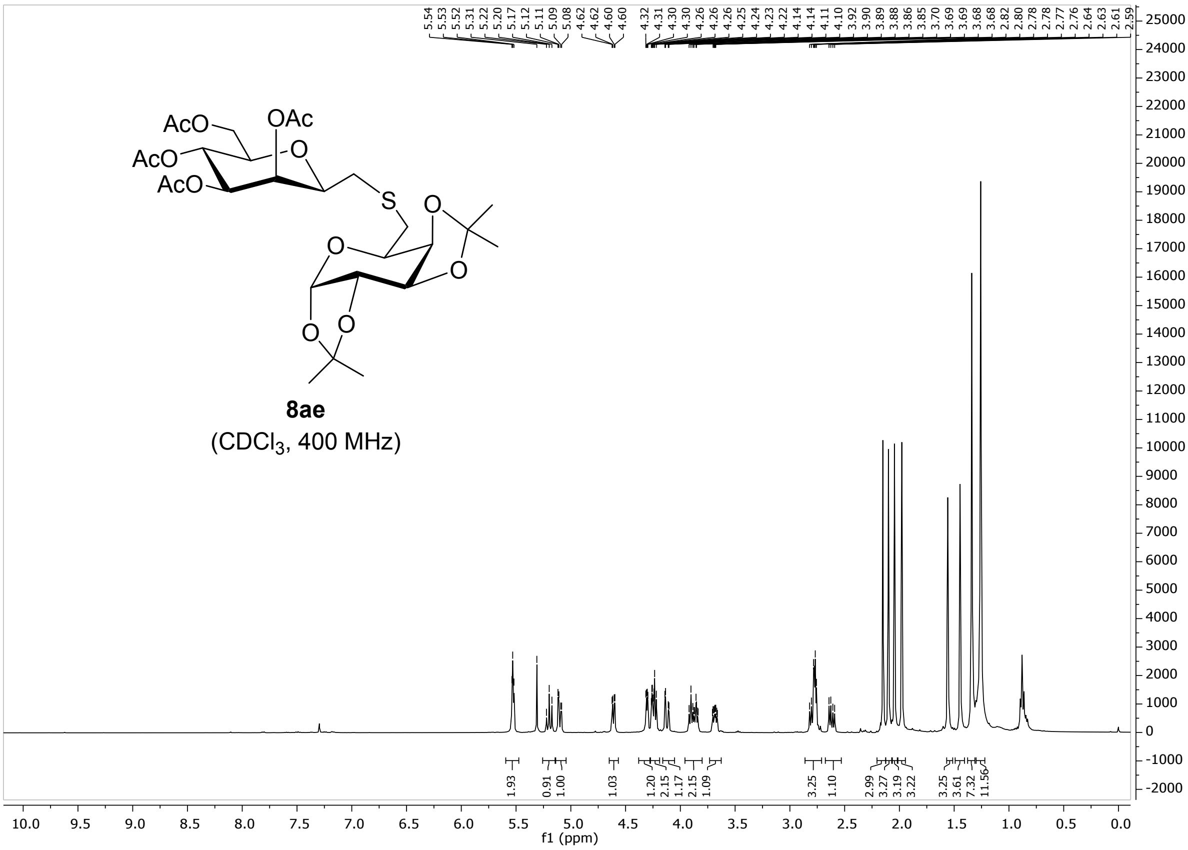

5.54 5.53 5.52 5.31 5.22 5.20 5.17 5.12 5.11 5.09 5.08 4.62 4.62 4.60 4.60 4.32 4.31 4.30 4.30 4.26 4.26 4.26 4.25 4.24 4.23 4.22 4.14 4.14 4.11 4.10 3.92 3.90 3.89 3.88 3.86 3.85 3.70 3.69 3.69 3.68 3.68 2.82 2.80 2.78 2.77 2.76 2.64 2.63 2.61 2.59
AcO
AcO
AcO
OAc
O
S
O
O
O
O
O
8ae
(CDCl$_3$, 400 MHz)
1.93 0.91 1.00 1.03 1.20 2.15 1.17 2.15 1.09 3.25 1.10 2.99 3.27 3.19 3.22 3.25 3.61 7.32 11.56
f1 (ppm)

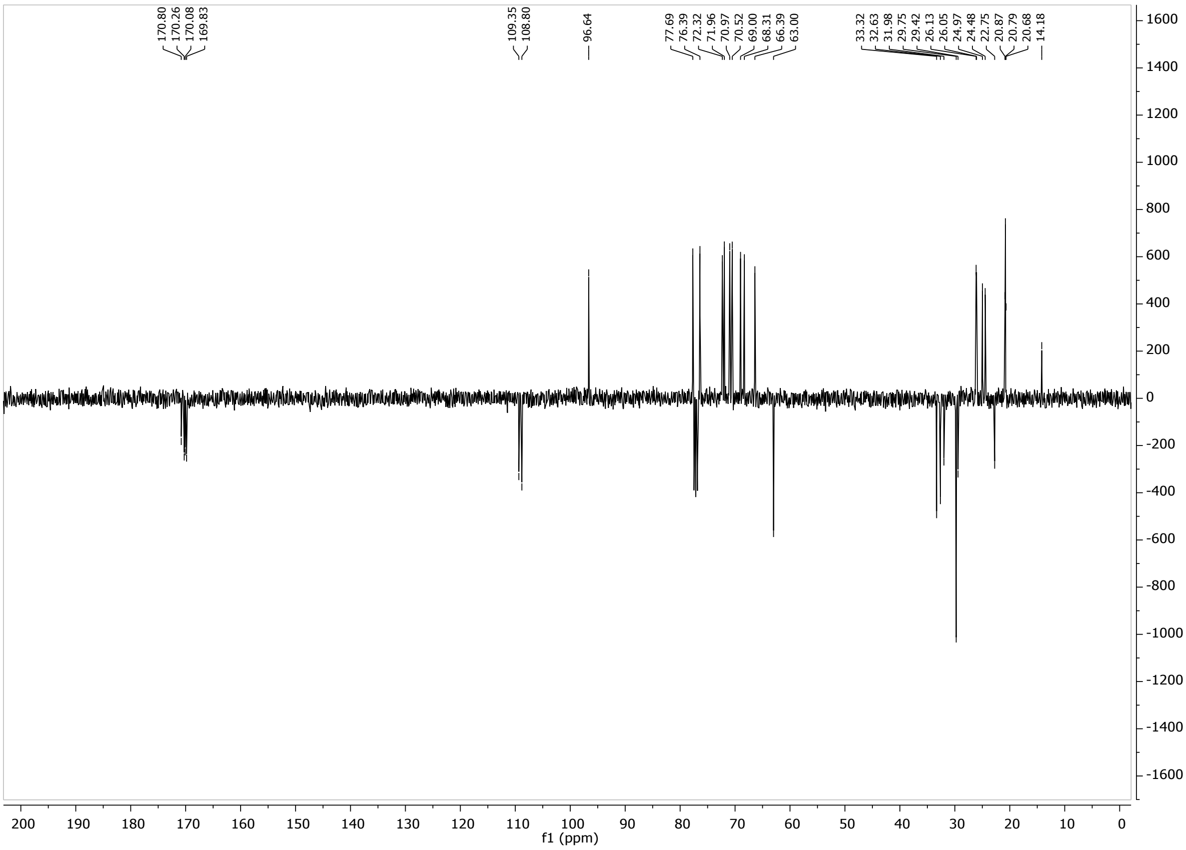

170.80 170.26 170.08 169.83 109.35 108.80 96.64 77.69 76.39 72.32 71.96 70.97 70.52 69.00 68.31 66.39 63.00 33.32 32.63 31.98 29.75 29.42 26.13 26.05 24.97 24.48 22.75 20.87 20.79 20.68 14.18
f1 (ppm)

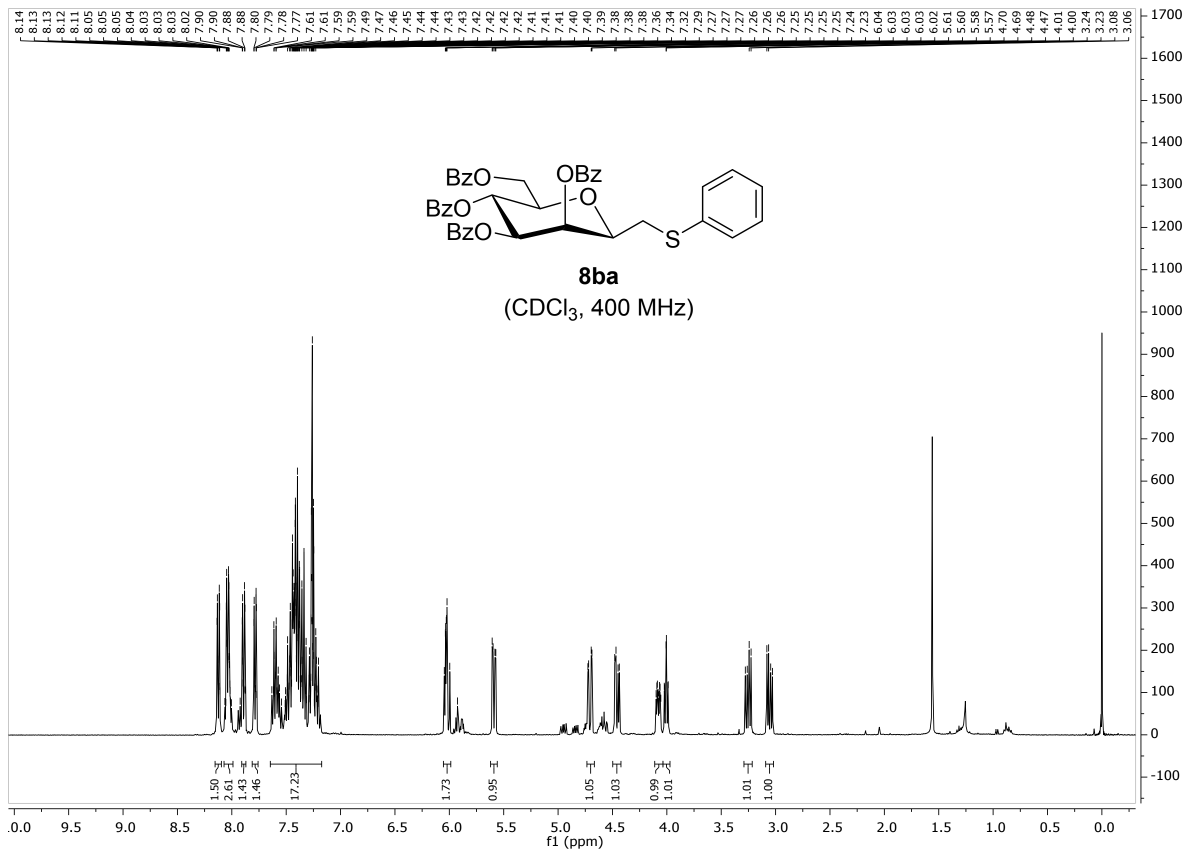
BzO
OBz
BzO
O
BzO
S
8ba
(CDCl3, 400 MHz)
f1 (ppm)

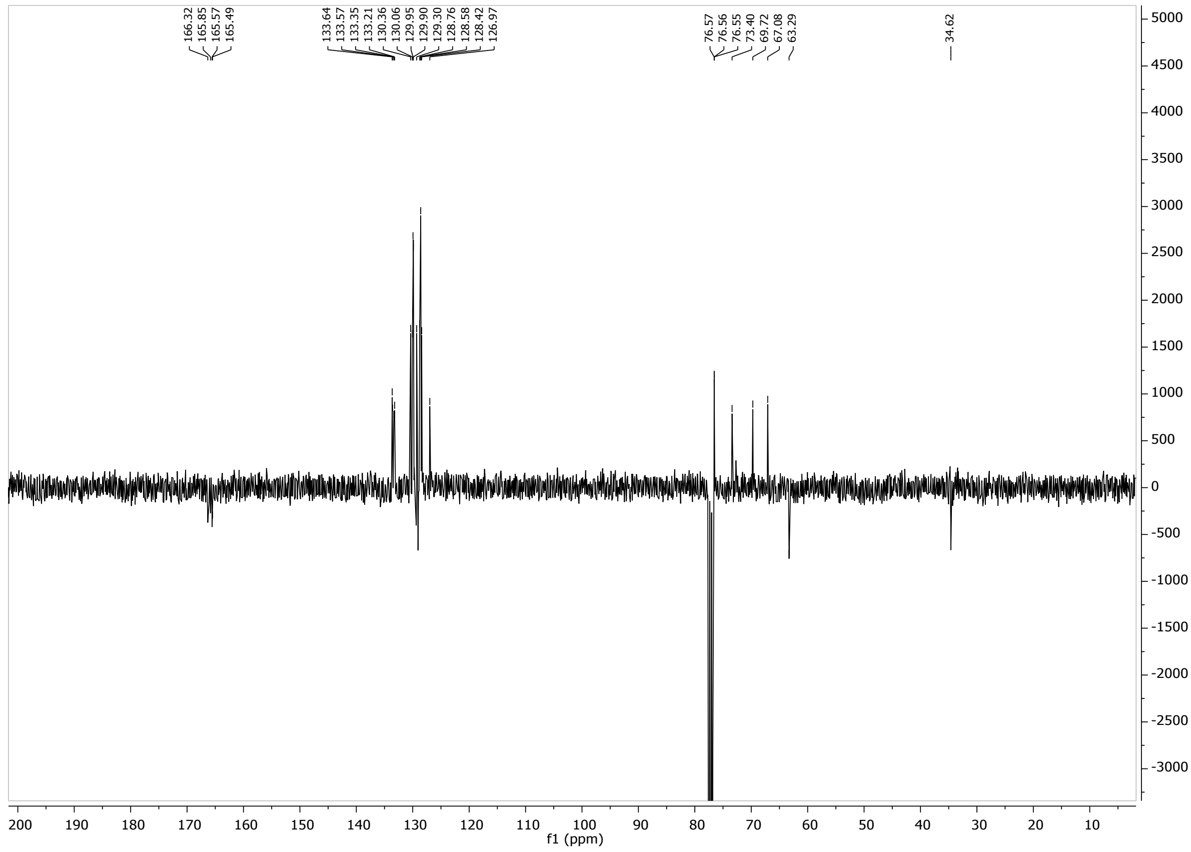
f1 (ppm)

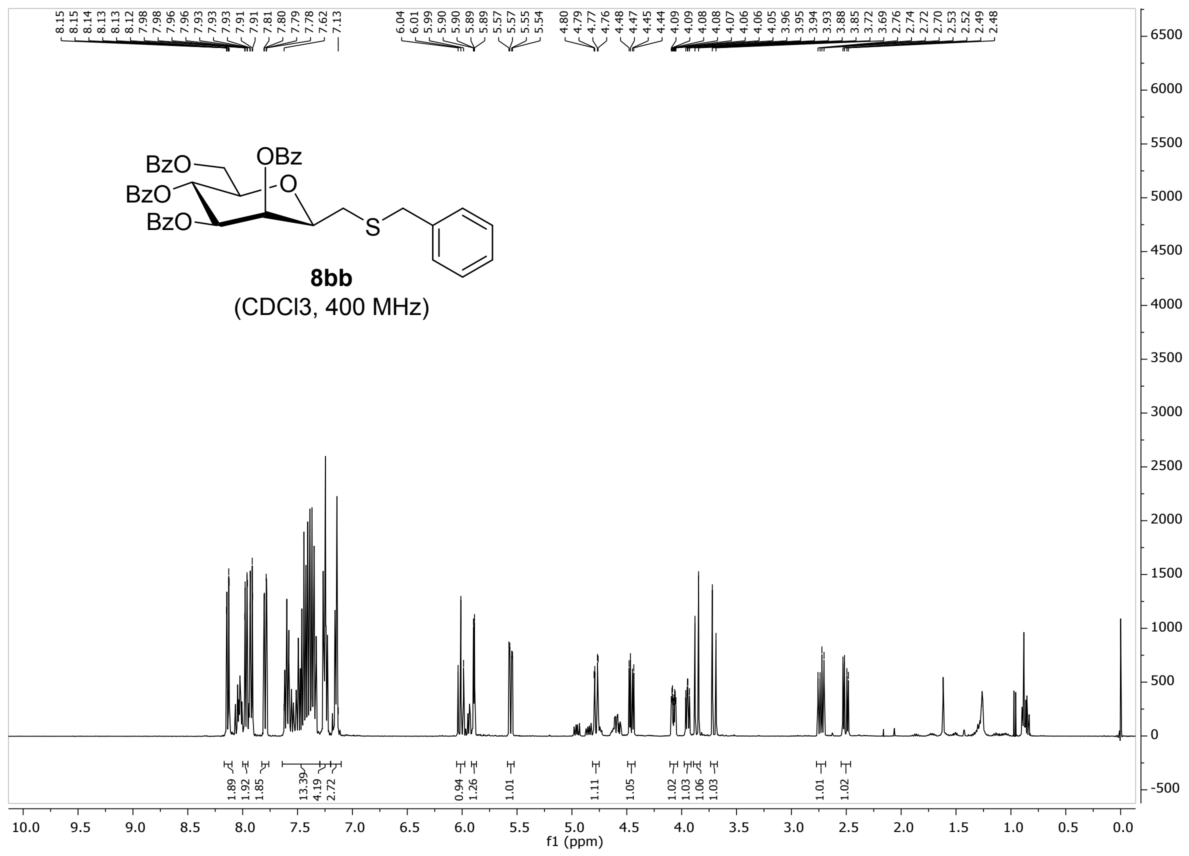
8bb
(CDCl3, 400 MHz)
f1 (ppm)

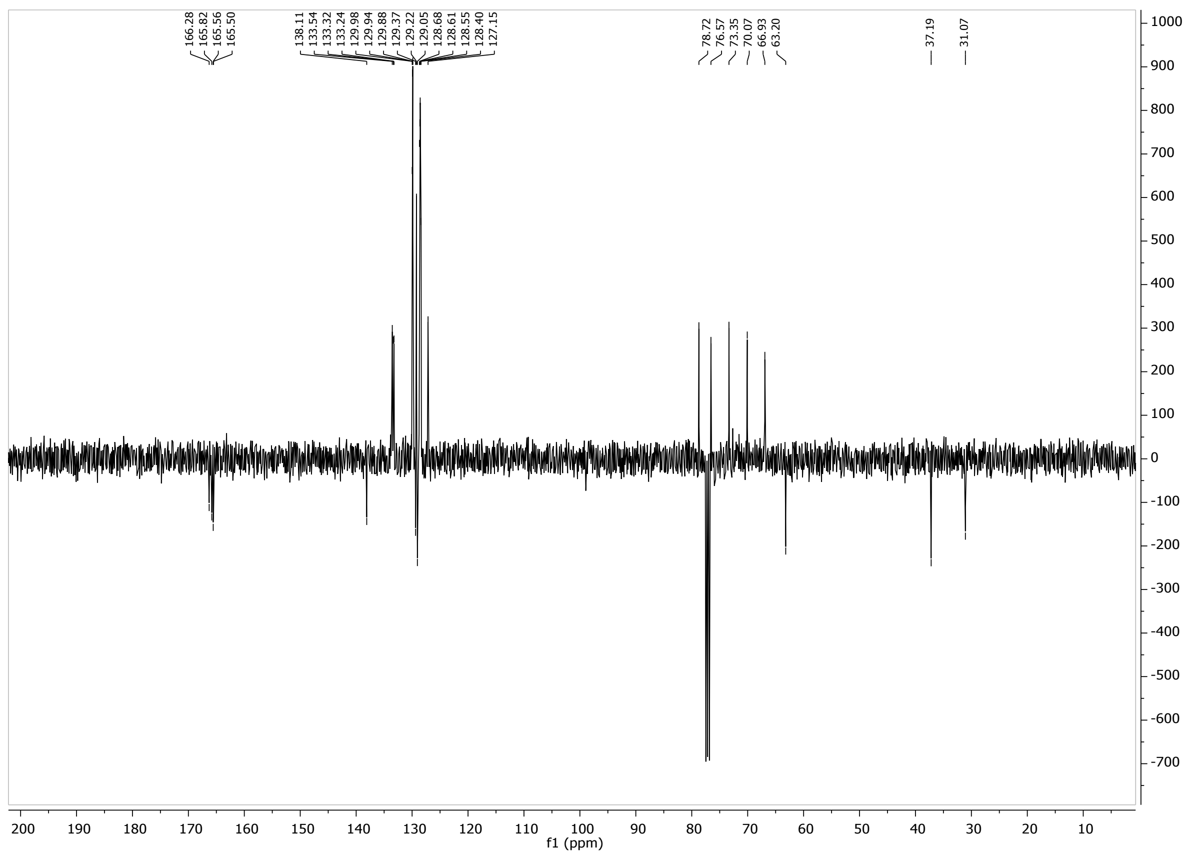
f1 (ppm)

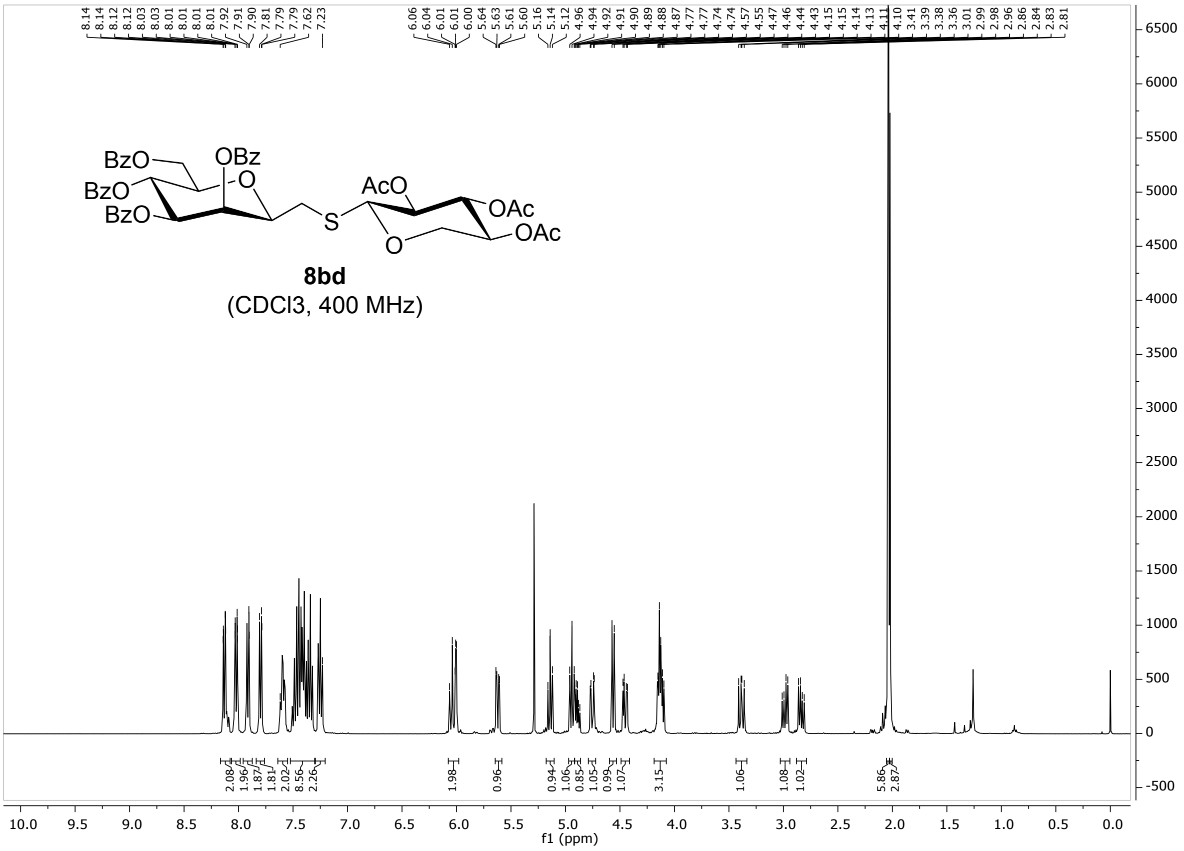
BzO
OBz
BzO
BzO
O
S
AcO
O
OAc
OAc
8bd
(CDCl3, 400 MHz)
f1 (ppm)

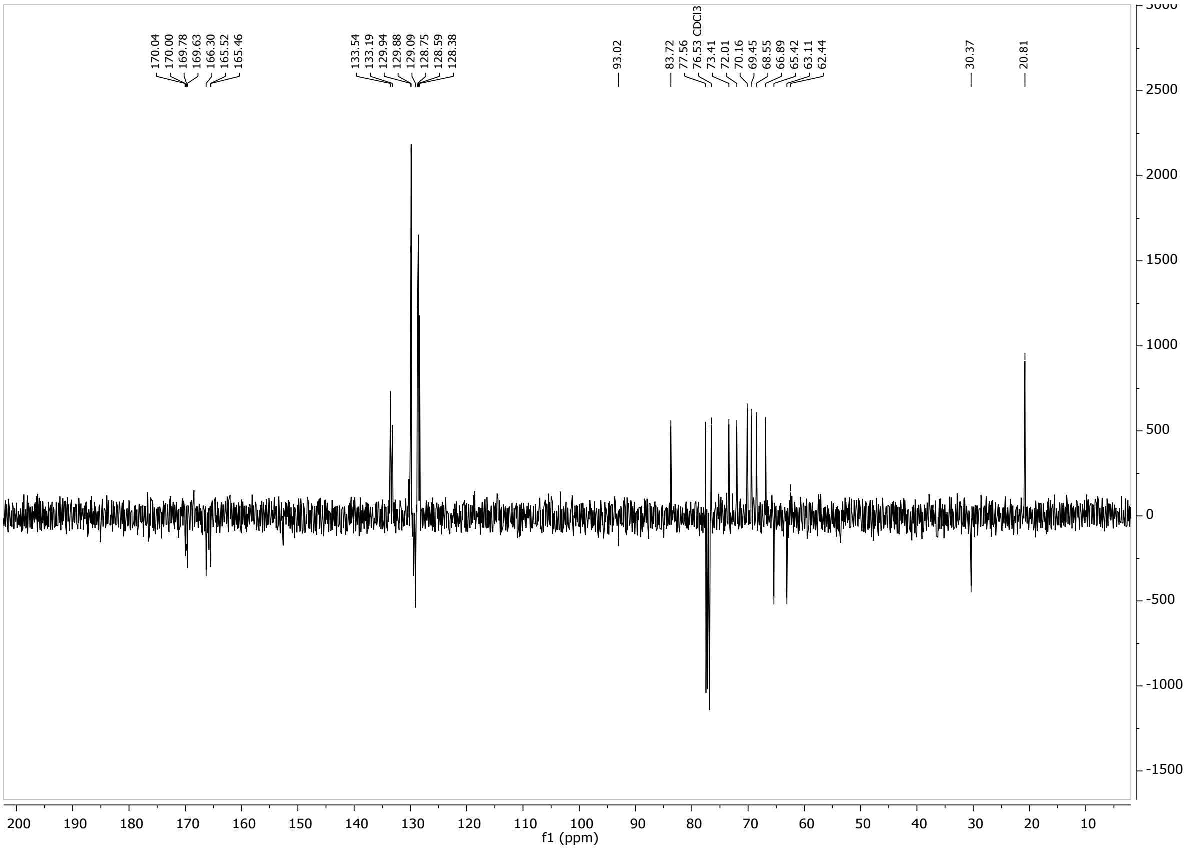
CDCl3
f1 (ppm)

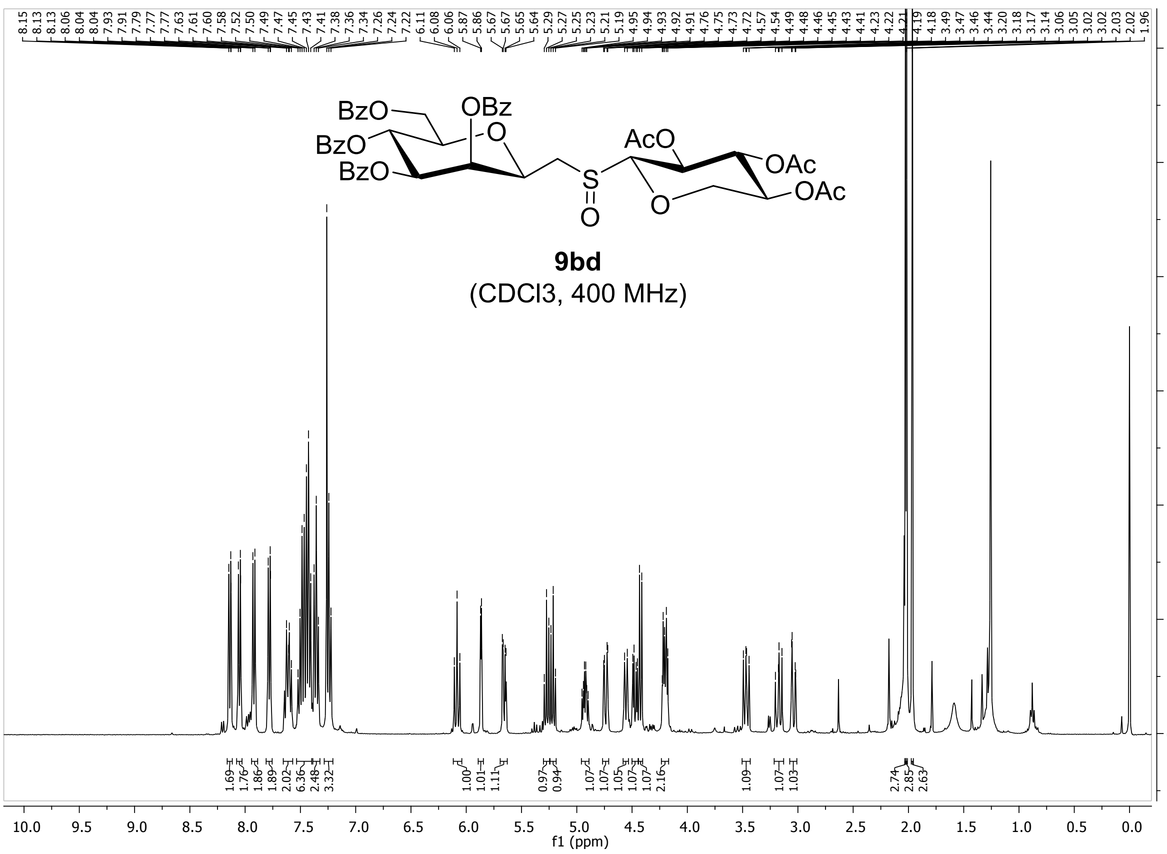
BzO
OBz
O
BzO
BzO
S
O
AcO
OAc
O
OAc
9bd
(CDCl3, 400 MHz)
f1 (ppm)

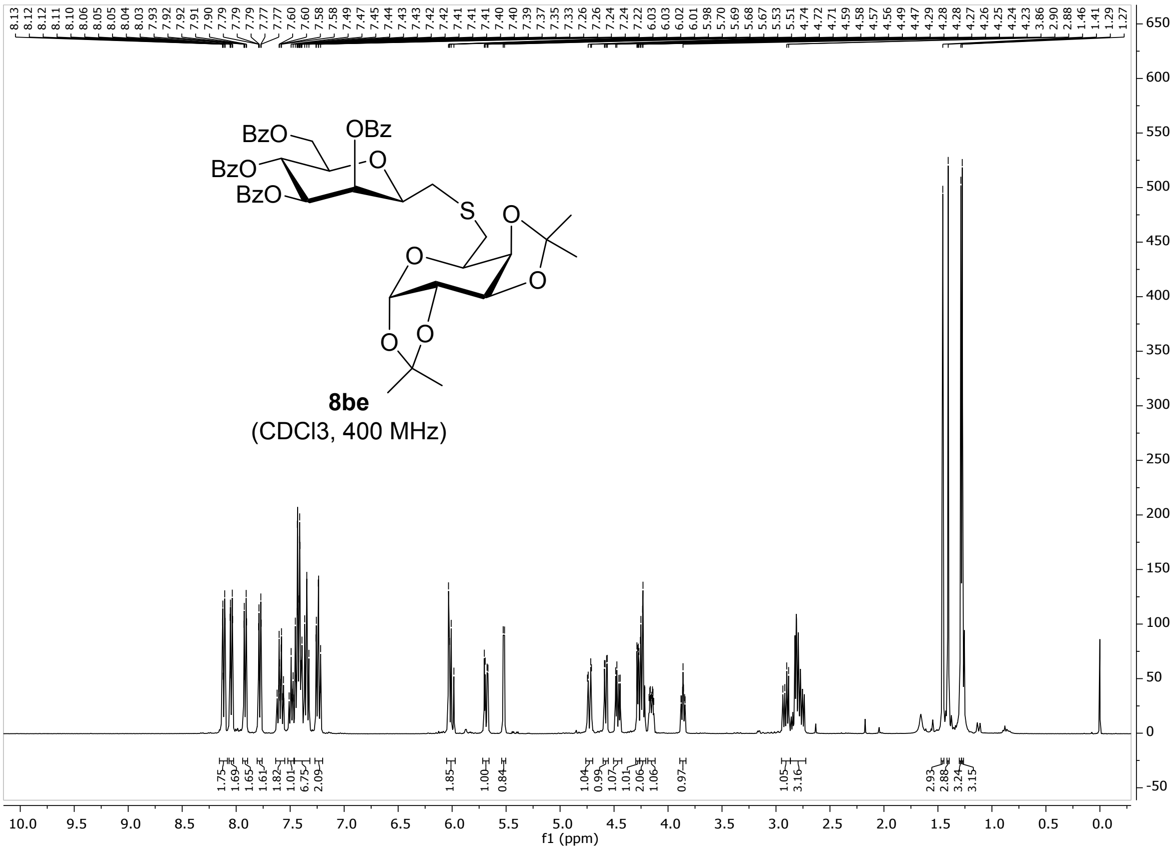
BzO
OBz
O
BzO
BzO
S
O
O
O
O
O
8be
(CDCl3, 400 MHz)
f1 (ppm)

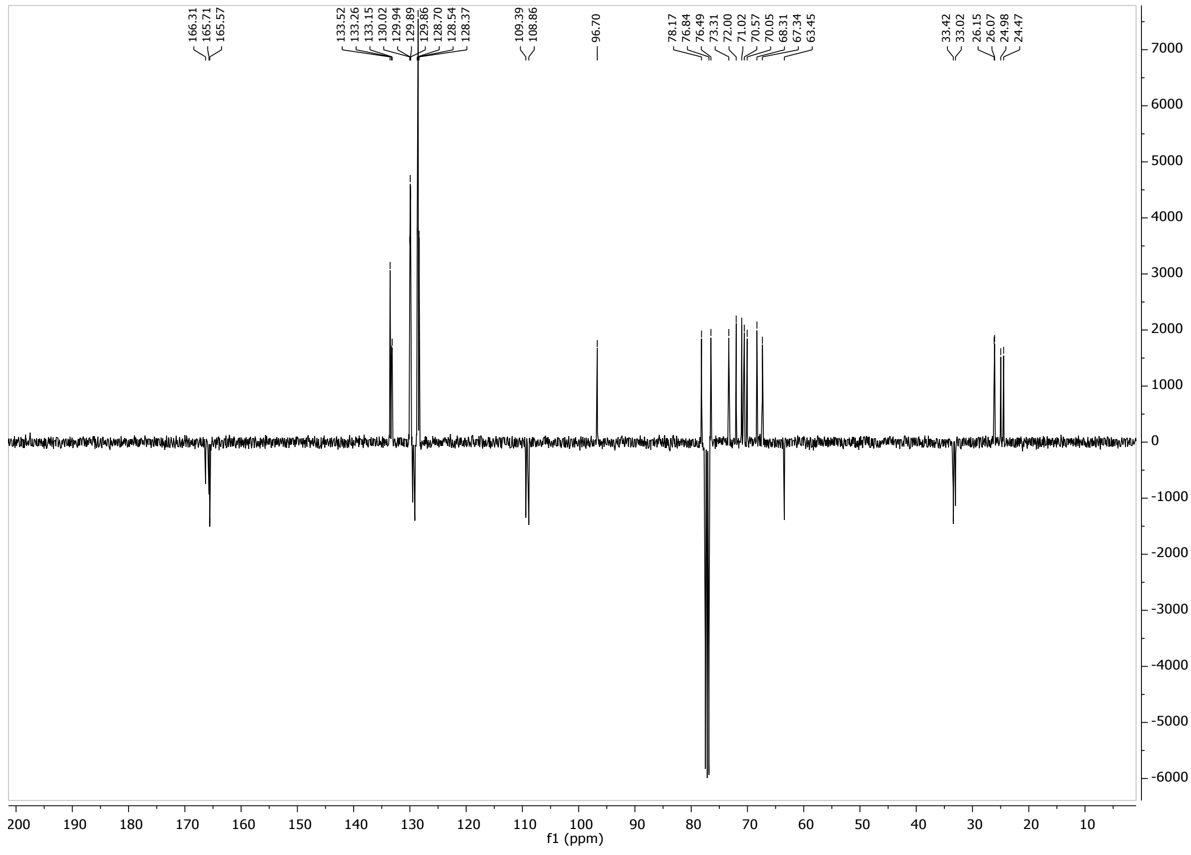
166.31
165.71
165.57
133.52
133.26
133.15
130.02
129.94
129.89
129.86
128.70
128.54
128.37
109.39
108.86
96.70
78.17
76.84
76.49
73.31
72.00
71.02
70.57
70.05
68.31
67.34
63.45
33.42
33.02
26.15
26.07
24.98
24.47
f1 (ppm)

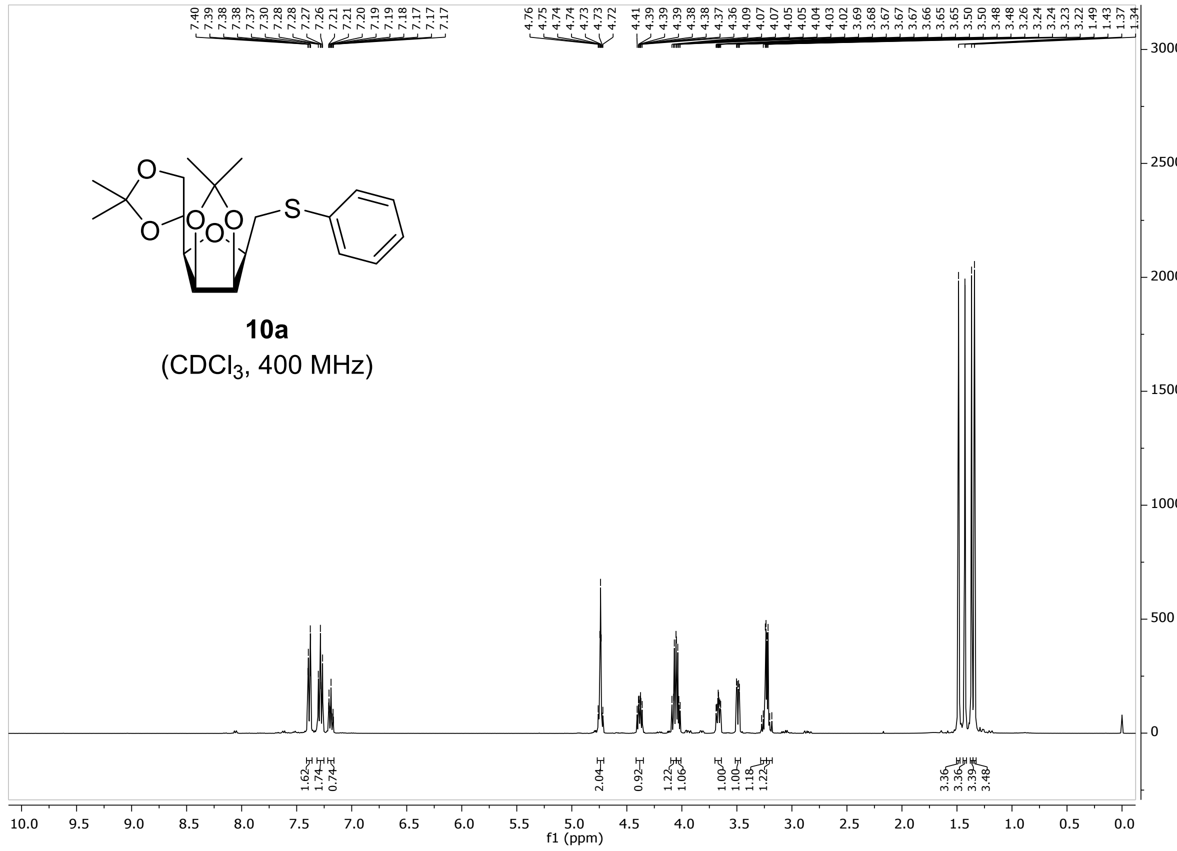
7.40
7.39
7.38
7.37
7.30
7.28
7.28
7.27
7.26
7.21
7.21
7.20
7.19
7.19
7.18
7.17
7.17
7.17
4.76
4.75
4.74
4.74
4.73
4.73
4.72
4.41
4.39
4.39
4.38
4.38
4.37
4.36
4.09
4.07
4.05
4.05
4.04
4.03
4.02
4.02
3.69
3.68
3.67
3.67
3.66
3.65
3.65
3.50
3.50
3.48
3.48
3.26
3.24
3.24
3.23
3.22
1.49
1.43
1.37
1.34
10a
(CDCl3, 400 MHz)
1.62
1.74
0.74
2.04
0.92
1.22
1.06
1.00
1.00
1.18
1.22
3.36
3.36
3.39
3.48
f1 (ppm)

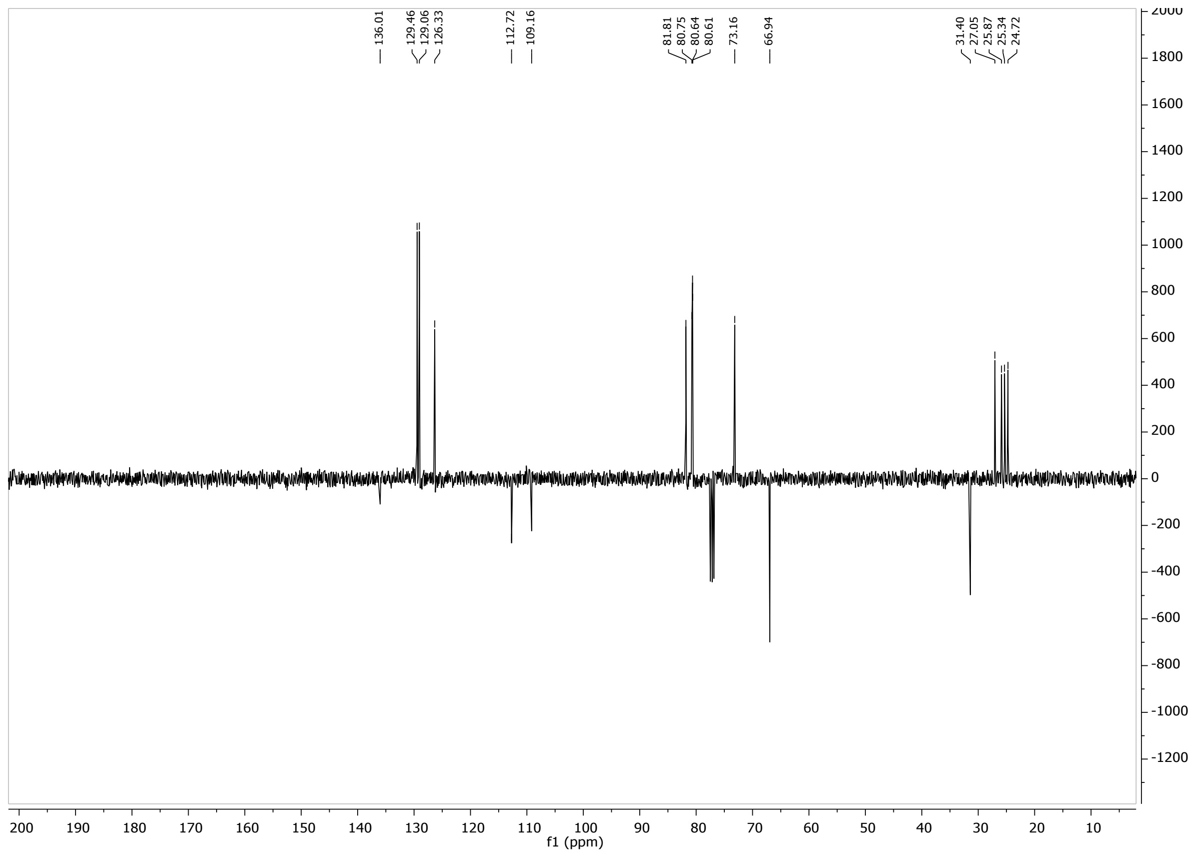
136.01
129.46
129.06
126.33
112.72
109.16
81.81
80.75
80.64
80.61
73.16
66.94
31.40
27.05
25.87
25.34
24.72
f1 (ppm)

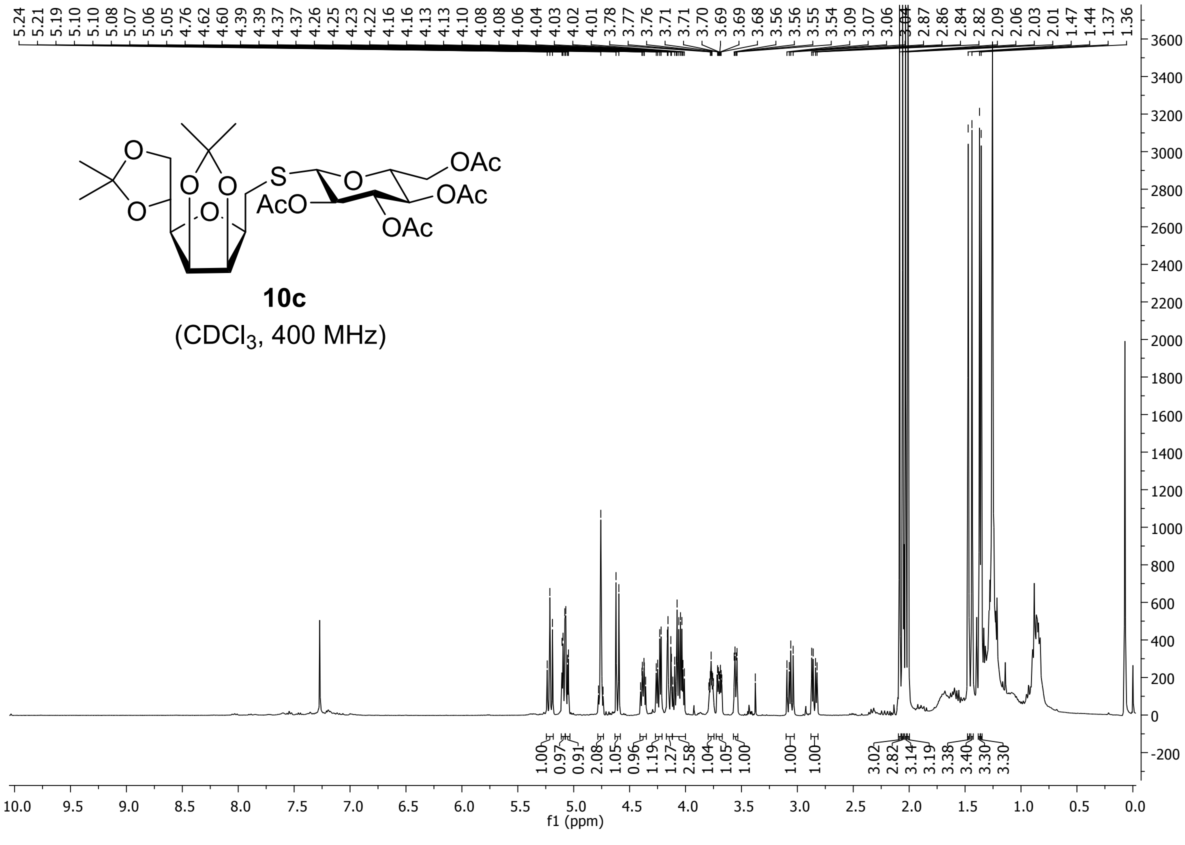
5.24
5.21
5.19
5.10
5.10
5.08
5.07
5.06
5.05
4.76
4.62
4.60
4.39
4.39
4.37
4.37
4.26
4.25
4.23
4.22
4.16
4.16
4.13
4.13
4.10
4.08
4.08
4.06
4.04
4.03
4.02
4.01
3.78
3.77
3.76
3.71
3.71
3.70
3.69
3.69
3.68
3.56
3.55
3.54
3.09
3.07
3.06
3.04
2.87
2.86
2.84
2.82
2.09
2.06
2.03
2.01
1.47
1.44
1.37
1.36
S
O
OAc
AcO
10c
(CDCl3, 400 MHz)
1.00
0.97
0.91
2.08
1.05
0.96
1.19
1.27
2.58
1.04
1.05
1.00
1.00
1.00
3.02
2.82
3.14
3.19
3.38
3.40
3.30
3.30
f1 (ppm)

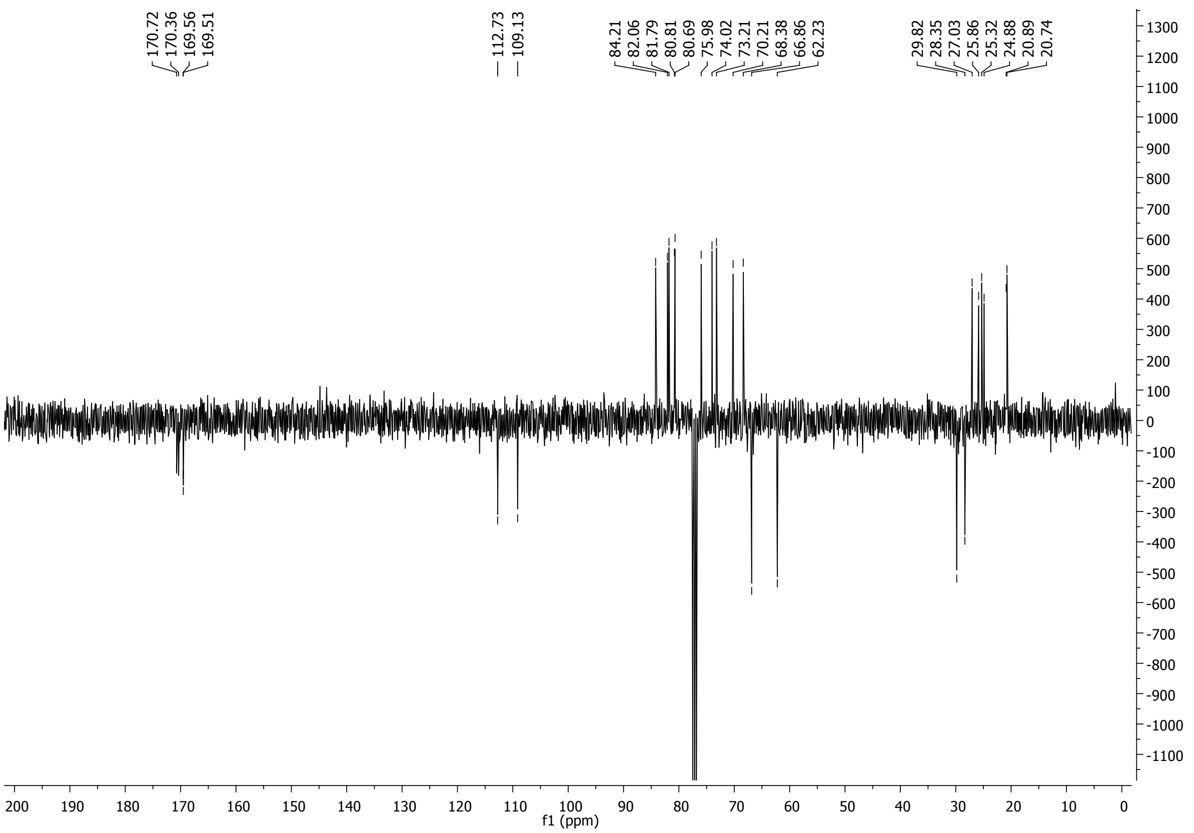

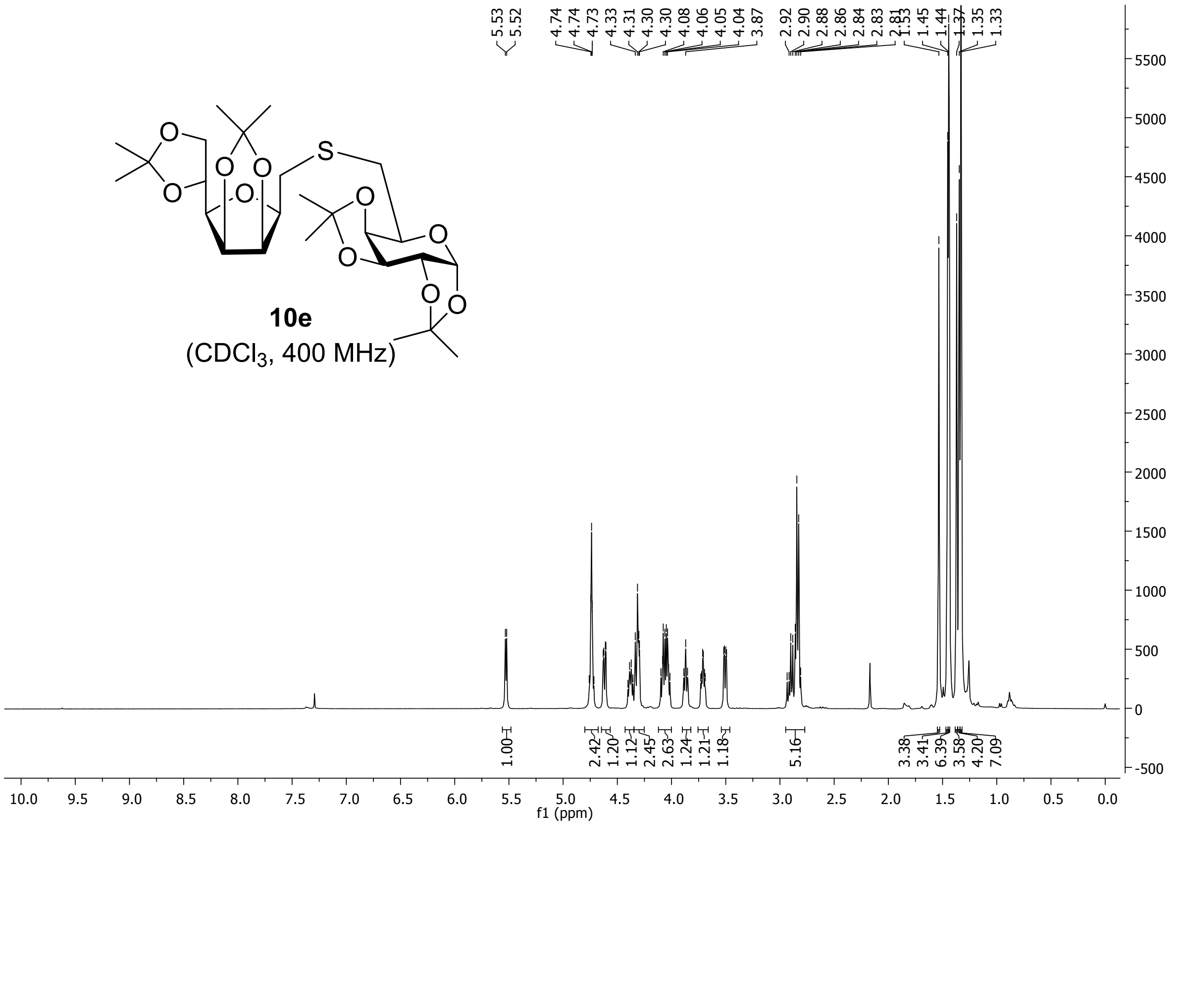

10e
($CDCl_3$, 400 MHz)

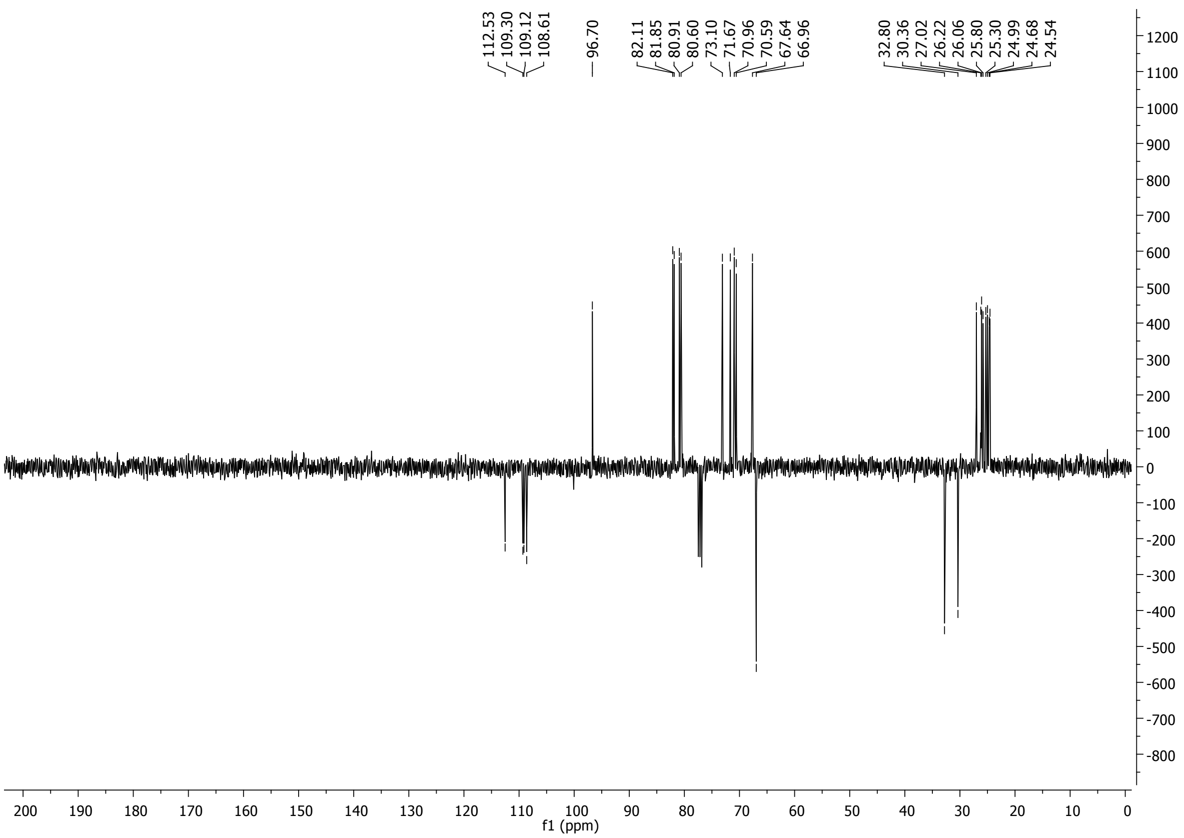

10f
($CDCl_3$, 400 MHz)

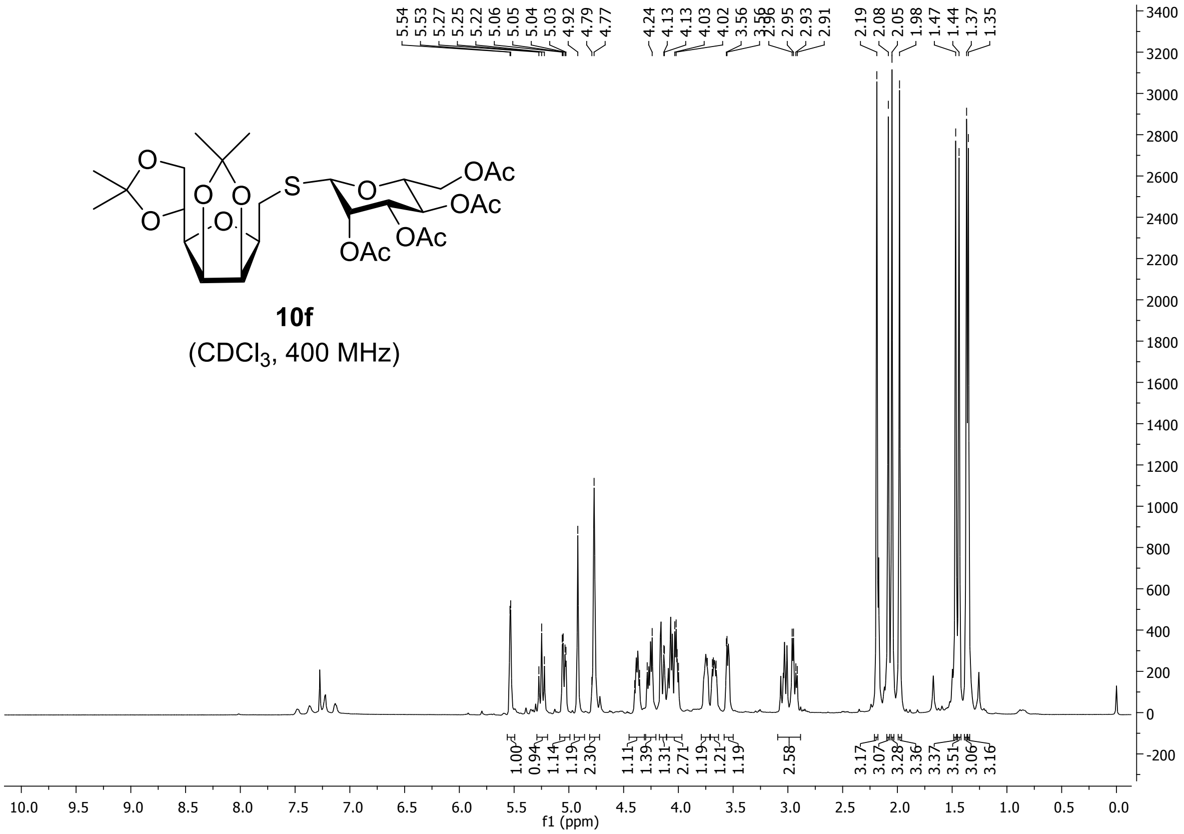

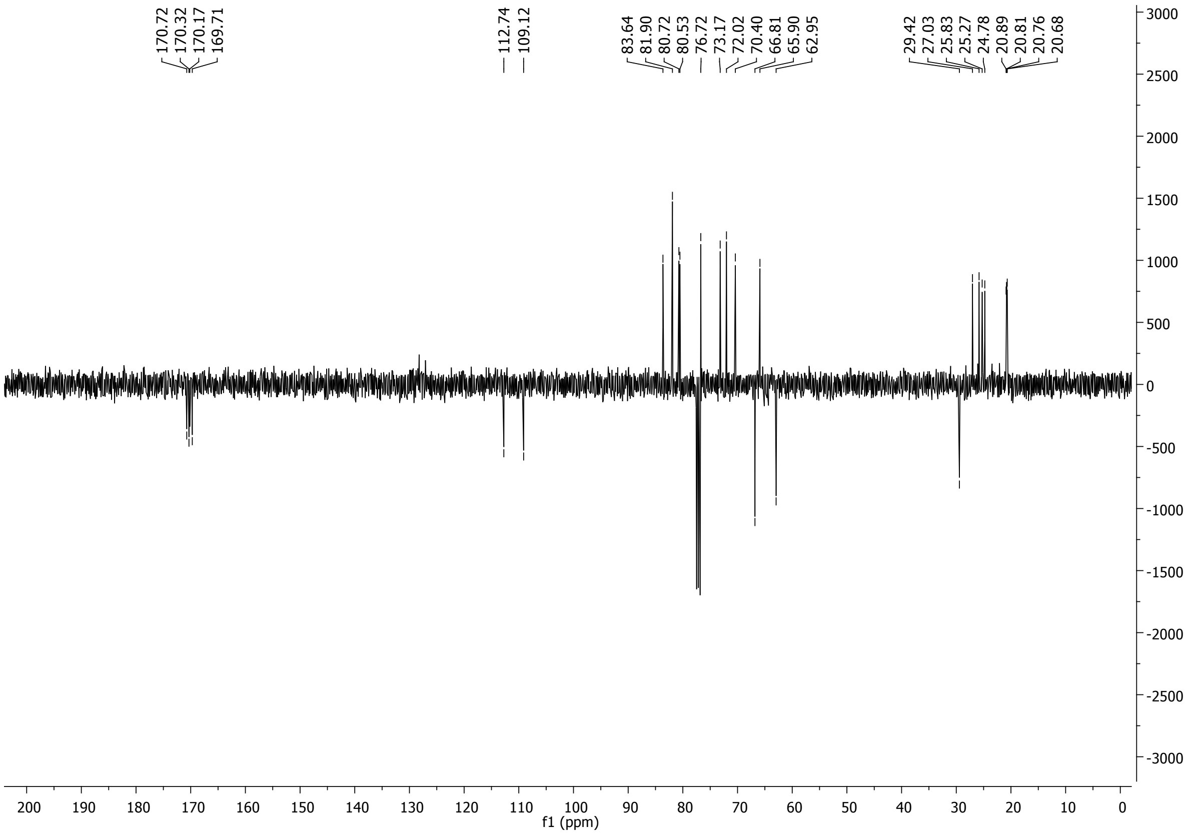
170.72
170.32
170.17
169.71
112.74
109.12
83.64
81.90
80.72
80.53
76.72
73.17
72.02
70.40
66.81
65.90
62.95
29.42
27.03
25.83
25.27
24.78
20.89
20.81
20.76
20.68
f1 (ppm)
